# Construction and comprehensive analysis of a ceRNA network to reveal potential prognostic biomarkers for hepatocellular carcinoma

**DOI:** 10.1186/s12935-019-0817-y

**Published:** 2019-04-11

**Authors:** Junyu Long, Yi Bai, Xiaobo Yang, Jianzhen Lin, Xu Yang, Dongxu Wang, Li He, Yongchang Zheng, Haitao Zhao

**Affiliations:** 1 Department of Liver Surgery, Peking Union Medical College Hospital, Chinese Academy of Medical Sciences & Peking Union Medical College, Beijing, China; 2 Department of Medicine, University of Alabama at Birmingham, Birmingham, AL USA

**Keywords:** Competing endogenous RNA, Hepatocellular carcinoma, Long noncoding RNA, MicroRNA, Prognosis

## Abstract

**Background:**

Long noncoding RNAs (lncRNAs) can act as microRNA (miRNA) sponges to regulate protein-coding gene expression; therefore, lncRNAs are considered a major part of the competitive endogenous RNA (ceRNA) network and have attracted growing attention. The present study explored the regulatory mechanisms and functional roles of lncRNAs as ceRNAs in hepatocellular carcinoma (HCC) and their potential impact on HCC patient prognosis.

**Methods:**

In this study, we systematically studied the expression profiles and prognostic value of lncRNA, miRNA, and mRNA from a total of 838 HCC patients from five HCC cohorts (TCGA, GSE54236, GSE76427, GSE64041 and GSE14520). The TCGA, GSE54236 and GSE76427 HCC cohorts were utilized to establish a prognosis-related network of dysregulated ceRNAs by bioinformatics methods. The GSE64041 and GSE14520 HCC cohorts were utilized to verify the expression of candidate genes.

**Results:**

In total, 721 lncRNAs, 73 miRNAs, and 1563 mRNAs were aberrantly expressed in HCC samples. A ceRNA network including 26 lncRNAs, four miRNAs, and six mRNAs specific to HCC was established. The survival analysis showed that four lncRNAs (MYCNOS, DLX6-AS1, LINC00221, and CRNDE) and two mRNAs (CCNB1 and SHCBP1) were prognostic biomarkers for patients with HCC in both the TCGA and GEO databases.

**Conclusion:**

The proposed ceRNA network may help elucidate the regulatory mechanism by which lncRNAs function as ceRNAs and contribute to the pathogenesis of HCC. Importantly, the candidate lncRNAs, miRNAs, and mRNAs involved in the ceRNA network can be further evaluated as potential therapeutic targets and prognostic biomarkers for HCC.

**Electronic supplementary material:**

The online version of this article (10.1186/s12935-019-0817-y) contains supplementary material, which is available to authorized users.

## Background

Hepatocellular carcinoma (HCC) is a severe cancer with an increasing incidence and is the fifth most prevalent cancer worldwide [1–3]. Viral infections, such as hepatitis B and hepatitis C, are usually associated with liver cirrhosis and HCC tumorigenesis [2, 4]. HCC remains an important global clinical challenge due to its high incidence, limited treatment strategies, and poor prognosis [5]. Therefore, strategies based on a personal need to treat HCC, such as discovering potential biomarkers and therapeutic targets, are urgently needed. The present study explored how HCC-related long noncoding RNAs (lncRNAs) serve as competitive endogenous RNAs (ceRNAs) to regulate target genes and how they affect the pathogenesis and prognosis of HCC.

Although noncoding RNAs (ncRNAs) lack protein-encoding abilities, they are ubiquitous in organisms [6]. As a subtype of ncRNA greater than 200 nucleotides in length, lncRNAs were once considered transcriptional noise. Several studies have indicated that lncRNAs have many pivotal functions in tumor-related processes, including proliferation, invasion, and metastasis [7–9]. However, verification of the regulatory roles of lncRNAs in gene expression remains difficult. Currently, many researchers are working to reveal the different biological functions of lncRNAs in malignant tumors.

One ceRNA hypothesis was proposed by Salmena et al. [10]. A complicated posttranscriptional regulatory network was described that allowed lncRNAs, mRNAs, and other RNAs to compete with microRNAs (miRNAs) via acting as natural miRNA sponges by virtue of sharing no less than one miRNA response element (MRE). These ncRNAs act as ceRNAs to modulate mRNA expression and regulate protein levels, which contributes to the occurrence and development of tumors [11, 12]. Studies have shown that each miRNA can control the transcriptional expression levels of hundreds of proteins, and each mRNA contains different MREs and thus may be targeted by multiple miRNAs [12].

Recently, a growing number of studies have verified that the lncRNA–miRNA–mRNA regulatory network plays a critical role in the progression and pathogenesis of several tumors, including liver cancer, gallbladder cancer, and other malignant tumors [13–15]. lncRNAs with sequences similar to their target miRNAs can separate miRNAs from mRNAs. Wang et al. demonstrated that the lncRNA HULC influenced PRKACB gene expression by competitively combining with the miRNA miR-372 and thus participated in liver cancer pathogenesis [13]. Wang et al. confirmed that lncRNA H19 acts as a molecular sponge to absorb miR-342-3p in gallbladder cancer and upregulate FOXM1 gene expression [15].

Therefore, lncRNAs acting as ceRNAs have diverse biological functions that deserve further exploration. Here, we investigated differences in RNA expression patterns between 43 HCC tumor tissues and 43 paired nontumorous tissues and constructed a ceRNA network associated with HCC, including 26 lncRNAs, four miRNAs, and six mRNAs. The survival analysis showed that four lncRNAs (MYCNOS, DLX6-AS1, LINC00221, and CRNDE) and two mRNAs (CCNB1 and SHCBP1) were prognostic biomarkers for patients with HCC in both the Cancer Genome Atlas (TCGA) and Gene Expression Omnibus (GEO) databases. These candidate genes involved in the ceRNA network may become potential therapeutic targets or diagnostic biomarkers for HCC.

## Materials and methods

### Study population

A total of 371 patients with HCC participated in this study. Available miRNA-seq data from 371 HCC samples with survival data and 50 adjacent nontumorous samples (including 50 paired HCC samples) and mRNA-seq data for 367 HCC samples with survival data and 50 adjacent nontumorous samples (including 50 paired HCC samples) were obtained from the TCGA database (https://tcga-data.nci.nih.gov/) on May 1, 2018. Approval from the ethics committee was not necessary. This study fully meets the publication requirements of the TCGA. The RNA-seq data were obtained using the Illumina HiSeq_miRNA-Seq and Illumina HiSeq_RNA-Seq platforms. The genes identified via RNA expression profiling were annotated based on the Ensembl Gene ID. Log_2_ transformation was performed on all gene expression data. We normalized the downloaded data by using the trimmed mean of M value (TMM) normalization method of the edgeR R package (Version: 3.24.3) in R software (Version: 3.5.2) [16]. When an RNA had duplicate data, the average RNA expression was used. The lncRNAs, miRNAs and mRNAs with an average expression value > 1 were retained, and low-abundance RNAs were eliminated.

### Microarray data

The gene expression profiles from GSE54236 (including 77 adjacent nontumorous samples and 78 HCC samples; platform: GPL6480), GSE76427 (including 52 adjacent nontumorous samples and 115 HCC samples; platform: GPL10558), GSE64041 (including 60 HCC samples and 60 paired adjacent nontumorous samples; platform: GPL6244), and GSE14520 (including 214 HCC samples and 214 paired adjacent nontumorous samples; platform: GPL571) were obtained from the GEO database (https://www.ncbi.nlm.nih.gov/geo/). Log_2_ transformation was performed on only the gene expression data for GSE76427. The average RNA expression value was used when duplicated data were found. The genes with an average expression value > 1 were retained, and low-abundance microarray data were removed. Two datasets (GSE54236 and GSE76427) with clinical data were integrated into the meta-GEO HCC cohort to identify the candidate genes associated with the overall survival of patients with HCC. We remove batch effects by using the sva package, and normalized the data by using the scale method in the limma R package (Version: 3.38.3) [17]. The GSE64041 and GSE14520 HCC cohorts with paired HCC samples were utilized to verify the expression patterns of the candidate genes. The paired t-test was used for normally distributed data; otherwise, the Wilcoxon rank test for paired data was used to evaluate whether a gene was differentially expressed between normal tissue and tumor tissue.

### Identification of differentially expressed genes (DEGs)

Ensembl genome browser 92 (http://asia.ensembl.org/index.html) was used to annotate gene symbols. Then, we compared 50 HCC samples with 50 paired nontumorous samples to identify differentially expressed lncRNAs (DElncRNAs), mRNAs (DEmRNAs), and miRNAs (DEmiRNAs) utilizing the edgeR R package with the threshold set at an adjusted P-value < 0.01 and |log_2_-fold change (FC)| > 2 [16]. A hierarchical cluster heatmap based on Euclidean distance was generated using the pheatmap R package (Version: 1.0.12) and represents the expression intensity and direction of DEGs.

### Constructing the ceRNA network

To ensure the functions of the lncRNAs, miRNAs, and mRNAs in the ceRNA network and to further improve the ceRNA network reliability, a coexpression network of DElncRNAs, DEmiRNAs, and DEmRNAs was constructed; the ggalluvial R package (Version: 0.9.1) was used to visualize the ceRNA network [18]. Using the miRcode database (Version 11; http://www.mircode.org/), we confirmed the interactions between DElncRNAs and DEmiRNAs. miRcode, which is an integrated, searchable map of putative target sites of miRNAs, includes the complete transcriptome annotated by the ENCyclopedia Of DNA Elements (ENCODE) [19]. The existing version contains 10,419 lncRNA genes. DEmRNAs targeted by the DEmiRNAs were retrieved from the miRDB (Version 5.0; http://mirdb.org), miRTarBase (Version 7.0; http://mirtarbase.mbc.nctu.edu.tw/), and TargetScan (Version 7.2; http://www.targetscan.org/vert_72/) databases [20–22]. miRDB refers to an online database used to predict miRNA targets and make functional annotations [20]. MirTarget is a bioinformatics tool that was developed based on the analysis of thousands of interactions among miRNA targets from next-generation sequencing experiments [20] and was used to predict all targets in the miRDB database [20]. The DEmRNAs targeted by DEmiRNAs were verified by experimental studies using reporter assays, qRT-PCR, microarray analysis, Western blotting, and high-throughput sequencing experiments reported in miRTarBase [21]. TargetScan was used to search for conserved 6-mer, 7-mer, and 8-mer sites that corresponded to the seed regions of every miRNA to complete the prediction of biological targets of miRNAs [22].

### Survival analysis

After combining the overall survival of patients with HCC in the TCGA, the survival R package (Version: 2.43-3) was used to perform a survival analysis of the samples with DElncRNAs, DEmiRNAs, and DEmRNAs to identify the prognostic genes. The patients were classified into two groups (high vs. low) using optimal cut-off values determined by the survminer R package (Version: 0.4.3). Log-rank P < 0.05 was considered significant.

### Gene set enrichment analysis (GSEA)

Based on the expression level of the candidate gene and the median expression value, 43 HCC samples from the RNA-seq data of the TCGA HCC cohort were classified into the low-expression and high-expression groups. To identify the underlying function of candidate genes, GSEA (Version: 3.0; http://software.broadinstitute.org/gsea/index.jsp) was conducted between the two groups [23]. The annotated gene sets file c5.bp.v6.2.symbols.gm was chosen for the reference gene sets. Gene size ≥ 100, |enrichment score (ES)| > 0.6 and false discovery rate (FDR) < 0.01 were set as the cut-off criteria.

## Results

### DEGs in HCC

**Fig. 1:**
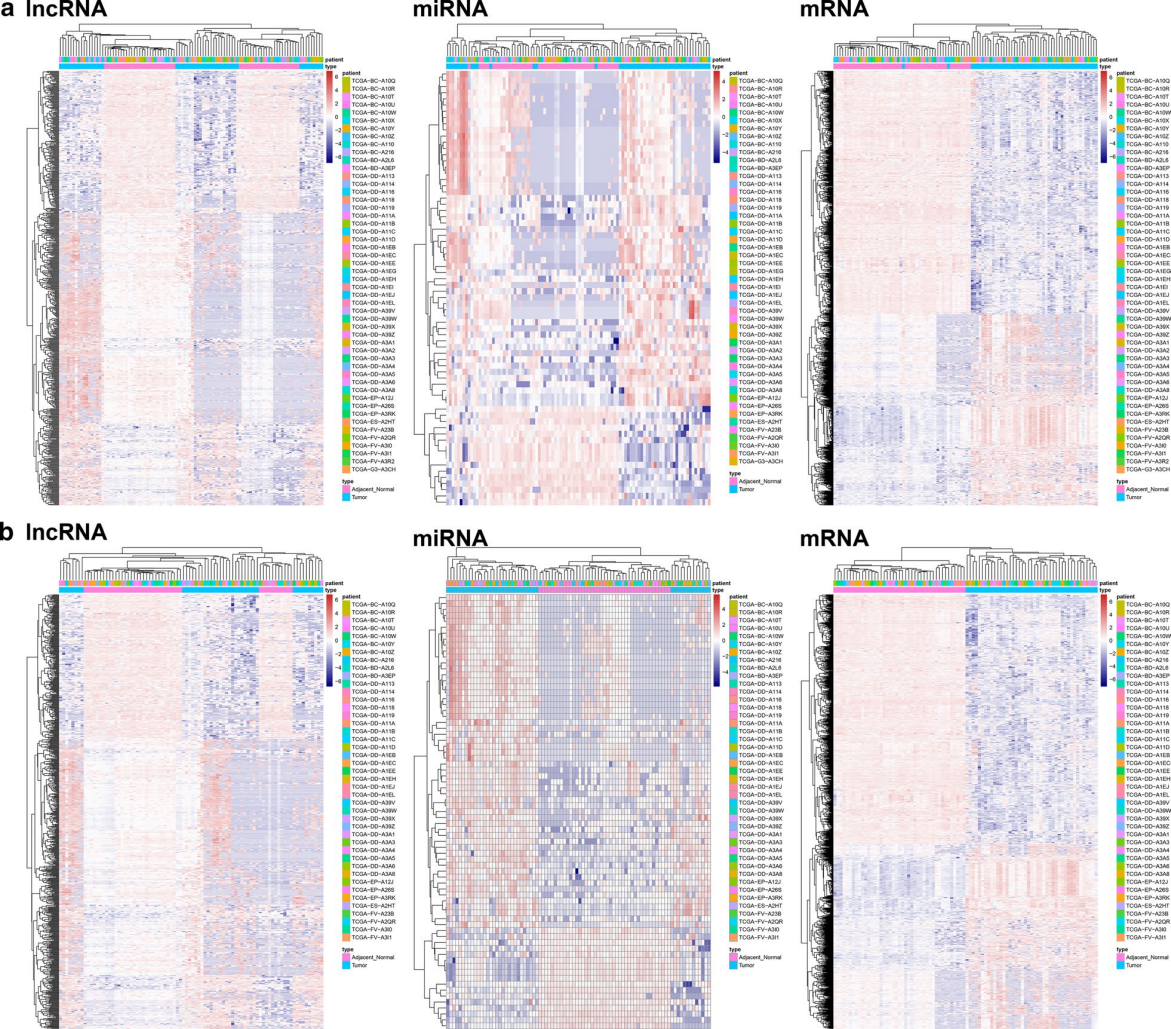
Heatmap plots of the differentially expressed genes (DEGs). Heatmap plots of the differentially expressed lncRNAs, miRNAs, and mRNAs in the 50 paired HCC samples (**a**) and 43 paired HCC samples (**b**). The left vertical axis presents the DEG clusters. The horizontal axis represents the samples. Sample clusters are presented above the horizontal axis. The vertical axis presents the DEGs. Blue denotes downregulated genes, and red denotes upregulated genes

Using a cut-off threshold of |log_2_ FC| > 2 and an adjusted P-value < 0.01 for the 50 HCC tissues compared with the paired 50 nontumorous samples, we identified 641 DElncRNAs, 70 DEmiRNAs, and 1392 DEmRNAs. Then, we used those DEGs for cluster analysis and removed seven abnormal samples (Fig. 1a). Next, we used the remaining 43 HCC tissues and the 43 paired nontumorous samples to perform differential expression analyses. As a result, 721 DElncRNAs, 73 DEmiRNAs, and 1563 DEmRNAs were identified (Additional file 1: Table S1). The heatmap of the lncRNAs, miRNAs and mRNAs showed that the tumors clustered separately from the paired nontumorous tissues (Fig. 1b).

### Construction of a ceRNA network for HCC

**Fig. 2:**
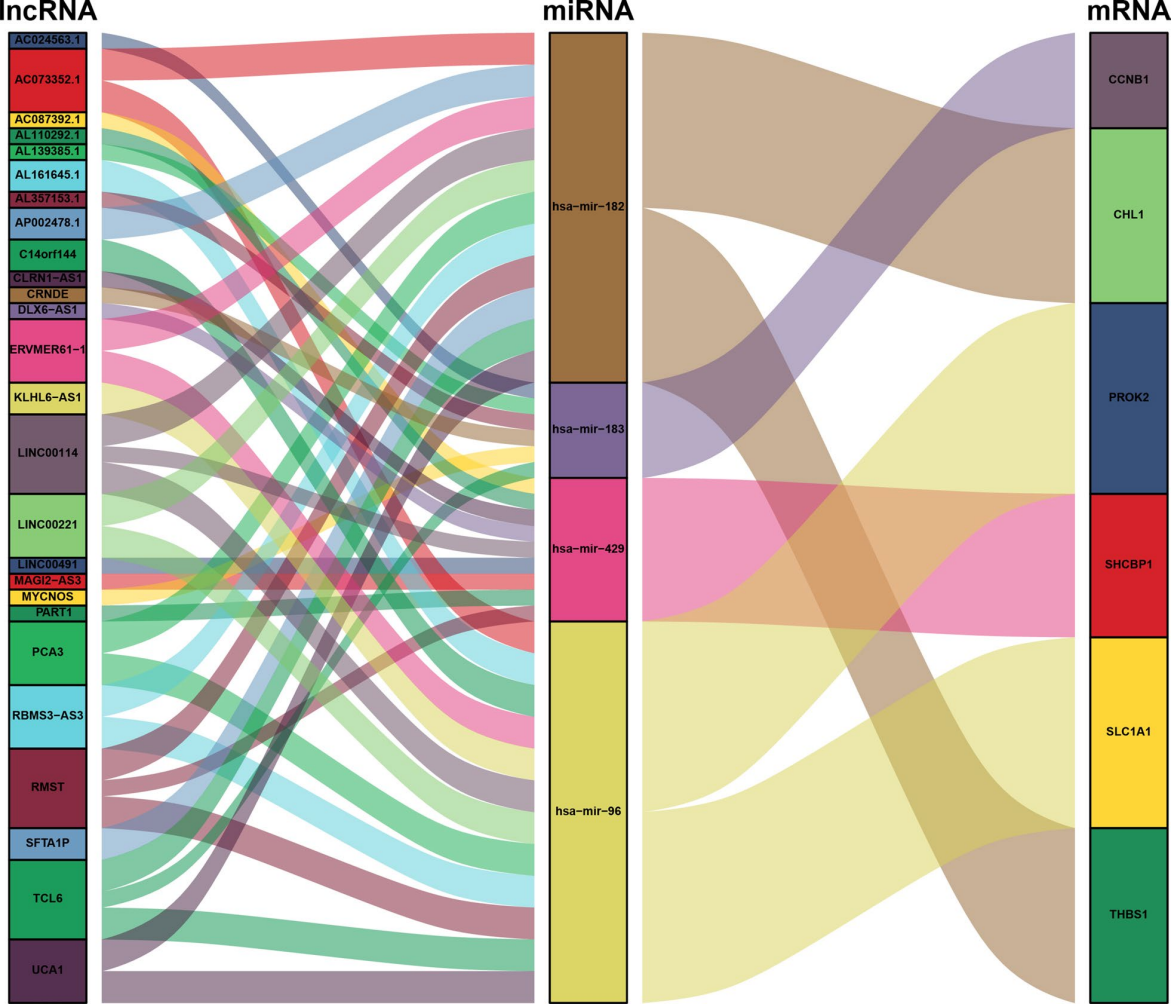
Sankey diagram for the ceRNA network in HCC. Each rectangle represents a gene, and the connection degree of each gene is visualized based on the size of the rectangle

To better understand the effect of lncRNAs on mRNAs mediated by combination with miRNAs in HCC, we built a ceRNA network based on the abovementioned data and used the ggalluvial R package (Version: 0.9.1) to visualize the network. Additional file 2: Table S2 shows that 44 DElncRNAs interact with nine DEmiRNAs retrieved from the miRcode database. Eight of these nine DEmiRNAs were identified in the starBase database. Then, we searched for DEmRNAs based on eight DEmiRNAs in the miRDB, miRTarBase, and TargetScan databases. Six DEmRNAs that can interact with four of the eight DEmiRNAs according to all three of the databases were chosen (Additional file 3: Table S3). After removing the remaining four DEmiRNAs and the corresponding lncRNAs, 26 DElncRNAs, four DEmiRNAs, and six DEmRNAs were used to establish a ceRNA network (Fig. 2) (Additional file 4: Table S4). Furthermore, the connection degree of each gene by topology was calculated to illustrate its importance in the ceRNA network (Fig. 2) (Additional file 5: Table S5).

### Prognosis assessment

**Fig. 3:**
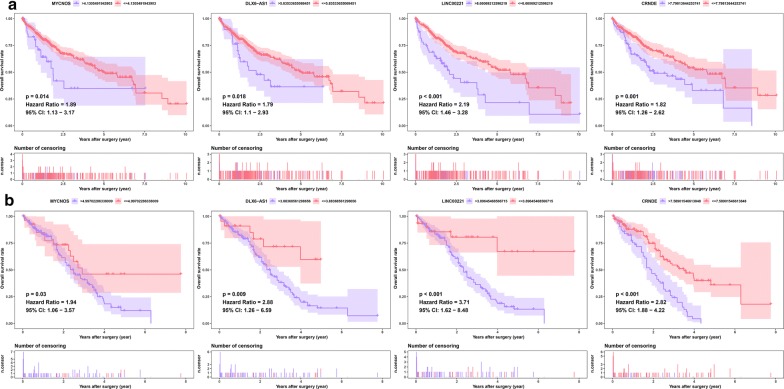
Survival analysis for DElncRNAs. Kaplan–Meier survival curves for DElncRNAs in the TCGA (**a**) and meta-GEO (**b**) HCC cohorts. The horizontal axis indicates the overall survival time in years, and the vertical axis indicates the survival rate

**Fig. 4:**
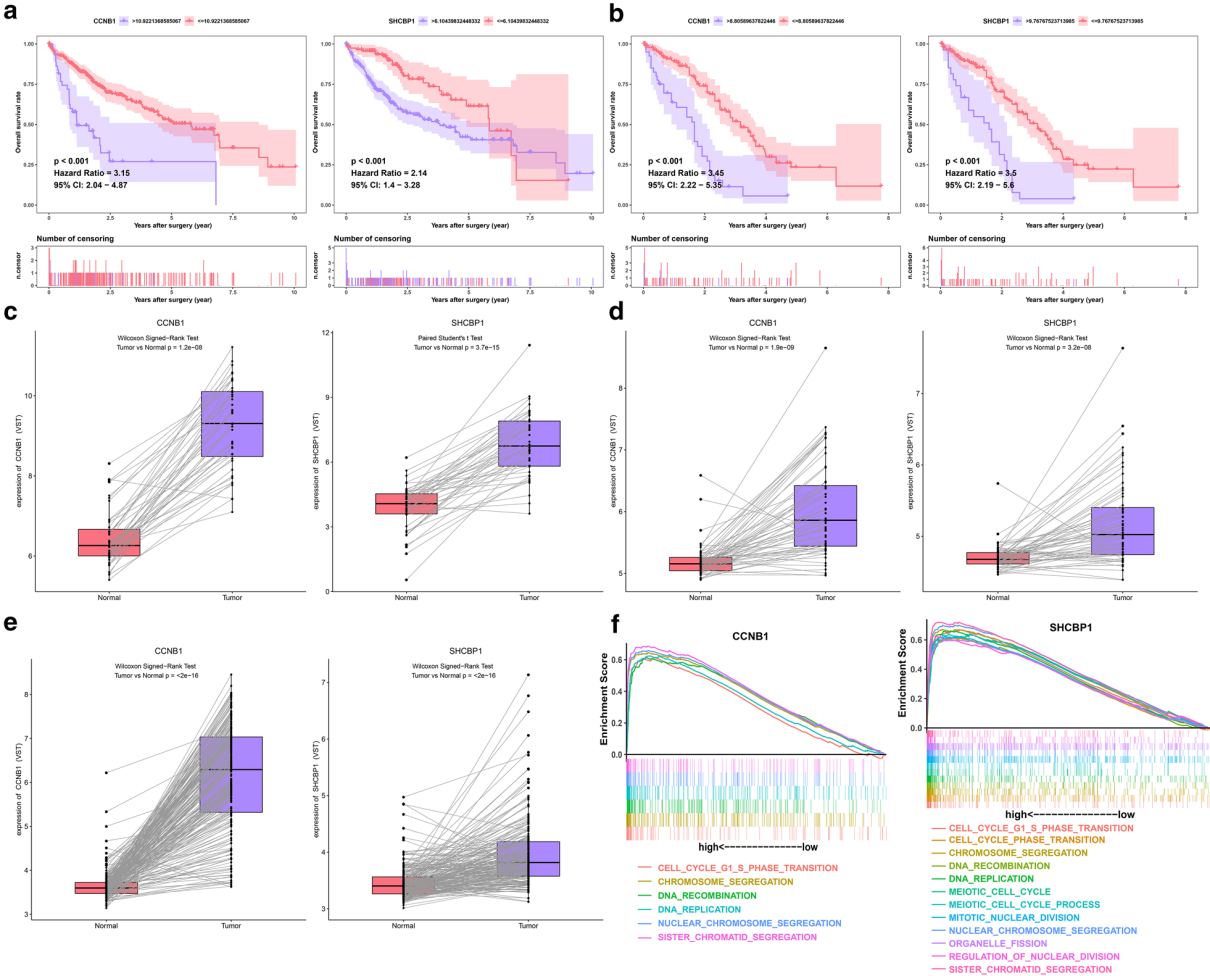
Survival analysis for DEmiRNAs. Kaplan–Meier survival curves for DEmRNAs in the TCGA (**a**) and meta-GEO (**b**) HCC cohorts. The horizontal axis indicates the overall survival time in years, and the vertical axis indicates the survival rate. The expression pattern of DEmRNAs in the TCGA (**c**), GSE64041 (**d**) and GSE14520 (**e**) HCC cohorts of paired HCC samples. Gene set enrichment analysis of DEmRNAs (**f**)

**Fig. 5:**
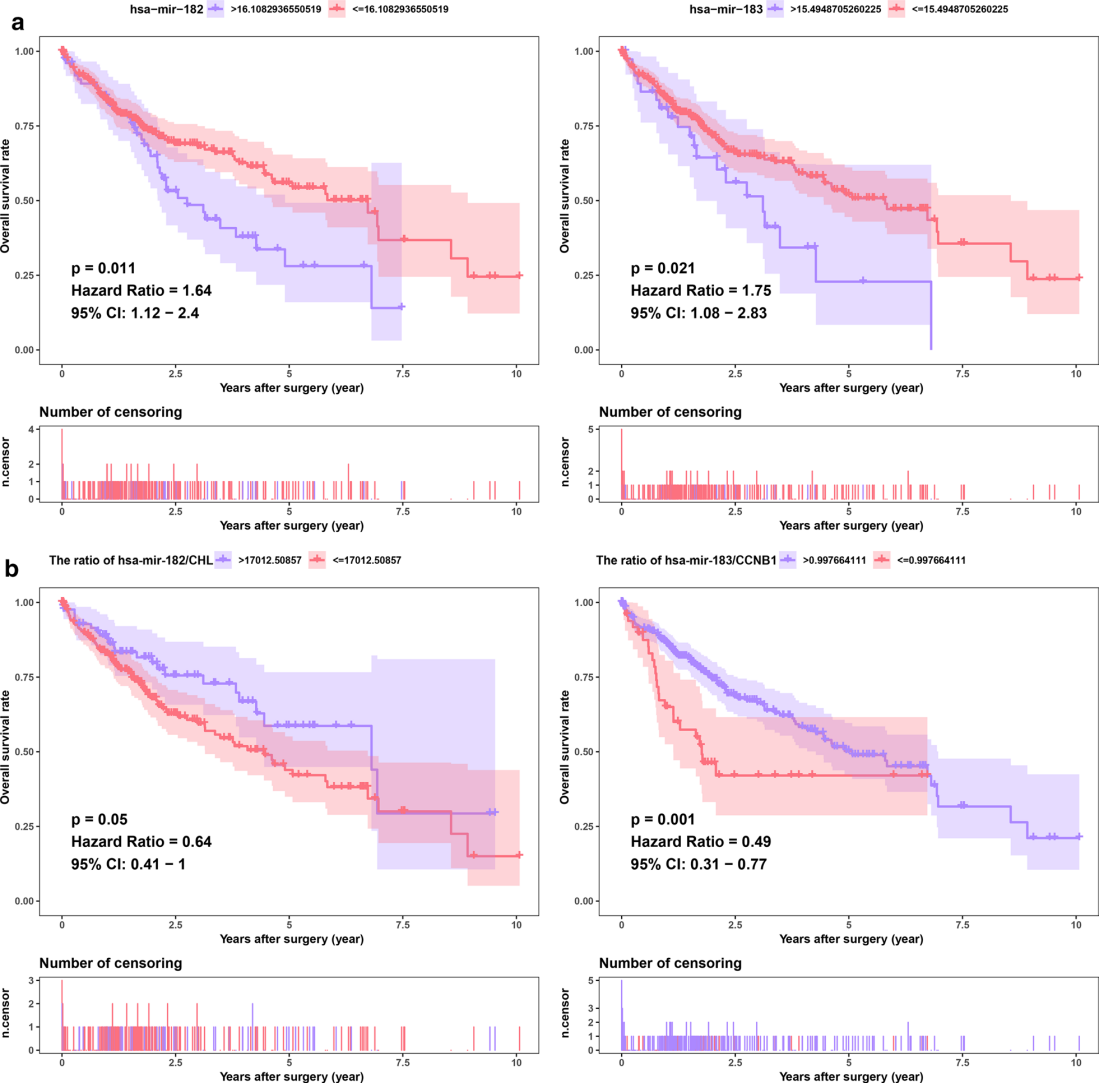
Survival analysis for DEmiRNAs. Kaplan–Meier survival curves for DEmiRNAs (**a**) and the ratios of DEmiRNAs to their target DEmRNAs (**b**) in the TCGA HCC cohorts. The horizontal axis indicates the overall survival time in years, and the vertical axis indicates the survival rate

To determine which DElncRNAs, DEmiRNAs and DEmRNAs have an impact on the overall survival of patients with HCC, survival analyses were conducted to investigate the Kaplan–Meier curves for HCC patients with the 26 DElncRNAs, four DEmiRNAs, and six DEmRNAs in both the TCGA and meta-GEO HCC cohorts. In the TCGA HCC cohort, 18 DElncRNAs and 73 DEmRNAs exhibited an obvious relationship to prognosis based on their respective optimal cutoffs (P < 0.05) (Additional file 6: Table S6, Additional file 7: Table S7). Meanwhile, in the meta-GEO HCC cohort, seven DElncRNAs and five DEmRNAs were significantly relevant to prognosis based on their respective optimal cutoffs (P < 0.05) (Additional file 8: Table S8, Additional file 9: Table S9). We consider that the genes with |log_2_ FC| > 2 in the TCGA HCC cohort and HR > 1 in both the TCGA and meta-GEO HCC cohorts were protective genes. In contrast, the genes with |log_2_ FC| < 2 in the TCGA HCC cohort and with HR < 1 in both the TCGA and meta-GEO HCC cohorts were conferred a risk of poor prognosis (“risky genes”). As a result, four DElncRNAs (MYCNOS, DLX6-AS1, LINC00221, and CRNDE) and two DEmRNAs (CCNB1 and SHCBP1) were identified as risky genes in both the TCGA and GEO meta-GEO HCC cohorts (Figs. 3, 4). However, due to the lack of miRNAs in the GEO database, we could not validate the miRNAs in another HCC cohort. Based on the above screening criteria, two DEmiRNAs (hsa-miR-182 and hsa-miR-183) were identified as risky genes in the TCGA HCC cohort (P < 0.05) (Fig. 5a) (Additional file 10: Table S10). In addition, we investigated whether the ratio of hsa-miR-182 with targeted RNAs (THBS1 and CHL1) and the ratio of hsa-miR-183 with targeted RNAs (CCNB1) have an impact on the prognosis of patients with HCC. We found that the ratio of hsa-miR-182/CHL1 and the ratio of hsa-miR-183/CCNB1 were significantly associated with the overall survival of patients with HCC (P < 0.05) (Fig. 5b).

### Validation of gene expression of DEmRNAs

The two DEmRNAs (CCNB1 and SHCBP1) related to prognosis were chosen for validation. The expression of CCNB1 and SHCBP1 were significantly higher in the HCC tissues than in the paired nontumorous liver tissues in the GSE64041 and GSE14520 HCC cohorts, which is consistent with our results in the TCGA HCC cohort (Fig. 4c–e). The results proved the reliability of our analysis.

### Gene set enrichment analysis

To identify the biological pathways associated with CCNB1 and SHCBP1, which were highly expressed in HCC, we conducted GSEA of the HCC samples based on the TCGA HCC cohort. HCC samples in the CCNB1 high-expression group were most significantly enriched for “sister chromatid segregation” (Fig. 4e) (Additional file 11: Table S11); HCC samples in the SHCBP1 high-expression group were most significantly enriched for “regulation of nuclear division” (Fig. 4f) (Additional file 12: Table S12).

## Discussion

Approximately 782,500 new cases of HCC occur each year, making HCC the second greatest cause of cancer mortality worldwide [24]. Although many treatment strategies (i.e., radiofrequency ablation, surgical resection, and liver transplantation) have been adopted, the prognoses of HCC patients who undergo these treatments are still not satisfactory [25]. Most patients are diagnosed at advanced stages due to the asymptomatic nature of the disease. Elucidating the molecular mechanisms and processes underlying HCC is of great importance for identifying new therapeutic targets and improving the clinical outcomes of patients with this disease. More and more studies indicate that lncRNAs play a vital role in biological functions through multiple levels of regulation, which involve transcriptional, posttranscriptional, and epigenetic regulation [26, 27]. A great deal of research has shown that there is a complex and closely related regulatory network between miRNAs and lncRNAs. In addition, lncRNAs and miRNAs have crucial functions in the pathogenesis and progression of cancers [27, 28]. Thus, these lncRNAs may serve as tumor-related prognostic indicators. Recently, Robinson and Henderson constructed a network model to identify hypothetical mRNA–miRNA interaction networks associated with epithelial function [29]. A mRNA–miRNA interaction list was generated using functional-molecular databases and network modeling. The authors quantified and visualized inherent network structures by using R code and identified a subnetwork containing a large number of shared, targeting miRNAs, of genes related to cancer and cellular proliferation, including cyclin D and c-MYC. The complexity of the miRNA–mRNA interaction network represents an obstacle for predicting and verifying the function of the ceRNA network [29]. The ceRNA hypothesis was proposed to explain the mechanism of tumorigenesis; this hypothesis provides a novel guiding theory and suggests valuable strategies and research directions for the diagnosis and treatment of malignancies [10]. lncRNAs with sequences similar to those of their target miRNAs are able to regulate the expression of mRNAs by acting as sponges for miRNAs [10].

To the best of our knowledge, few studies on ceRNAs have focused on predicting HCC prognosis. Additionally, rare yet reliable lncRNAs, miRNAs, and mRNAs related to HCC can be treated as molecular biomarkers to detect HCC and stratify the HCC risk. Under this background and hypothesis, lncRNA, mRNA, and miRNA data from 371 HCC samples were collected from the TCGA. Based on comprehensive integration of the lncRNA, mRNA, and miRNA data from the 43 paired HCC samples, a ceRNA network associated with lncRNAs was constructed to study the regulatory mechanism of the ceRNAs. Although the biogenesis of HCC is extremely complex, the complexity of tumor growth and metastasis dissemination can be largely represented by DEGs; therefore, we used DEGs to construct the ceRNA network. Here, we develop a ceRNA network including 26 lncRNAs, four miRNAs, and six mRNAs that are specific to HCC. Among them, four lncRNAs (MYCNOS, DLX6-AS1, LINC00221, and CRNDE) and two mRNAs (CCNB1 and SHCBP1) presented an obvious relevance to overall survival using patient data obtained from both the TCGA and GEO databases.

In the ceRNA network, the lncRNA LINC00221 (connection degree = 2) had the highest connection degree among the prognostic DElncRNAs (MYCNOS, DLX6-AS1, LINC00221, and CRNDE) (Additional file 5: Table S5). Therefore, we concluded that it might exert a strong influence on HCC pathogenesis. Russell et al. showed that the expression of LINC00221 is 2.4-fold higher in high-risk neuroblastomas than in low-risk neuroblastomas [30]. LINC00221 was involved in ceRNA networks in several tumor types. Fan et al. reported that LINC00221 is upregulated in breast cancer compared to adjacent-normal breast tissues and is involved in the lncRNA–miRNA–mRNA ceRNA network of breast cancer [31]. He et al. demonstrated that LINC00221 is dysregulated in gastric cancer and competes with six miRNAs (hsa‑miR‑96, hsa‑miR‑143, hsa‑miR‑204, hsa‑miR‑372, hsa‑miR‑373 and hsa‑miR‑519d) to mediate target mRNA expression in gastric cancer [32]. Zhang et al. found that LINC00221 is associated with a poor prognosis in patients with gastric cancer, and an 11‑lncRNA signature consisting of LINC00221 can predict the survival rate for gastric cancer [33]. Wang et al. noted that LINC00221 is involved in the lncRNA–miRNA–mRNA ceRNA network of muscle-invasive bladder cancer, and its high expression level was significantly related to the progression of muscle-invasive bladder cancer [34]. Our study also showed that LINC00221 expression is 77-fold higher in HCC tissues than in paired nontumorous tissues. Increased expression of LINC00221 was associated with a poor prognosis in patients with HCC. Moreover, we found that LINC00221 might compete with two key DEmiRNAs (hsa-miR-182 and hsa-miR-96) to mediate target DEmRNA expression in HCC.

In addition to lncRNAs, miRNAs should also receive extensive attention. Undoubtedly, research related to tumorigenicity in terms of miRNA regulation is critically needed. miRNAs are RNA molecules of approximately 22 nucleotides in length that bind to the 3′-untranslated region (3′-UTR) of their respective target genes and exert their influence on gene expression by either inhibiting protein translation or degrading mRNA [35]. miRNAs are known to be an integral component of cancer development [35]. We noted that the DEmiRNA hsa-miR-182 (connection degree = 13) had the highest connection degree among the prognostic DEmiRNAs (hsa-miR-182 and hsa-miR-183) in the ceRNA network, suggesting an obvious influence of hsa-miR-424 on HCC pathogenesis and prognosis (Additional file 5: Table S5). miR-182 is located on human chromosome 7q31-34 [36]. miR-182 promotes tumorigenesis in a variety of tumors and is one of the most frequently studied cancer-associated miRNAs [36]. In glioblastoma, miR-182-5p targets protein phosphatase 1 regulatory inhibitor subunit 1C [37]. By downregulating RAB27A expression, miR-182-5p improves the migration, mitosis, viability, and invasion capabilities of human gastric cancer cells [37]. Inhibiting miR-182-5p by regulating CASP9 expression confers pro-apoptotic and anti-proliferative effects in human breast cancer [38]. Activated STAT3 induces miR-182-5p expression, which enhances the growth of gliomas [39]. Tang et al. used real-time quantitative PCR to detect the expression of miR-182 in normal cervical epithelial cells and primary cervical cancer tissues and found that miR-182 was significantly upregulated in primary cervical cancer and that the expression level of miR-182 was significantly associated with the patient’s International Federation of Gynecology and Obstetrics (FIGO) cancer stage level [40]. Aberrant expression of miR-182 promotes melanoma metastasis by inhibiting microphthalmia-associated transcription factor and FOXO3 [41, 42]. In HCC, Yu et al. reported that miR-182 was one of the most significantly overexpressed miRNAs in HCC [43]. Moreover, the upregulation of miR-182 expression was associated with unfavorable prognosis of HCC patients and intrahepatic metastasis. In cisplatin-treated HCC cells, upregulated miR-182-5p promotes drug resistance by targeting tumor protein 53-induced nuclear protein 1 (TP53INP1) [42]. By regulating metastasis suppressor 1 (MTSS1), miR-182-5p contributes to HCC metastasis [43]. Furthermore, upregulated expression of miR-182-5p may be a diagnostic and prognostic indicator for HCC patients [41]. Consistent with previous studies, our study showed that miR-182 is upregulated by sixfold in HCC, is associated with poor prognosis, and may act through downstream targets (Additional file 1: Table S1).

In addition, we investigated whether the ratios of hsa-miR-182 to its target RNAs (THBS1 and CHL1) and the ratio of hsa-miR-183 to its target RNA (CCNB1) have an impact on the prognosis of patients with HCC. We found that the hsa-miR-182/CHL1 ratio and the hsa-miR-183/CCNB1 ratio were significantly associated with the overall survival of patients with HCC. Zhu et al. reported that upregulation of miR-182 was significantly associated with CHL1 downregulation in papillary thyroid carcinoma (PTC) cell lines and human PTC tissues [44]. miR-182 inhibits the expression of CHL1 by directly targeting the 3’-UTR. Downregulation of miR-182 inhibited the invasion and growth of PTC cells. They concluded that miR-182 in PTC promotes cell invasion and proliferation by directly inhibiting CHL1. In glioblastoma, anti-miR-182 expression results in increased expression of biomarkers for proliferation and stem cell biomarkers (e.g., CCNB1, CD44, Sox2, and Nestin) [45]. Xu et al. reported that hsa-miR-183/CCNB1 may be related to the effect of ribavirin on HCC [46]. Therefore, we believe that the hsa-miR-182/CHL1 and hsa-miR-183/CCNB1 axes may have a strong influence on HCC pathogenesis and may provide novel therapeutic targets for HCC treatment.

Among the prognostic DEmRNAs, CCNB1 and SHCBP1 had the same connection degree (connection degree = 1) in the ceRNA network (Additional file 5: Table S5). CCNB1, a rigorous quality control regulator and an important initiator of mitosis, is a key member of the cyclin family [47, 48]. By promoting the transition of the cell cycle from the G2 phase to mitosis, CCNB1 plays a key role in forming cyclin‐dependent kinase 1 (CDK1) complexes [47, 48]. The dysregulated expression of CCNB1 is observed in many different cancers, including melanoma and esophageal squamous cell carcinoma [49, 50]. There is increasing evidence that CCNB1 is involved in checkpoint control and that its dysfunction is an early event in tumorigenesis [49, 50]. At the same time, there is evidence that inhibition of CCNB1 expression makes breast cancer cells more sensitive to the chemotherapy drug paclitaxel [51]. CCNB1 also has significant predictive power in monitoring hormone therapy efficacy and the prognosis of patients with ER+ breast cancer [52]. CCNB1 is also a biomarker for HBV-related HCC recurrence [53]. The overexpression of CCNB1 was an independent factor for unfavorable disease-free survival and had an unfavorable prognosis in patients with lung adenocarcinoma [54]. Gu et al. found that CCNB1 overexpression is closely related to poor survival in patients with HCC [55]. Knockdown of CCNB1 by RNA interference significantly suppressed HCC cell invasion, migration, and proliferation. Furthermore, they also found that miR-144 inhibits CCNB1 expression by directly targeting CCNB1. Moreover, miR-144 negatively regulates CCNB1 to delay tumor formation. The study concluded that the miR-144/CCNB1 axis plays a vital role in HCC. In the present study, our results showed that CCNB1 was upregulated in HCC samples compared to the paired adjacent nontumorous samples from both the TCGA and GEO databases. Furthermore, the survival analysis indicated that patients with HCC exhibiting high CCNB1 expression levels presented a poor prognosis in both the TCGA and GEO databases.

SHC SH2-domain binding protein 1 (SHCBP1) is a cytoplasmic protein that couples signaling pathways to activated growth factor receptors [56]. The expression of SHCBP1 mRNA levels has significant individual differences in human normal tissues. SHCBP1 mRNA and protein are absent in normal, quiescent tissues but are selectively expressed in tissues containing proliferating cells or even cancer cells, demonstrating that SHCBP1 may be involved in tumor development [56]. Immunohistochemical analysis revealed that SHCBP1 was upregulated in breast cancer [57]. High expression level of SHCBP1 was related to poorer survival and advanced clinical stage [57]. In gliomas, by activating the NF-κB signaling pathway, increased expression of SHCBP1 contributed to invasion and migration [58]. In HCC, SHCBP1 was significantly overregulated [59]. Upregulation of SHCBP1 significantly promoted colony formation and survival and cell proliferation in HCC cell lines [59]. In parallel, knockdown of SHCBP1 suppressed cell proliferation and induced cell cycle delay [59]. Our findings indicate that SHCBP1 was upregulated in HCC and that its overexpression was related to poor prognosis in patients with HCC.

Although our ceRNA network identifies many HCC-related lncRNAs, miRNAs, and mRNAs, the correlation and the extent of ceRNA effects in vivo remain poorly understood. Recent experimental studies have shown that miRNA-mediated competition between ceRNAs plays a vital role in many biological contexts by constituting additional levels of posttranscriptional regulation [60]. Sensitivity analysis demonstrates that repression mechanisms and binding free energy are vital factors for cross-talk between ceRNAs. Interactions that occur within a particular range of inhibitory values can be asymmetric (one ceRNA influences another but not the reverse) or symmetrical (one ceRNA influences another and vice versa) and can be limited by noise; at the same time, the interactions can be highly selective [60]. All in all, there are many criteria for validating ceRNA networks, such as cellular concentrations of RNA-binding proteins (RBPs) and miRNAs, timescales, steady-state parameters, kinetic parameters, the absolute concentration of the effective target pool, the miRNA:target ratio, miRNA concentration, the size and affinities of the competing target pool, quantitative measurements of miRNA, target abundance, and so on [61–65]. Therefore, our results still need to be verified through in vivo and in vitro experiments and clinical practice. Although the ceRNA network has many interference factors in experimental verification, this deficit did not hinder the reliability of the ceRNA network because our network was based on rigorous processes. First, we included only cancer-specific lncRNAs, miRNAs, and mRNAs that had an absolute fold change > 4 and an adjusted P-value < 0.01. Second, the interactions between DElncRNAs and DEmiRNAs and between DEmiRNAs and DEmRNAs were predicted by experiment-supported databases, such as miRTarBase. These two approaches guarantee that the interactions identified not only occur in silico but are also based on experimentally supported evidence. Therefore, we believe that the genes in the current ceRNA network are important for HCC. In the future, with the emergence of larger sample sizes, better databases, and better algorithms, a more comprehensive ceRNA network will be constructed. In addition, further research is warranted on the functions of key ceRNAs in vivo and in vitro.

## Conclusions

In conclusion, a ceRNA network including 26 DElncRNAs, four DEmiRNAs, and six DEmRNAs was successfully built. Importantly, four lncRNAs (MYCNOS, DLX6-AS1, LINC00221, and CRNDE) and two mRNAs (CCNB1 and SHCBP1) were remarkably related to the prognosis of patients with HCC in both the TCGA and GEO databases. Our research provides novel insights that will increase our understanding of the prognosis-related ceRNA network in HCC. Furthermore, the candidate lncRNAs, miRNAs, and mRNAs involved in the ceRNA network can be further evaluated as potential therapeutic targets and prognostic biomarkers for HCC.

## Additional files

**Additional file 1: Table S1.** Differentially expressed genes between HCC samples and paired nontumorous samples.

**Additional file 2: Table S2.** Forty-four DElncRNAs interacted with nine DEmiRNAs retrieved from the miRcode database.

**Additional file 3: Table S3.** Four DEmiRNAs interacted with six DEmRNAs retrieved from the miRDB, miRTarBase and TargetScan databases.

**Additional file 4: Table S4.** Interactions of the ceRNA network in HCC.

**Additional file 5: Table S5.** The connection degree of each gene in the ceRNA network.

**Additional file 6: Table S6.** Eighteen DElncRNAs were associated with the overall survival of patients with HCC in the TCGA HCC cohort.

**Additional file 7: Table S7.** Six DEmRNAs were associated with the overall survival of patients with HCC in the TCGA HCC cohort.

**Additional file 8: Table S8.** Seven DElncRNAs were associated with the overall survival of patients with HCC in the meta-GEO HCC cohort.

**Additional file 9: Table S9.** Five DEmRNAs were associated with the overall survival of patients with HCC in the meta-GEO HCC cohort.

**Additional file 10: Table S10.** Three DEmiRNAs were associated with the overall survival of patients with HCC in the TCGA HCC cohort.

**Additional file 11: Table S11.** Gene enrichment in the high CCNB1 expression group of patients with HCC in the TCGA HCC cohort.

**Additional file 12: Table S12.** Gene enrichment in the high SHCBP1 expression group of patients with HCC in the TCGA HCC cohort.

**Additional file 13.** R code.

## Abbreviations

HCC: hepatocellular carcinoma
ceRNA: competing endogenous RNA
lncRNA: long noncoding RNA
miRNA: microRNA
DEG: differentially expressed gene
DElncRNA: differentially expressed lncRNA
DEmiRNA: differentially expressed miRNA
DEmRNA: differentially expressed mRNA
TCGA: The Cancer Genome Atlas
GEO: Gene Expression Omnibus
FC: fold change
GSEA: gene set enrichment analysis
HR: hazard ratio
MRE: miRNA response element
TMM: trimmed mean of M value
FDR: false discovery rate
