# Supplementary material for: Construction and comprehensive analysis of a ceRNA network to reveal potential prognostic biomarkers for hepatocellular carcinoma
Source: Cancer Cell Int. 2019 Apr 11;19:90. doi: 10.1186/s12935-019-0817-y (PMC6458652; doi:10.1186/s12935-019-0817-y)
Supplement: Supplementary file 1 — Additional file 1: Table S1. Differentially expressed genes between HCC samples and paired nontumorous samples. [file 12935_2019_817_MOESM1_ESM.docx]

**Table S1. Differentially expressed genes between HCC samples and paired nontumorous samples.**

| **Gene** | **Type** | **Log_2_-fold change** | **P-value** | **Adjusted P-value** |
| --- | --- | --- | --- | --- |
| AC107396.1 | lncRNA | -6.489919517 | 4.62E-24 | 2.92E-20 |
| LINC01419 | lncRNA | 9.551752907 | 1.11E-23 | 3.50E-20 |
| AC004080.2 | lncRNA | 5.892519645 | 4.00E-23 | 8.43E-20 |
| AC010776.2 | lncRNA | -5.337363335 | 2.65E-22 | 4.19E-19 |
| SFTA1P | lncRNA | 5.31034979 | 5.95E-22 | 7.52E-19 |
| LINC02163 | lncRNA | 6.487586085 | 7.25E-21 | 7.65E-18 |
| LINC02476 | lncRNA | 7.706901246 | 2.48E-20 | 2.24E-17 |
| LINC02241 | lncRNA | 8.450531052 | 1.26E-19 | 9.99E-17 |
| LINC02475 | lncRNA | 6.31953907 | 3.28E-18 | 2.31E-15 |
| MAFA-AS1 | lncRNA | 5.479637771 | 8.51E-18 | 5.20E-15 |
| LINC00355 | lncRNA | 6.699402533 | 9.15E-18 | 5.20E-15 |
| LINC01194 | lncRNA | 7.038109688 | 1.00E-17 | 5.20E-15 |
| HOTTIP | lncRNA | 6.603634014 | 1.07E-17 | 5.20E-15 |
| AC104809.2 | lncRNA | -4.129349854 | 1.38E-17 | 6.22E-15 |
| PCA3 | lncRNA | -4.489201143 | 1.75E-17 | 7.37E-15 |
| LINC01612 | lncRNA | -6.21932393 | 2.27E-17 | 8.97E-15 |
| AC091987.1 | lncRNA | 5.709260113 | 2.67E-17 | 9.92E-15 |
| AC245100.6 | lncRNA | 7.041876575 | 3.25E-17 | 1.14E-14 |
| FENDRR | lncRNA | -4.183805149 | 6.42E-17 | 2.14E-14 |
| AC093725.2 | lncRNA | -4.610840601 | 7.89E-17 | 2.50E-14 |
| AL391845.2 | lncRNA | 4.074244957 | 8.73E-17 | 2.63E-14 |
| HAGLROS | lncRNA | 4.460127374 | 1.46E-16 | 4.19E-14 |
| AC104809.1 | lncRNA | -7.529322811 | 1.64E-16 | 4.51E-14 |
| AC245128.3 | lncRNA | -3.345859389 | 2.00E-16 | 5.08E-14 |
| HAGLR | lncRNA | 4.408988652 | 2.01E-16 | 5.08E-14 |
| AC024230.1 | lncRNA | 5.528644366 | 2.44E-16 | 5.94E-14 |
| LINC01093 | lncRNA | -6.352185198 | 2.54E-16 | 5.95E-14 |
| AC008556.1 | lncRNA | -2.392901409 | 3.88E-16 | 8.78E-14 |
| AL136537.2 | lncRNA | 6.078041827 | 4.13E-16 | 9.01E-14 |
| LINC02377 | lncRNA | 6.04627984 | 6.56E-16 | 1.38E-13 |
| LINP1 | lncRNA | -4.390278068 | 8.12E-16 | 1.66E-13 |
| AC092171.2 | lncRNA | 2.546873738 | 9.23E-16 | 1.82E-13 |
| AF165147.1 | lncRNA | -3.567030352 | 1.54E-15 | 2.95E-13 |
| LINC01532 | lncRNA | 5.773454188 | 3.41E-15 | 6.34E-13 |
| B3GALT5-AS1 | lncRNA | -4.681591482 | 3.71E-15 | 6.71E-13 |
| PCDH9-AS2 | lncRNA | -5.159089105 | 4.28E-15 | 7.51E-13 |
| AL136987.1 | lncRNA | 4.695313852 | 4.41E-15 | 7.53E-13 |
| HAND2-AS1 | lncRNA | -4.738053132 | 6.83E-15 | 1.14E-12 |
| LINC02335 | lncRNA | 5.635612442 | 1.49E-14 | 2.42E-12 |
| AC090809.1 | lncRNA | 6.457733541 | 1.80E-14 | 2.85E-12 |
| AC116049.2 | lncRNA | 4.732458488 | 2.11E-14 | 3.25E-12 |
| AC010547.2 | lncRNA | -5.442790772 | 2.33E-14 | 3.46E-12 |
| AC129507.1 | lncRNA | -2.291618276 | 2.38E-14 | 3.46E-12 |
| DDX11-AS1 | lncRNA | 2.497502313 | 2.41E-14 | 3.46E-12 |
| AC016550.2 | lncRNA | 5.554210935 | 2.49E-14 | 3.49E-12 |
| AC087477.4 | lncRNA | -4.171868744 | 2.73E-14 | 3.73E-12 |
| HHIP-AS1 | lncRNA | -3.767584499 | 2.77E-14 | 3.73E-12 |
| LINC00906 | lncRNA | 5.296579734 | 2.91E-14 | 3.84E-12 |
| AC092384.2 | lncRNA | -4.720767434 | 3.36E-14 | 4.34E-12 |
| AC138356.1 | lncRNA | -2.923829857 | 4.07E-14 | 5.14E-12 |
| AL157778.1 | lncRNA | 5.64671399 | 4.23E-14 | 5.25E-12 |
| AC239868.2 | lncRNA | 2.107822258 | 6.16E-14 | 7.49E-12 |
| LINC01727 | lncRNA | -3.964120118 | 7.46E-14 | 8.91E-12 |
| HOXA11-AS | lncRNA | 5.094764613 | 7.93E-14 | 9.29E-12 |
| AC004540.2 | lncRNA | -4.49553594 | 1.27E-13 | 1.46E-11 |
| AC004477.1 | lncRNA | 2.417501359 | 1.29E-13 | 1.46E-11 |
| DUXAP8 | lncRNA | 2.999219768 | 1.94E-13 | 2.13E-11 |
| AC010595.1 | lncRNA | 5.138123489 | 1.96E-13 | 2.13E-11 |
| AL606489.1 | lncRNA | 3.421322328 | 2.02E-13 | 2.13E-11 |
| AC010280.2 | lncRNA | -4.06007677 | 2.04E-13 | 2.13E-11 |
| AC092957.1 | lncRNA | 5.22917292 | 2.05E-13 | 2.13E-11 |
| AC026369.3 | lncRNA | -3.703304842 | 2.44E-13 | 2.49E-11 |
| AC112178.1 | lncRNA | 5.305478339 | 3.54E-13 | 3.55E-11 |
| CDKN2B-AS1 | lncRNA | 3.236726926 | 4.39E-13 | 4.34E-11 |
| AC105118.1 | lncRNA | 5.152818785 | 5.04E-13 | 4.90E-11 |
| LINC01224 | lncRNA | 4.898595663 | 5.40E-13 | 5.18E-11 |
| AC090502.1 | lncRNA | 7.271985977 | 5.54E-13 | 5.23E-11 |
| AC110995.1 | lncRNA | -2.020105643 | 6.56E-13 | 6.10E-11 |
| AC087392.1 | lncRNA | -4.439631107 | 7.68E-13 | 7.04E-11 |
| AC010719.1 | lncRNA | 2.562074746 | 9.83E-13 | 8.76E-11 |
| AC004160.1 | lncRNA | -3.931841926 | 1.17E-12 | 1.03E-10 |
| LINC02027 | lncRNA | -5.98898286 | 1.36E-12 | 1.18E-10 |
| AL023803.2 | lncRNA | 3.191403409 | 1.39E-12 | 1.19E-10 |
| AL035401.1 | lncRNA | 4.84615785 | 2.37E-12 | 1.98E-10 |
| AL136084.3 | lncRNA | 3.83488379 | 2.45E-12 | 2.02E-10 |
| FAM99A | lncRNA | -6.434879028 | 2.72E-12 | 2.20E-10 |
| LINC01235 | lncRNA | 3.631884826 | 3.22E-12 | 2.58E-10 |
| AC012409.1 | lncRNA | -2.567615559 | 3.35E-12 | 2.65E-10 |
| AP006285.2 | lncRNA | -4.020612655 | 4.31E-12 | 3.37E-10 |
| AC068858.1 | lncRNA | 3.504171847 | 4.48E-12 | 3.46E-10 |
| LINC01833 | lncRNA | 6.798918087 | 4.58E-12 | 3.49E-10 |
| FAM99B | lncRNA | -4.966663075 | 4.84E-12 | 3.65E-10 |
| LINC01767 | lncRNA | -2.682941692 | 5.24E-12 | 3.90E-10 |
| AC092155.1 | lncRNA | -3.319482892 | 5.48E-12 | 4.00E-10 |
| AC084864.1 | lncRNA | 3.951397528 | 5.50E-12 | 4.00E-10 |
| SMIM25 | lncRNA | -2.268140155 | 5.75E-12 | 4.13E-10 |
| LINC01704 | lncRNA | 3.480935032 | 6.26E-12 | 4.45E-10 |
| LINC01287 | lncRNA | 8.2707612 | 6.66E-12 | 4.68E-10 |
| LINC02580 | lncRNA | -2.132826248 | 7.37E-12 | 5.12E-10 |
| C17orf82 | lncRNA | 2.585036288 | 7.54E-12 | 5.18E-10 |
| LINC00491 | lncRNA | 4.630761561 | 7.67E-12 | 5.22E-10 |
| AL117329.1 | lncRNA | 5.002856614 | 8.18E-12 | 5.51E-10 |
| AC006960.2 | lncRNA | -4.651605996 | 8.60E-12 | 5.72E-10 |
| LINC00885 | lncRNA | -4.604885599 | 9.35E-12 | 6.10E-10 |
| AC114316.2 | lncRNA | 3.891701826 | 1.01E-11 | 6.54E-10 |
| LINC02202 | lncRNA | 2.698782246 | 1.06E-11 | 6.78E-10 |
| KCNMB2-AS1 | lncRNA | 3.852165253 | 1.12E-11 | 7.11E-10 |
| LINC01863 | lncRNA | -3.413676679 | 1.27E-11 | 7.93E-10 |
| LEF1-AS1 | lncRNA | 3.646815635 | 1.28E-11 | 7.95E-10 |
| AC106771.1 | lncRNA | 4.79759849 | 1.30E-11 | 8.01E-10 |
| AC004160.2 | lncRNA | -3.529390647 | 1.40E-11 | 8.53E-10 |
| LY6E-DT | lncRNA | -3.775465208 | 1.50E-11 | 9.02E-10 |
| AC061975.7 | lncRNA | -4.258743112 | 1.57E-11 | 9.35E-10 |
| AC107419.1 | lncRNA | 4.44873579 | 1.93E-11 | 1.13E-09 |
| AC109830.1 | lncRNA | 4.73136974 | 2.02E-11 | 1.17E-09 |
| AC079061.1 | lncRNA | -3.267954388 | 2.35E-11 | 1.35E-09 |
| DNM3OS | lncRNA | -2.40519748 | 2.47E-11 | 1.41E-09 |
| AC000067.1 | lncRNA | 3.246806728 | 2.61E-11 | 1.47E-09 |
| AC016877.3 | lncRNA | 3.747490042 | 3.03E-11 | 1.69E-09 |
| AC026461.2 | lncRNA | -3.660895741 | 3.06E-11 | 1.70E-09 |
| AC109588.1 | lncRNA | 6.044625109 | 3.50E-11 | 1.91E-09 |
| HTR2A-AS1 | lncRNA | -2.870679817 | 3.69E-11 | 1.99E-09 |
| AP000439.3 | lncRNA | -3.188712822 | 3.73E-11 | 1.99E-09 |
| AL133467.4 | lncRNA | 5.03027154 | 3.74E-11 | 1.99E-09 |
| LINC01370 | lncRNA | -5.394773605 | 4.76E-11 | 2.51E-09 |
| LINC01667 | lncRNA | 6.491603394 | 4.94E-11 | 2.58E-09 |
| AC099508.2 | lncRNA | -3.885918926 | 5.27E-11 | 2.71E-09 |
| LINC01818 | lncRNA | -3.227269312 | 5.69E-11 | 2.90E-09 |
| AC019257.1 | lncRNA | -3.310797095 | 5.80E-11 | 2.94E-09 |
| LINC01108 | lncRNA | 3.45753808 | 5.96E-11 | 2.99E-09 |
| AC079789.1 | lncRNA | -3.413839715 | 6.91E-11 | 3.41E-09 |
| AL138900.2 | lncRNA | 5.145424485 | 6.96E-11 | 3.41E-09 |
| CDKN2A-AS1 | lncRNA | 3.68551139 | 7.37E-11 | 3.56E-09 |
| UCA1 | lncRNA | -3.641461589 | 7.44E-11 | 3.57E-09 |
| NCOA7-AS1 | lncRNA | -3.093370335 | 8.23E-11 | 3.91E-09 |
| AL021328.1 | lncRNA | -4.68759567 | 8.33E-11 | 3.93E-09 |
| MIR548XHG | lncRNA | 5.852020865 | 8.51E-11 | 3.99E-09 |
| LINC00683 | lncRNA | -3.442424863 | 8.75E-11 | 4.07E-09 |
| LINC00879 | lncRNA | 5.173555922 | 9.80E-11 | 4.49E-09 |
| AC015468.1 | lncRNA | -3.220786562 | 1.04E-10 | 4.72E-09 |
| AC090227.2 | lncRNA | -3.153935161 | 1.14E-10 | 5.15E-09 |
| AL162413.1 | lncRNA | 5.475468219 | 1.17E-10 | 5.22E-09 |
| AL451074.2 | lncRNA | 2.017072783 | 1.35E-10 | 5.96E-09 |
| HOXD-AS2 | lncRNA | 4.001804687 | 1.47E-10 | 6.45E-09 |
| AC093895.1 | lncRNA | 4.433575497 | 1.96E-10 | 8.42E-09 |
| HAO2-IT1 | lncRNA | -3.486670573 | 2.11E-10 | 9.02E-09 |
| WARS2-IT1 | lncRNA | -2.758123106 | 2.36E-10 | 9.96E-09 |
| LINC02388 | lncRNA | -2.893117425 | 2.74E-10 | 1.14E-08 |
| AC016999.1 | lncRNA | -2.810635553 | 2.92E-10 | 1.20E-08 |
| LINC00853 | lncRNA | 2.468931337 | 2.98E-10 | 1.20E-08 |
| AC011632.1 | lncRNA | 4.62656114 | 2.98E-10 | 1.20E-08 |
| LINC00704 | lncRNA | -2.932203377 | 3.03E-10 | 1.21E-08 |
| LINC01726 | lncRNA | -3.095430545 | 3.04E-10 | 1.21E-08 |
| AL133419.1 | lncRNA | -4.340532087 | 3.36E-10 | 1.31E-08 |
| NOVA1-AS1 | lncRNA | 4.674985012 | 3.37E-10 | 1.31E-08 |
| LINC01980 | lncRNA | 6.210700782 | 3.38E-10 | 1.31E-08 |
| AL392089.1 | lncRNA | -3.193997659 | 3.55E-10 | 1.36E-08 |
| AC016710.1 | lncRNA | 5.292855179 | 3.59E-10 | 1.37E-08 |
| LINC01561 | lncRNA | -3.351565767 | 3.80E-10 | 1.43E-08 |
| LINC02404 | lncRNA | 5.958831342 | 3.83E-10 | 1.44E-08 |
| AL590426.2 | lncRNA | -3.587841459 | 4.14E-10 | 1.54E-08 |
| AL161668.3 | lncRNA | -2.812958325 | 4.31E-10 | 1.58E-08 |
| AC090192.2 | lncRNA | 4.618181303 | 4.71E-10 | 1.71E-08 |
| AP002954.1 | lncRNA | -2.024458146 | 5.87E-10 | 2.09E-08 |
| AL139385.1 | lncRNA | -2.738008058 | 5.94E-10 | 2.10E-08 |
| LINC01507 | lncRNA | -4.268326568 | 5.98E-10 | 2.10E-08 |
| FEZF1-AS1 | lncRNA | 4.458444195 | 6.48E-10 | 2.27E-08 |
| AC131391.1 | lncRNA | 4.472899258 | 6.58E-10 | 2.29E-08 |
| LINC00907 | lncRNA | -3.480396879 | 7.57E-10 | 2.62E-08 |
| MAFG-AS1 | lncRNA | 2.267581434 | 7.63E-10 | 2.62E-08 |
| AC116025.2 | lncRNA | 2.994509767 | 8.14E-10 | 2.77E-08 |
| AC117386.2 | lncRNA | 4.21402732 | 8.29E-10 | 2.80E-08 |
| AL353708.3 | lncRNA | 2.010421146 | 8.41E-10 | 2.83E-08 |
| AC005150.1 | lncRNA | 5.516881795 | 9.37E-10 | 3.10E-08 |
| U91324.1 | lncRNA | -2.374265516 | 1.12E-09 | 3.63E-08 |
| LINC02428 | lncRNA | -3.859429711 | 1.12E-09 | 3.63E-08 |
| AL590666.2 | lncRNA | 3.06766952 | 1.23E-09 | 3.94E-08 |
| CASC20 | lncRNA | 5.116249849 | 1.25E-09 | 3.97E-08 |
| CRNDE | lncRNA | 2.554766778 | 1.45E-09 | 4.55E-08 |
| AL137060.1 | lncRNA | 2.243808356 | 1.46E-09 | 4.55E-08 |
| LINC01234 | lncRNA | 5.692578617 | 1.59E-09 | 4.92E-08 |
| LINC00707 | lncRNA | -3.353311959 | 1.72E-09 | 5.27E-08 |
| AL139042.1 | lncRNA | 4.361245562 | 1.85E-09 | 5.64E-08 |
| LINC02492 | lncRNA | 4.502636741 | 1.87E-09 | 5.67E-08 |
| BPESC1 | lncRNA | 3.495930603 | 1.94E-09 | 5.85E-08 |
| LINC02561 | lncRNA | 2.994039509 | 2.01E-09 | 5.99E-08 |
| AL133215.2 | lncRNA | 2.137532167 | 2.06E-09 | 6.13E-08 |
| AL731684.1 | lncRNA | 4.322176874 | 2.13E-09 | 6.30E-08 |
| AC020978.5 | lncRNA | -2.469552438 | 2.27E-09 | 6.66E-08 |
| AL118511.2 | lncRNA | 3.292132661 | 2.64E-09 | 7.66E-08 |
| AL121906.2 | lncRNA | 2.290998126 | 2.73E-09 | 7.87E-08 |
| AL139412.1 | lncRNA | 2.269060848 | 2.80E-09 | 8.03E-08 |
| AL078590.3 | lncRNA | -2.621848006 | 2.87E-09 | 8.11E-08 |
| FP325330.3 | lncRNA | 3.071462433 | 2.92E-09 | 8.18E-08 |
| AC099850.3 | lncRNA | 2.239678517 | 3.12E-09 | 8.65E-08 |
| LINC02525 | lncRNA | 4.881565916 | 3.15E-09 | 8.70E-08 |
| AL365181.3 | lncRNA | 3.431558611 | 3.19E-09 | 8.77E-08 |
| AC129507.2 | lncRNA | -2.033481839 | 3.30E-09 | 9.05E-08 |
| AC016395.1 | lncRNA | -2.405494868 | 3.52E-09 | 9.60E-08 |
| STAU2-AS1 | lncRNA | 2.746781621 | 3.65E-09 | 9.87E-08 |
| LINC01970 | lncRNA | 2.348448103 | 3.92E-09 | 1.05E-07 |
| AC132872.2 | lncRNA | 2.326717636 | 4.06E-09 | 1.08E-07 |
| H19 | lncRNA | -3.444872468 | 4.18E-09 | 1.11E-07 |
| AL358075.2 | lncRNA | 2.629171249 | 4.55E-09 | 1.20E-07 |
| LINC01775 | lncRNA | 2.929684618 | 4.71E-09 | 1.24E-07 |
| LINC01446 | lncRNA | 4.73115221 | 4.93E-09 | 1.29E-07 |
| AL359313.1 | lncRNA | 4.755830032 | 5.63E-09 | 1.45E-07 |
| AC062015.1 | lncRNA | 4.690437144 | 5.91E-09 | 1.51E-07 |
| CELSR3-AS1 | lncRNA | 2.360747268 | 6.10E-09 | 1.55E-07 |
| AL161740.1 | lncRNA | -2.002647164 | 6.37E-09 | 1.61E-07 |
| AC099684.1 | lncRNA | -2.641891941 | 6.47E-09 | 1.63E-07 |
| POU6F2-AS1 | lncRNA | -3.582455791 | 6.61E-09 | 1.65E-07 |
| LINC01348 | lncRNA | -3.415327202 | 6.62E-09 | 1.65E-07 |
| AC069277.1 | lncRNA | 4.277117172 | 6.63E-09 | 1.65E-07 |
| C3orf67-AS1 | lncRNA | 2.956979138 | 6.72E-09 | 1.67E-07 |
| AC108748.1 | lncRNA | -2.454259862 | 6.86E-09 | 1.69E-07 |
| LINC01630 | lncRNA | 4.353252614 | 7.00E-09 | 1.71E-07 |
| AC010969.1 | lncRNA | -3.590848604 | 7.01E-09 | 1.71E-07 |
| PCAT18 | lncRNA | -2.707870768 | 7.28E-09 | 1.77E-07 |
| SAMMSON | lncRNA | 3.702694674 | 9.19E-09 | 2.20E-07 |
| LINC01901 | lncRNA | 3.632291065 | 9.21E-09 | 2.20E-07 |
| LINC02267 | lncRNA | 4.208198553 | 9.25E-09 | 2.20E-07 |
| AL133467.2 | lncRNA | 4.573825871 | 9.47E-09 | 2.23E-07 |
| LINC01714 | lncRNA | -2.337265617 | 1.04E-08 | 2.43E-07 |
| PTGES2-AS1 | lncRNA | 2.75082125 | 1.11E-08 | 2.60E-07 |
| LINC00628 | lncRNA | 2.557798678 | 1.16E-08 | 2.69E-07 |
| AC114489.1 | lncRNA | 4.423732493 | 1.18E-08 | 2.73E-07 |
| MIR2052HG | lncRNA | 3.7217023 | 1.35E-08 | 3.09E-07 |
| NPSR1-AS1 | lncRNA | 4.283324511 | 1.36E-08 | 3.10E-07 |
| C14orf144 | lncRNA | -2.710279556 | 1.37E-08 | 3.10E-07 |
| LINC02153 | lncRNA | -2.880569274 | 1.40E-08 | 3.15E-07 |
| AL391095.2 | lncRNA | -2.112200875 | 1.41E-08 | 3.17E-07 |
| AFAP1-AS1 | lncRNA | 3.989034983 | 1.55E-08 | 3.47E-07 |
| AP003774.3 | lncRNA | -2.049722655 | 1.58E-08 | 3.51E-07 |
| AC106795.2 | lncRNA | -3.150173998 | 1.71E-08 | 3.79E-07 |
| LINC02159 | lncRNA | 3.385102707 | 1.76E-08 | 3.88E-07 |
| LINC00221 | lncRNA | 6.272487664 | 1.85E-08 | 4.06E-07 |
| AC007128.1 | lncRNA | 4.3974266 | 1.92E-08 | 4.19E-07 |
| AC024600.1 | lncRNA | -2.578575722 | 1.95E-08 | 4.23E-07 |
| AC022167.4 | lncRNA | -2.441436511 | 2.10E-08 | 4.56E-07 |
| AC008991.1 | lncRNA | 3.916066258 | 2.16E-08 | 4.63E-07 |
| AC010280.1 | lncRNA | -2.85218322 | 2.24E-08 | 4.78E-07 |
| FAM230C | lncRNA | 4.848226345 | 2.33E-08 | 4.97E-07 |
| AC008060.1 | lncRNA | 4.488330827 | 2.38E-08 | 5.03E-07 |
| AL445228.2 | lncRNA | 2.854912639 | 2.46E-08 | 5.19E-07 |
| AL645608.1 | lncRNA | -3.224767095 | 2.49E-08 | 5.23E-07 |
| LINC01842 | lncRNA | 3.121926801 | 2.55E-08 | 5.35E-07 |
| AP000526.1 | lncRNA | 3.223138043 | 2.61E-08 | 5.43E-07 |
| AL138749.1 | lncRNA | -4.153664714 | 2.91E-08 | 6.02E-07 |
| AL035661.1 | lncRNA | -3.336015739 | 3.09E-08 | 6.37E-07 |
| AC106822.1 | lncRNA | -2.263077813 | 3.22E-08 | 6.59E-07 |
| AC015468.4 | lncRNA | -2.755348782 | 3.29E-08 | 6.71E-07 |
| LINC02275 | lncRNA | -3.501468314 | 3.46E-08 | 7.01E-07 |
| AC011294.1 | lncRNA | 3.417161894 | 3.57E-08 | 7.17E-07 |
| LINC00383 | lncRNA | 4.038028118 | 3.62E-08 | 7.24E-07 |
| AC112206.2 | lncRNA | -3.494053421 | 3.69E-08 | 7.36E-07 |
| AP000525.1 | lncRNA | 2.819202651 | 3.84E-08 | 7.63E-07 |
| AP002478.1 | lncRNA | 3.819141673 | 3.97E-08 | 7.84E-07 |
| DLGAP1-AS3 | lncRNA | -3.15501794 | 4.15E-08 | 8.13E-07 |
| AP001043.1 | lncRNA | -2.457342639 | 4.27E-08 | 8.33E-07 |
| LINC01702 | lncRNA | -2.933915374 | 4.61E-08 | 8.97E-07 |
| AL139023.1 | lncRNA | 4.060608542 | 4.72E-08 | 9.11E-07 |
| AC097515.1 | lncRNA | 3.510207209 | 4.72E-08 | 9.11E-07 |
| AC091729.2 | lncRNA | -3.629743954 | 4.72E-08 | 9.11E-07 |
| LINC01019 | lncRNA | 4.857635973 | 4.74E-08 | 9.12E-07 |
| AP001178.3 | lncRNA | 2.815238983 | 5.02E-08 | 9.62E-07 |
| TMEM26-AS1 | lncRNA | -2.498993362 | 5.44E-08 | 1.03E-06 |
| AL139130.1 | lncRNA | 3.052662987 | 5.56E-08 | 1.05E-06 |
| AF279873.3 | lncRNA | 5.060959984 | 5.94E-08 | 1.11E-06 |
| LINC00399 | lncRNA | -2.462060216 | 5.98E-08 | 1.12E-06 |
| AP001065.1 | lncRNA | -2.013326581 | 6.19E-08 | 1.15E-06 |
| ATP2A1-AS1 | lncRNA | 2.248698744 | 6.61E-08 | 1.22E-06 |
| AC110285.6 | lncRNA | 2.365092951 | 6.71E-08 | 1.23E-06 |
| AL161630.1 | lncRNA | -2.918637226 | 7.52E-08 | 1.37E-06 |
| AC008080.1 | lncRNA | -2.297993445 | 8.09E-08 | 1.45E-06 |
| AF254983.1 | lncRNA | 3.756160595 | 8.36E-08 | 1.50E-06 |
| AC096577.1 | lncRNA | -4.172187372 | 8.51E-08 | 1.52E-06 |
| AL357060.1 | lncRNA | 3.400580344 | 8.64E-08 | 1.53E-06 |
| MAGI2-AS3 | lncRNA | -2.68562239 | 8.64E-08 | 1.53E-06 |
| LINC01136 | lncRNA | 2.229127547 | 8.80E-08 | 1.55E-06 |
| LINC01608 | lncRNA | 4.558936637 | 9.50E-08 | 1.67E-06 |
| LINC01748 | lncRNA | 3.577939183 | 1.05E-07 | 1.83E-06 |
| G2E3-AS1 | lncRNA | 4.236586885 | 1.15E-07 | 1.99E-06 |
| RASGRF2-AS1 | lncRNA | 2.566892167 | 1.18E-07 | 2.01E-06 |
| AL049555.1 | lncRNA | -2.947119044 | 1.19E-07 | 2.03E-06 |
| AL355974.2 | lncRNA | -4.327332369 | 1.21E-07 | 2.07E-06 |
| AC062004.1 | lncRNA | -2.619730516 | 1.23E-07 | 2.09E-06 |
| AC005858.1 | lncRNA | 3.836682165 | 1.25E-07 | 2.13E-06 |
| LINC01625 | lncRNA | -2.909106314 | 1.31E-07 | 2.21E-06 |
| MIR3945HG | lncRNA | -2.087604887 | 1.38E-07 | 2.31E-06 |
| AL390778.2 | lncRNA | -4.140929773 | 1.39E-07 | 2.33E-06 |
| AC134312.6 | lncRNA | -2.330620884 | 1.40E-07 | 2.33E-06 |
| RBMS3-AS3 | lncRNA | -2.13712346 | 1.40E-07 | 2.33E-06 |
| ST8SIA6-AS1 | lncRNA | 4.346631525 | 1.45E-07 | 2.40E-06 |
| AL365181.2 | lncRNA | 3.281613593 | 1.51E-07 | 2.49E-06 |
| AC074327.1 | lncRNA | 4.298992531 | 1.58E-07 | 2.60E-06 |
| FER1L6-AS2 | lncRNA | 3.464819002 | 1.58E-07 | 2.60E-06 |
| LINC01524 | lncRNA | 3.758812417 | 1.60E-07 | 2.62E-06 |
| FAM83A-AS1 | lncRNA | -3.80680559 | 1.63E-07 | 2.65E-06 |
| AC010175.1 | lncRNA | -2.029872215 | 1.72E-07 | 2.78E-06 |
| LINC00864 | lncRNA | -3.389458964 | 1.85E-07 | 2.97E-06 |
| AC018742.1 | lncRNA | -2.136750287 | 1.89E-07 | 3.01E-06 |
| AL591848.2 | lncRNA | 2.828277944 | 1.90E-07 | 3.02E-06 |
| CTD-3080P12.3 | lncRNA | -2.24767213 | 1.90E-07 | 3.02E-06 |
| AC010333.1 | lncRNA | 3.828121375 | 2.05E-07 | 3.24E-06 |
| AL449403.2 | lncRNA | -2.807272609 | 2.13E-07 | 3.34E-06 |
| AC006065.4 | lncRNA | 4.476636083 | 2.20E-07 | 3.42E-06 |
| AL355601.1 | lncRNA | 3.464981717 | 2.30E-07 | 3.54E-06 |
| AC090796.1 | lncRNA | -3.311323706 | 2.38E-07 | 3.63E-06 |
| AL080248.1 | lncRNA | -2.511045499 | 2.43E-07 | 3.68E-06 |
| FIRRE | lncRNA | 2.800502087 | 2.46E-07 | 3.71E-06 |
| AC005224.3 | lncRNA | -2.148064324 | 2.49E-07 | 3.74E-06 |
| LINC02156 | lncRNA | -2.762187777 | 2.56E-07 | 3.82E-06 |
| AGAP1-IT1 | lncRNA | -2.103127472 | 2.60E-07 | 3.86E-06 |
| AP003716.1 | lncRNA | -2.887114335 | 2.67E-07 | 3.95E-06 |
| AL512652.1 | lncRNA | 2.374362901 | 2.79E-07 | 4.11E-06 |
| AL731897.1 | lncRNA | 7.133205433 | 2.82E-07 | 4.13E-06 |
| MNX1-AS1 | lncRNA | 5.050477553 | 2.95E-07 | 4.30E-06 |
| AP003469.2 | lncRNA | 3.462772032 | 3.02E-07 | 4.38E-06 |
| AC015712.6 | lncRNA | -2.677653424 | 3.13E-07 | 4.51E-06 |
| AC106900.2 | lncRNA | 3.418302835 | 3.25E-07 | 4.67E-06 |
| LINC01121 | lncRNA | 2.807253756 | 3.33E-07 | 4.74E-06 |
| LINC02152 | lncRNA | 3.119646339 | 3.43E-07 | 4.86E-06 |
| AC091133.3 | lncRNA | 3.429525187 | 3.49E-07 | 4.91E-06 |
| LINC02438 | lncRNA | 2.979004903 | 3.69E-07 | 5.16E-06 |
| AC010735.1 | lncRNA | 2.3081537 | 3.81E-07 | 5.32E-06 |
| AC112493.1 | lncRNA | 3.29093487 | 4.07E-07 | 5.63E-06 |
| LINC01535 | lncRNA | 2.611629406 | 4.09E-07 | 5.65E-06 |
| AC125603.1 | lncRNA | 3.651213951 | 4.17E-07 | 5.75E-06 |
| AC113383.1 | lncRNA | 2.344513571 | 4.18E-07 | 5.75E-06 |
| AC103808.3 | lncRNA | 3.198103135 | 4.23E-07 | 5.79E-06 |
| SATB2-AS1 | lncRNA | 2.320196078 | 4.40E-07 | 5.98E-06 |
| LINC02005 | lncRNA | -2.797670082 | 4.66E-07 | 6.31E-06 |
| AC114401.1 | lncRNA | 3.087830686 | 4.79E-07 | 6.46E-06 |
| AL358613.2 | lncRNA | -2.336631231 | 4.82E-07 | 6.49E-06 |
| AC116025.1 | lncRNA | -2.348179413 | 4.94E-07 | 6.61E-06 |
| PRR7-AS1 | lncRNA | 2.296979633 | 4.94E-07 | 6.61E-06 |
| AL162582.1 | lncRNA | 3.312133418 | 5.03E-07 | 6.70E-06 |
| DIO3OS | lncRNA | -3.107227181 | 5.24E-07 | 6.95E-06 |
| AC013553.3 | lncRNA | 2.365650864 | 5.41E-07 | 7.14E-06 |
| AC122710.1 | lncRNA | 4.326525235 | 5.47E-07 | 7.19E-06 |
| AP000851.2 | lncRNA | -2.782249159 | 5.49E-07 | 7.21E-06 |
| AC106820.5 | lncRNA | 2.216831784 | 5.55E-07 | 7.27E-06 |
| DISC1FP1 | lncRNA | -2.310604578 | 5.62E-07 | 7.35E-06 |
| AC005670.1 | lncRNA | 2.279767183 | 5.73E-07 | 7.45E-06 |
| AL603840.1 | lncRNA | -2.378718093 | 5.87E-07 | 7.60E-06 |
| AC105384.1 | lncRNA | -2.40098621 | 6.14E-07 | 7.93E-06 |
| AC116351.1 | lncRNA | -2.200791899 | 6.17E-07 | 7.95E-06 |
| AP000553.1 | lncRNA | 2.302523407 | 6.31E-07 | 8.10E-06 |
| LINC01731 | lncRNA | 3.506498085 | 6.35E-07 | 8.13E-06 |
| CCDC26 | lncRNA | -2.256384702 | 6.41E-07 | 8.20E-06 |
| AL512329.2 | lncRNA | -2.380196075 | 6.55E-07 | 8.36E-06 |
| AC063977.6 | lncRNA | -2.097350745 | 6.62E-07 | 8.43E-06 |
| LINC02313 | lncRNA | 2.877011935 | 6.76E-07 | 8.58E-06 |
| AL359636.2 | lncRNA | 2.741863261 | 6.82E-07 | 8.64E-06 |
| LINC00844 | lncRNA | -3.720288458 | 6.86E-07 | 8.67E-06 |
| DEPDC1-AS1 | lncRNA | 2.740019513 | 7.12E-07 | 8.93E-06 |
| AP005230.1 | lncRNA | 3.008381608 | 7.36E-07 | 9.20E-06 |
| AC100803.1 | lncRNA | 3.479502456 | 7.65E-07 | 9.54E-06 |
| LINC01116 | lncRNA | 2.571750361 | 7.75E-07 | 9.58E-06 |
| AC011503.1 | lncRNA | 3.722699807 | 7.75E-07 | 9.58E-06 |
| AC008549.1 | lncRNA | -3.096271026 | 7.96E-07 | 9.82E-06 |
| AL365259.1 | lncRNA | -2.001068023 | 8.15E-07 | 1.00E-05 |
| AP006285.1 | lncRNA | -2.70630087 | 8.21E-07 | 1.01E-05 |
| AC109439.2 | lncRNA | 3.259901692 | 8.21E-07 | 1.01E-05 |
| AC092115.3 | lncRNA | 3.005187114 | 8.80E-07 | 1.07E-05 |
| AC007298.2 | lncRNA | -2.749317316 | 9.05E-07 | 1.10E-05 |
| KIAA1614-AS1 | lncRNA | 2.361215634 | 9.09E-07 | 1.10E-05 |
| AC025254.1 | lncRNA | 6.73206867 | 9.45E-07 | 1.14E-05 |
| ERVMER61-1 | lncRNA | 4.606240903 | 9.63E-07 | 1.15E-05 |
| AC073321.1 | lncRNA | -2.354183737 | 9.91E-07 | 1.18E-05 |
| LINC02293 | lncRNA | 2.917614522 | 9.92E-07 | 1.18E-05 |
| LINC01554 | lncRNA | -4.171074466 | 1.05E-06 | 1.24E-05 |
| AC003988.1 | lncRNA | -2.473933297 | 1.05E-06 | 1.24E-05 |
| TFAP2A-AS1 | lncRNA | 2.723801857 | 1.06E-06 | 1.26E-05 |
| U47924.3 | lncRNA | 2.088032037 | 1.07E-06 | 1.26E-05 |
| AC009093.5 | lncRNA | -2.819256364 | 1.08E-06 | 1.26E-05 |
| FO393415.1 | lncRNA | -2.154402479 | 1.08E-06 | 1.26E-05 |
| AC141002.1 | lncRNA | 2.174574291 | 1.08E-06 | 1.27E-05 |
| LINC02037 | lncRNA | -2.538475719 | 1.11E-06 | 1.30E-05 |
| FAM212B-AS1 | lncRNA | 2.316889003 | 1.14E-06 | 1.32E-05 |
| AC068987.1 | lncRNA | 2.033469745 | 1.15E-06 | 1.33E-05 |
| LINC01747 | lncRNA | 2.22066587 | 1.27E-06 | 1.45E-05 |
| AL121721.1 | lncRNA | 3.633619827 | 1.28E-06 | 1.46E-05 |
| AC022031.2 | lncRNA | 3.692318144 | 1.28E-06 | 1.46E-05 |
| AC011611.3 | lncRNA | -2.391107125 | 1.30E-06 | 1.48E-05 |
| LINC00898 | lncRNA | 3.161021733 | 1.40E-06 | 1.58E-05 |
| AC006058.1 | lncRNA | -2.679531879 | 1.50E-06 | 1.67E-05 |
| AC103740.1 | lncRNA | -2.171510051 | 1.54E-06 | 1.71E-05 |
| AC104024.1 | lncRNA | -2.277947693 | 1.58E-06 | 1.74E-05 |
| AC007495.1 | lncRNA | -2.505307556 | 1.61E-06 | 1.77E-05 |
| LINC00114 | lncRNA | 2.622154546 | 1.72E-06 | 1.88E-05 |
| AL583808.1 | lncRNA | 3.233219107 | 1.80E-06 | 1.96E-05 |
| KLHL6-AS1 | lncRNA | -2.687834236 | 1.91E-06 | 2.06E-05 |
| LINC02362 | lncRNA | -2.475752057 | 1.94E-06 | 2.10E-05 |
| AC104590.1 | lncRNA | 2.235017978 | 1.99E-06 | 2.13E-05 |
| LINC01611 | lncRNA | 6.189619705 | 2.01E-06 | 2.15E-05 |
| DLX2-AS1 | lncRNA | 2.980251623 | 2.14E-06 | 2.27E-05 |
| AC091133.2 | lncRNA | 2.906615696 | 2.21E-06 | 2.34E-05 |
| AC005722.3 | lncRNA | 3.354163152 | 2.22E-06 | 2.35E-05 |
| AC245100.1 | lncRNA | 2.391046563 | 2.29E-06 | 2.42E-05 |
| AC069294.1 | lncRNA | -2.758992696 | 2.32E-06 | 2.44E-05 |
| LINC01666 | lncRNA | 3.021853725 | 2.33E-06 | 2.44E-05 |
| LINC01511 | lncRNA | 2.769271652 | 2.33E-06 | 2.44E-05 |
| AC078909.2 | lncRNA | 2.192915232 | 2.34E-06 | 2.44E-05 |
| LINC01192 | lncRNA | 3.144419171 | 2.37E-06 | 2.47E-05 |
| LINC00485 | lncRNA | 2.25455776 | 2.38E-06 | 2.47E-05 |
| AL161645.1 | lncRNA | -2.529376947 | 2.42E-06 | 2.50E-05 |
| LINC01297 | lncRNA | 3.21790859 | 2.58E-06 | 2.64E-05 |
| AL162431.1 | lncRNA | 2.593177407 | 2.59E-06 | 2.64E-05 |
| AC034213.1 | lncRNA | 3.516471606 | 2.69E-06 | 2.72E-05 |
| AP001198.2 | lncRNA | -2.728984724 | 2.75E-06 | 2.77E-05 |
| Z99289.1 | lncRNA | -2.16016539 | 2.78E-06 | 2.80E-05 |
| LINC01010 | lncRNA | -2.126893259 | 2.86E-06 | 2.86E-05 |
| AL592182.2 | lncRNA | -2.975830225 | 2.93E-06 | 2.92E-05 |
| AC004221.1 | lncRNA | 2.640334073 | 2.96E-06 | 2.95E-05 |
| LINC01979 | lncRNA | -2.196240877 | 3.10E-06 | 3.08E-05 |
| MIR663AHG | lncRNA | 3.332199595 | 3.15E-06 | 3.11E-05 |
| LINC02327 | lncRNA | 3.85604165 | 3.20E-06 | 3.15E-05 |
| TMEM132D-AS1 | lncRNA | 4.318065501 | 3.20E-06 | 3.15E-05 |
| LINC01814 | lncRNA | -2.662891943 | 3.21E-06 | 3.16E-05 |
| LINC02109 | lncRNA | 3.034177305 | 3.34E-06 | 3.28E-05 |
| AC007991.3 | lncRNA | -2.515103441 | 3.47E-06 | 3.38E-05 |
| AC122694.1 | lncRNA | 2.732722855 | 3.48E-06 | 3.38E-05 |
| AC005381.1 | lncRNA | 3.373186645 | 3.50E-06 | 3.41E-05 |
| AC017002.3 | lncRNA | 2.041384569 | 3.58E-06 | 3.47E-05 |
| AL138974.1 | lncRNA | 3.439540316 | 3.80E-06 | 3.66E-05 |
| AC234772.2 | lncRNA | 2.51549825 | 3.91E-06 | 3.75E-05 |
| AC002351.1 | lncRNA | 3.220149436 | 3.97E-06 | 3.80E-05 |
| TEX41 | lncRNA | 2.485013627 | 3.98E-06 | 3.80E-05 |
| AL353148.1 | lncRNA | -2.352482834 | 3.99E-06 | 3.80E-05 |
| HAR1A | lncRNA | -2.337916272 | 4.04E-06 | 3.83E-05 |
| LDLRAD4-AS1 | lncRNA | -2.134805598 | 4.14E-06 | 3.91E-05 |
| AC021491.2 | lncRNA | 2.238368193 | 4.35E-06 | 4.10E-05 |
| PWRN1 | lncRNA | -2.646093135 | 4.54E-06 | 4.27E-05 |
| AL109933.1 | lncRNA | -2.333128467 | 4.63E-06 | 4.34E-05 |
| LINC02160 | lncRNA | -2.966754485 | 4.72E-06 | 4.41E-05 |
| FALEC | lncRNA | 2.030039246 | 5.04E-06 | 4.69E-05 |
| LINC01338 | lncRNA | 3.225620577 | 5.20E-06 | 4.84E-05 |
| AC099520.1 | lncRNA | 5.256584003 | 5.27E-06 | 4.90E-05 |
| LINC01257 | lncRNA | 3.290297517 | 5.43E-06 | 5.03E-05 |
| AC100872.1 | lncRNA | 2.669740049 | 5.47E-06 | 5.05E-05 |
| IGF2BP2-AS1 | lncRNA | 2.574982939 | 5.57E-06 | 5.13E-05 |
| AC110285.1 | lncRNA | 2.05316872 | 5.61E-06 | 5.16E-05 |
| LINC01186 | lncRNA | 2.401399317 | 5.92E-06 | 5.42E-05 |
| LINC01750 | lncRNA | -2.016792876 | 6.15E-06 | 5.60E-05 |
| AC004870.2 | lncRNA | 4.396373997 | 6.41E-06 | 5.78E-05 |
| AC012379.1 | lncRNA | -2.570346735 | 6.47E-06 | 5.80E-05 |
| AC020934.1 | lncRNA | 2.539742859 | 6.53E-06 | 5.83E-05 |
| AC024581.1 | lncRNA | 2.792873231 | 6.62E-06 | 5.91E-05 |
| ADGRD1-AS1 | lncRNA | 3.948249951 | 6.93E-06 | 6.16E-05 |
| AC010333.2 | lncRNA | 3.318869427 | 7.01E-06 | 6.21E-05 |
| AL357033.2 | lncRNA | -2.487423825 | 7.21E-06 | 6.36E-05 |
| AL353693.1 | lncRNA | -2.344051792 | 7.50E-06 | 6.57E-05 |
| AL445209.1 | lncRNA | 2.874914696 | 7.72E-06 | 6.75E-05 |
| FAM30A | lncRNA | -2.541415393 | 7.92E-06 | 6.91E-05 |
| AL358216.1 | lncRNA | -2.211387518 | 7.98E-06 | 6.96E-05 |
| AL121790.1 | lncRNA | -2.283666246 | 8.14E-06 | 7.06E-05 |
| AC010255.1 | lncRNA | 2.931487747 | 8.15E-06 | 7.06E-05 |
| LRP4-AS1 | lncRNA | 2.021645693 | 8.15E-06 | 7.06E-05 |
| LINC00664 | lncRNA | 2.075294492 | 8.31E-06 | 7.17E-05 |
| AC022424.1 | lncRNA | 3.573902543 | 8.66E-06 | 7.44E-05 |
| HNF4A-AS1 | lncRNA | -2.151717716 | 8.66E-06 | 7.44E-05 |
| MIR217HG | lncRNA | 2.749992691 | 8.89E-06 | 7.60E-05 |
| AL353801.1 | lncRNA | 2.085311431 | 9.00E-06 | 7.68E-05 |
| TLX1NB | lncRNA | 2.628010721 | 9.06E-06 | 7.72E-05 |
| LINC01956 | lncRNA | 3.92314363 | 9.26E-06 | 7.85E-05 |
| AC037198.3 | lncRNA | -2.216816129 | 1.01E-05 | 8.43E-05 |
| AC009292.1 | lncRNA | 2.611428979 | 1.05E-05 | 8.72E-05 |
| AP003555.3 | lncRNA | -2.40416665 | 1.09E-05 | 9.03E-05 |
| AL109615.3 | lncRNA | 2.329049748 | 1.17E-05 | 9.63E-05 |
| HOXA-AS3 | lncRNA | 2.671536189 | 1.21E-05 | 9.88E-05 |
| AC089983.1 | lncRNA | 2.95753673 | 1.23E-05 | 0.0001 |
| AC092625.1 | lncRNA | 5.206250322 | 1.24E-05 | 0.0001 |
| AC010894.3 | lncRNA | 2.831637751 | 1.26E-05 | 0.000101 |
| AC245041.2 | lncRNA | -2.433778475 | 1.27E-05 | 0.000102 |
| RMST | lncRNA | 2.614671722 | 1.33E-05 | 0.000107 |
| LINC01117 | lncRNA | 2.712414753 | 1.40E-05 | 0.000112 |
| LINC01426 | lncRNA | 2.02719534 | 1.41E-05 | 0.000112 |
| AL033381.2 | lncRNA | 2.891033517 | 1.42E-05 | 0.000113 |
| BX322234.2 | lncRNA | 2.965067764 | 1.43E-05 | 0.000113 |
| AC093515.1 | lncRNA | 4.94387759 | 1.50E-05 | 0.000118 |
| U91319.1 | lncRNA | -2.829096783 | 1.51E-05 | 0.000119 |
| LINC01831 | lncRNA | -3.255903337 | 1.53E-05 | 0.000119 |
| LINC02466 | lncRNA | 4.805969918 | 1.53E-05 | 0.000119 |
| AC090340.1 | lncRNA | -2.878916696 | 1.54E-05 | 0.00012 |
| AC104088.1 | lncRNA | 3.217335177 | 1.61E-05 | 0.000125 |
| SNHG27 | lncRNA | 3.292992847 | 1.70E-05 | 0.00013 |
| AC114485.1 | lncRNA | 5.217674011 | 1.73E-05 | 0.000132 |
| LINC02082 | lncRNA | 2.869082355 | 1.75E-05 | 0.000133 |
| AL365226.2 | lncRNA | 3.325561493 | 1.80E-05 | 0.000137 |
| CLRN1-AS1 | lncRNA | -2.590144805 | 1.80E-05 | 0.000137 |
| LINC01018 | lncRNA | -3.303435975 | 1.89E-05 | 0.000143 |
| LINC01146 | lncRNA | -2.284169732 | 1.94E-05 | 0.000146 |
| AL359636.1 | lncRNA | 2.447402798 | 2.00E-05 | 0.00015 |
| AC009121.1 | lncRNA | 2.235609266 | 2.01E-05 | 0.00015 |
| AC007639.1 | lncRNA | 2.872806023 | 2.02E-05 | 0.000151 |
| AC138965.2 | lncRNA | 2.932245943 | 2.19E-05 | 0.000161 |
| AC005304.2 | lncRNA | -2.223180623 | 2.22E-05 | 0.000164 |
| LINC02105 | lncRNA | 2.760117116 | 2.31E-05 | 0.000169 |
| MIR4290HG | lncRNA | -2.366929366 | 2.34E-05 | 0.00017 |
| AC023090.1 | lncRNA | 3.234182679 | 2.35E-05 | 0.000171 |
| LINC02154 | lncRNA | 2.942949286 | 2.42E-05 | 0.000175 |
| AC007402.1 | lncRNA | 2.839490317 | 2.50E-05 | 0.000179 |
| AC007277.1 | lncRNA | 2.32345566 | 2.58E-05 | 0.000184 |
| CU104787.1 | lncRNA | 3.088156051 | 2.59E-05 | 0.000184 |
| AL163953.1 | lncRNA | 2.410303121 | 2.60E-05 | 0.000185 |
| AP000593.3 | lncRNA | 2.641146177 | 2.68E-05 | 0.00019 |
| AC079598.1 | lncRNA | -2.744753375 | 2.68E-05 | 0.00019 |
| ZNF385D-AS1 | lncRNA | 4.757703274 | 2.71E-05 | 0.000192 |
| AC107294.1 | lncRNA | -2.057612998 | 2.87E-05 | 0.0002 |
| AC069061.2 | lncRNA | 4.858505356 | 3.15E-05 | 0.000218 |
| AC007614.4 | lncRNA | 2.074457552 | 3.19E-05 | 0.00022 |
| AC245884.11 | lncRNA | -2.03672715 | 3.36E-05 | 0.000229 |
| AL137026.2 | lncRNA | 3.058272398 | 3.39E-05 | 0.000231 |
| SLC7A11-AS1 | lncRNA | 2.547267726 | 3.43E-05 | 0.000233 |
| AC079380.1 | lncRNA | 2.199788424 | 3.50E-05 | 0.000237 |
| AC006206.2 | lncRNA | 3.240786893 | 3.67E-05 | 0.000246 |
| AC068875.1 | lncRNA | -2.001202907 | 3.77E-05 | 0.000251 |
| AL928742.1 | lncRNA | -2.012873234 | 3.97E-05 | 0.000262 |
| TTC39A-AS1 | lncRNA | 2.356618132 | 3.98E-05 | 0.000262 |
| LINC01202 | lncRNA | 4.429966711 | 3.98E-05 | 0.000262 |
| AC009121.2 | lncRNA | 2.014696722 | 4.00E-05 | 0.000264 |
| TRPC7-AS1 | lncRNA | 2.382612381 | 4.02E-05 | 0.000264 |
| AC139769.2 | lncRNA | 3.671406335 | 4.02E-05 | 0.000264 |
| AL121827.1 | lncRNA | -2.485575034 | 4.12E-05 | 0.000267 |
| AC025265.3 | lncRNA | 2.508762584 | 4.20E-05 | 0.000271 |
| AC007207.2 | lncRNA | -3.125316983 | 4.29E-05 | 0.000275 |
| AC084782.2 | lncRNA | 2.617974899 | 4.52E-05 | 0.000289 |
| LINC00582 | lncRNA | -2.184787472 | 4.53E-05 | 0.000289 |
| AC099552.1 | lncRNA | 4.616770013 | 4.64E-05 | 0.000295 |
| AC024651.1 | lncRNA | -2.203590082 | 4.85E-05 | 0.000308 |
| LINC00648 | lncRNA | 3.724672955 | 4.99E-05 | 0.000316 |
| AC011840.1 | lncRNA | 3.410485447 | 5.00E-05 | 0.000316 |
| AC092747.1 | lncRNA | -2.063072985 | 5.14E-05 | 0.000323 |
| AC113145.1 | lncRNA | 4.633839434 | 5.24E-05 | 0.000328 |
| AP005057.1 | lncRNA | 2.504121687 | 5.36E-05 | 0.000335 |
| AC006557.1 | lncRNA | 2.104798826 | 5.40E-05 | 0.000337 |
| AC112495.1 | lncRNA | -2.279596253 | 5.55E-05 | 0.000344 |
| AC073352.1 | lncRNA | 2.003219559 | 5.59E-05 | 0.000346 |
| LINC01681 | lncRNA | 3.272803282 | 5.62E-05 | 0.000347 |
| LINC01448 | lncRNA | 2.81512132 | 5.98E-05 | 0.000367 |
| LINC02119 | lncRNA | 4.291621971 | 6.04E-05 | 0.00037 |
| LINC02505 | lncRNA | 2.816507214 | 6.15E-05 | 0.000376 |
| AP000785.1 | lncRNA | -2.188795428 | 6.21E-05 | 0.000379 |
| AP000997.2 | lncRNA | 2.43818078 | 6.28E-05 | 0.000382 |
| LINC01258 | lncRNA | 2.515859818 | 6.50E-05 | 0.000395 |
| AC016705.2 | lncRNA | -2.362348634 | 6.77E-05 | 0.000409 |
| MYCNOS | lncRNA | 2.258643768 | 7.13E-05 | 0.000427 |
| LHFPL3-AS1 | lncRNA | 2.670655576 | 7.14E-05 | 0.000427 |
| AC126175.1 | lncRNA | 2.577335492 | 7.22E-05 | 0.000431 |
| PART1 | lncRNA | 4.091788824 | 7.43E-05 | 0.00044 |
| AC022784.6 | lncRNA | -2.388113593 | 7.45E-05 | 0.000441 |
| AP003900.1 | lncRNA | 4.188612867 | 7.55E-05 | 0.000447 |
| LINC01793 | lncRNA | 3.40137412 | 7.75E-05 | 0.000457 |
| PINCR | lncRNA | 3.325859298 | 7.77E-05 | 0.000458 |
| AC108865.1 | lncRNA | 2.809514943 | 7.93E-05 | 0.000466 |
| AL078612.2 | lncRNA | 2.332339151 | 8.12E-05 | 0.000474 |
| AC096637.2 | lncRNA | 2.014734417 | 8.22E-05 | 0.000478 |
| SACS-AS1 | lncRNA | 3.575743181 | 8.23E-05 | 0.000479 |
| LINC01967 | lncRNA | 2.312211482 | 8.56E-05 | 0.000495 |
| AP000439.2 | lncRNA | -2.194346249 | 8.61E-05 | 0.000497 |
| AL355596.1 | lncRNA | 2.861262025 | 8.63E-05 | 0.000498 |
| MYHAS | lncRNA | 2.6491236 | 8.78E-05 | 0.000506 |
| AC007423.1 | lncRNA | -3.200864091 | 8.87E-05 | 0.00051 |
| LINC02128 | lncRNA | -2.211735509 | 9.02E-05 | 0.000517 |
| AC074286.1 | lncRNA | 2.136189041 | 9.02E-05 | 0.000517 |
| WASIR2 | lncRNA | 2.076602905 | 9.32E-05 | 0.000531 |
| LINC01992 | lncRNA | 2.96874449 | 9.55E-05 | 0.000541 |
| AC245041.1 | lncRNA | -2.026224547 | 9.67E-05 | 0.000547 |
| AC108136.1 | lncRNA | 2.124467097 | 9.69E-05 | 0.000547 |
| LINC00519 | lncRNA | 2.145413762 | 0.000101 | 0.000566 |
| AC008060.4 | lncRNA | 4.256361552 | 0.000102 | 0.000573 |
| MIR3150BHG | lncRNA | 2.082493149 | 0.000104 | 0.000582 |
| AL445647.1 | lncRNA | 2.693916216 | 0.000106 | 0.000591 |
| AC090709.1 | lncRNA | 3.642806895 | 0.000109 | 0.000604 |
| PITPNM2-AS1 | lncRNA | -2.269829647 | 0.000109 | 0.000605 |
| LINC00668 | lncRNA | 2.949438662 | 0.000119 | 0.000651 |
| BBOX1-AS1 | lncRNA | 2.310375053 | 0.000119 | 0.000653 |
| LINC01649 | lncRNA | 2.470027482 | 0.000121 | 0.000657 |
| AL139275.2 | lncRNA | 3.907222403 | 0.000121 | 0.000658 |
| HOXC-AS2 | lncRNA | 2.341315105 | 0.000121 | 0.00066 |
| AC015712.1 | lncRNA | -2.011029584 | 0.000128 | 0.000693 |
| AC068675.1 | lncRNA | 2.247844091 | 0.000129 | 0.000695 |
| NCAM1-AS1 | lncRNA | 2.157987611 | 0.000132 | 0.00071 |
| LINC01468 | lncRNA | 3.719077189 | 0.000133 | 0.000711 |
| AC006504.7 | lncRNA | -2.57520283 | 0.000136 | 0.000727 |
| AL450322.2 | lncRNA | 2.393513863 | 0.000138 | 0.000736 |
| AC092447.8 | lncRNA | 3.444312293 | 0.000138 | 0.000736 |
| LINC02348 | lncRNA | -2.323859493 | 0.000141 | 0.00075 |
| AC010880.1 | lncRNA | 3.349380755 | 0.000144 | 0.000764 |
| AL133370.1 | lncRNA | 2.332403588 | 0.000146 | 0.000772 |
| AC104129.1 | lncRNA | -2.014480517 | 0.000146 | 0.000775 |
| AC108865.2 | lncRNA | 2.671426448 | 0.00015 | 0.00079 |
| LINC00689 | lncRNA | 2.279552343 | 0.000153 | 0.000806 |
| AC074135.1 | lncRNA | 2.498597474 | 0.000155 | 0.000818 |
| LINC02323 | lncRNA | 2.278096911 | 0.000161 | 0.000841 |
| AC008991.2 | lncRNA | 3.631575809 | 0.000161 | 0.000844 |
| LINC02058 | lncRNA | 3.782757502 | 0.000169 | 0.000879 |
| AC048383.1 | lncRNA | 2.08278347 | 0.000172 | 0.00089 |
| AC113346.1 | lncRNA | 2.490796469 | 0.000172 | 0.00089 |
| LINC00922 | lncRNA | 2.660966706 | 0.000177 | 0.00091 |
| AC078993.1 | lncRNA | 2.378907267 | 0.000178 | 0.000913 |
| LINC01269 | lncRNA | 2.286913245 | 0.00018 | 0.000923 |
| AC007036.1 | lncRNA | 2.218390239 | 0.000187 | 0.000955 |
| LINC00511 | lncRNA | 2.084723908 | 0.000188 | 0.00096 |
| AC026320.1 | lncRNA | 3.646292347 | 0.000189 | 0.000962 |
| AC006946.2 | lncRNA | 2.42194579 | 0.00019 | 0.000964 |
| LINC00595 | lncRNA | 2.26361795 | 0.000198 | 0.001 |
| TCL6 | lncRNA | 2.005101606 | 0.000205 | 0.001028 |
| AP005271.1 | lncRNA | 2.43575727 | 0.000206 | 0.001031 |
| AC113404.1 | lncRNA | 2.343841018 | 0.000207 | 0.001039 |
| ZFPM2-AS1 | lncRNA | 2.197728444 | 0.000208 | 0.001043 |
| DSCR4-IT1 | lncRNA | 3.744204515 | 0.000216 | 0.001077 |
| LINC02418 | lncRNA | -2.343694626 | 0.000218 | 0.001086 |
| AP003181.1 | lncRNA | 3.388993683 | 0.000221 | 0.001097 |
| AL157714.2 | lncRNA | 2.718710167 | 0.000222 | 0.0011 |
| LINC01920 | lncRNA | -2.361887831 | 0.000228 | 0.001125 |
| AC008443.3 | lncRNA | 2.406622504 | 0.000232 | 0.00114 |
| LINC02432 | lncRNA | -2.227959585 | 0.000244 | 0.001189 |
| AC107973.1 | lncRNA | 3.564731189 | 0.000247 | 0.001202 |
| IGF2-AS | lncRNA | -2.370216471 | 0.000249 | 0.001208 |
| HOXC-AS1 | lncRNA | 3.478404874 | 0.000259 | 0.001252 |
| LINC01804 | lncRNA | 2.754245822 | 0.000265 | 0.001277 |
| LINC00942 | lncRNA | 2.920818917 | 0.000266 | 0.00128 |
| LINC00551 | lncRNA | -2.10669666 | 0.000268 | 0.001291 |
| LINC02269 | lncRNA | 3.401889344 | 0.000282 | 0.001346 |
| AL357153.1 | lncRNA | 2.415947551 | 0.000293 | 0.00139 |
| PKIA-AS1 | lncRNA | 2.291200295 | 0.000294 | 0.001394 |
| LINC01231 | lncRNA | 3.430569731 | 0.000294 | 0.001394 |
| AC011676.1 | lncRNA | 2.199895379 | 0.000309 | 0.001447 |
| BX547991.1 | lncRNA | -2.030151359 | 0.000315 | 0.001467 |
| C5orf66-AS1 | lncRNA | 2.756585227 | 0.00033 | 0.001529 |
| AC018814.1 | lncRNA | 2.643086169 | 0.000332 | 0.001537 |
| AL591501.1 | lncRNA | 3.30970285 | 0.000349 | 0.001603 |
| LINC01060 | lncRNA | 2.451966888 | 0.000362 | 0.001646 |
| AC106892.1 | lncRNA | 3.023741531 | 0.000365 | 0.001657 |
| AC003985.2 | lncRNA | 3.429949488 | 0.00037 | 0.001678 |
| C10orf71-AS1 | lncRNA | 3.124421328 | 0.000377 | 0.001706 |
| LINC01242 | lncRNA | 3.166159175 | 0.000382 | 0.001729 |
| AC093702.1 | lncRNA | 2.064395062 | 0.000382 | 0.001729 |
| LINC01738 | lncRNA | 2.267358212 | 0.000412 | 0.001846 |
| AC079466.1 | lncRNA | 3.230248716 | 0.000418 | 0.001868 |
| AL353740.1 | lncRNA | -2.213749965 | 0.000418 | 0.001868 |
| AC024563.1 | lncRNA | 2.746290104 | 0.000423 | 0.001887 |
| DLX6-AS1 | lncRNA | 3.10994739 | 0.000425 | 0.001893 |
| AL954650.1 | lncRNA | 2.595915588 | 0.000432 | 0.00192 |
| LINC02307 | lncRNA | 3.11875028 | 0.000433 | 0.001921 |
| LINC01297 | lncRNA | 2.261331369 | 0.000437 | 0.001938 |
| SRGAP3-AS4 | lncRNA | 2.33365033 | 0.000442 | 0.001958 |
| FLJ36000 | lncRNA | 3.044611774 | 0.000479 | 0.002101 |
| AC104134.1 | lncRNA | 2.057829464 | 0.0005 | 0.002185 |
| AC109454.3 | lncRNA | -2.050465138 | 0.000501 | 0.002187 |
| Z93403.1 | lncRNA | 2.268286814 | 0.000503 | 0.002188 |
| AC013451.2 | lncRNA | 2.295098543 | 0.000512 | 0.002223 |
| LINC01436 | lncRNA | 2.104891785 | 0.000568 | 0.002419 |
| LINC02197 | lncRNA | -2.158269795 | 0.000569 | 0.002419 |
| AP005205.2 | lncRNA | 2.286792265 | 0.000571 | 0.002425 |
| AL162726.4 | lncRNA | 2.93001848 | 0.000589 | 0.002486 |
| TRHDE-AS1 | lncRNA | -2.764763881 | 0.000591 | 0.002489 |
| UG0898H09 | lncRNA | -2.203391907 | 0.000617 | 0.002578 |
| LINC02489 | lncRNA | 2.685896653 | 0.000628 | 0.002621 |
| AL121757.1 | lncRNA | 2.095660905 | 0.00063 | 0.002626 |
| LINC01036 | lncRNA | 2.573456144 | 0.000632 | 0.002628 |
| AL121970.1 | lncRNA | 2.275307437 | 0.00064 | 0.002658 |
| AC090116.1 | lncRNA | 2.19419019 | 0.000675 | 0.002774 |
| AC023824.6 | lncRNA | 3.199424151 | 0.000688 | 0.002814 |
| AC108868.2 | lncRNA | 2.894574709 | 0.000689 | 0.002814 |
| AC012339.1 | lncRNA | 2.869188255 | 0.000689 | 0.002814 |
| AL139002.1 | lncRNA | 2.35173618 | 0.000694 | 0.002834 |
| LINC01518 | lncRNA | 2.817176065 | 0.000697 | 0.002841 |
| LINC00160 | lncRNA | 2.101604409 | 0.0007 | 0.002852 |
| LINC02008 | lncRNA | 2.344892309 | 0.000703 | 0.002862 |
| AC008109.1 | lncRNA | 2.301120741 | 0.000715 | 0.002907 |
| AL359551.1 | lncRNA | 2.619305297 | 0.000747 | 0.003011 |
| AC009271.1 | lncRNA | 2.848908719 | 0.00075 | 0.003019 |
| AC011591.1 | lncRNA | -2.107710286 | 0.000817 | 0.003246 |
| AP004147.1 | lncRNA | 2.309108569 | 0.00082 | 0.003254 |
| AC114803.1 | lncRNA | 2.06827375 | 0.000821 | 0.003255 |
| AL133372.2 | lncRNA | 2.186286738 | 0.000846 | 0.00334 |
| AC018558.1 | lncRNA | 2.741631323 | 0.000861 | 0.003378 |
| LINC01143 | lncRNA | 2.109862232 | 0.00087 | 0.00341 |
| AC131902.1 | lncRNA | 2.349441374 | 0.000913 | 0.003554 |
| LINC01639 | lncRNA | -2.252606186 | 0.000924 | 0.003591 |
| AC106895.1 | lncRNA | 2.676861091 | 0.000954 | 0.003689 |
| UPK1A-AS1 | lncRNA | 2.181994896 | 0.000973 | 0.00374 |
| EGLN3-AS1 | lncRNA | 2.626537543 | 0.000989 | 0.003792 |
| LINC01164 | lncRNA | 2.718895655 | 0.00106 | 0.004037 |
| AC144833.1 | lncRNA | 2.443752075 | 0.001082 | 0.004102 |
| AC068756.1 | lncRNA | 2.507829432 | 0.001087 | 0.004121 |
| AL110292.1 | lncRNA | 2.031342996 | 0.001126 | 0.004247 |
| OVAAL | lncRNA | 2.137119496 | 0.001127 | 0.004247 |
| AP001599.1 | lncRNA | 2.538219975 | 0.001181 | 0.004394 |
| AC104041.1 | lncRNA | 2.063497632 | 0.001199 | 0.004452 |
| Z82214.2 | lncRNA | 2.415564511 | 0.001201 | 0.004456 |
| AL157931.1 | lncRNA | 2.367330334 | 0.001204 | 0.004462 |
| AC124290.1 | lncRNA | 2.527742058 | 0.001218 | 0.004511 |
| AC092447.5 | lncRNA | 2.546240652 | 0.001247 | 0.004603 |
| AC007848.1 | lncRNA | 2.019088966 | 0.001253 | 0.004618 |
| AL590705.2 | lncRNA | 2.53115102 | 0.001261 | 0.004637 |
| LINC02365 | lncRNA | 2.443622037 | 0.001284 | 0.00471 |
| AC022092.1 | lncRNA | 2.01929958 | 0.001302 | 0.004762 |
| LINC00351 | lncRNA | 2.466630728 | 0.00136 | 0.004937 |
| AC007099.1 | lncRNA | 2.176187611 | 0.001416 | 0.0051 |
| AL591686.1 | lncRNA | 2.719651901 | 0.001459 | 0.005225 |
| AC128689.1 | lncRNA | 2.09763559 | 0.001481 | 0.005293 |
| C8orf87 | lncRNA | 2.282436511 | 0.001538 | 0.005467 |
| LINC01579 | lncRNA | 2.322906515 | 0.001572 | 0.005571 |
| AC004053.1 | lncRNA | -2.272507961 | 0.001613 | 0.005702 |
| Z82249.1 | lncRNA | 3.307541442 | 0.00169 | 0.005934 |
| AC092490.1 | lncRNA | 2.368199232 | 0.001704 | 0.005976 |
| LINC02055 | lncRNA | 2.208205802 | 0.001742 | 0.006091 |
| AP000696.1 | lncRNA | 2.326767084 | 0.001761 | 0.006146 |
| SRGAP3-AS2 | lncRNA | 2.308835413 | 0.001835 | 0.00636 |
| SSTR5-AS1 | lncRNA | 2.546087353 | 0.001855 | 0.006422 |
| LINC01854 | lncRNA | 2.164467424 | 0.001877 | 0.006488 |
| LINC01951 | lncRNA | 2.199530261 | 0.001915 | 0.006603 |
| AL162725.2 | lncRNA | 2.052526229 | 0.001963 | 0.006744 |
| AL035045.1 | lncRNA | 2.211426968 | 0.001973 | 0.006757 |
| AL353581.1 | lncRNA | 2.113223585 | 0.001973 | 0.006757 |
| AC007064.2 | lncRNA | 2.132449072 | 0.002039 | 0.006926 |
| AC019183.1 | lncRNA | 2.057804216 | 0.002134 | 0.007193 |
| LINC02258 | lncRNA | 2.262683841 | 0.002137 | 0.007197 |
| AC099792.1 | lncRNA | 2.074171991 | 0.002178 | 0.007303 |
| AP001783.1 | lncRNA | -2.692830668 | 0.002234 | 0.00746 |
| LINC01792 | lncRNA | 2.062545176 | 0.002279 | 0.007587 |
| AC096746.1 | lncRNA | 2.180567544 | 0.002367 | 0.007822 |
| LINC02469 | lncRNA | 2.164599697 | 0.002431 | 0.007986 |
| LINC00601 | lncRNA | 2.937321771 | 0.002577 | 0.008373 |
| LINC00602 | lncRNA | 2.13995307 | 0.002582 | 0.008385 |
| AC097512.1 | lncRNA | 2.230893561 | 0.002716 | 0.008749 |
| hsa-miR-490 | miRNA | -5.97124 | 2.06E-21 | 1.39E-18 |
| hsa-miR-4686 | miRNA | -4.76904 | 8.10E-19 | 2.24E-16 |
| hsa-miR-767 | miRNA | 8.365187 | 9.98E-19 | 2.24E-16 |
| hsa-miR-10b | miRNA | 4.073701 | 2.48E-17 | 4.17E-15 |
| hsa-miR-105-1 | miRNA | 7.730002 | 2.85E-16 | 3.83E-14 |
| hsa-miR-1258 | miRNA | -4.80732 | 3.02E-15 | 2.45E-13 |
| hsa-miR-139 | miRNA | -2.16368 | 3.29E-15 | 2.45E-13 |
| hsa-miR-4652 | miRNA | 5.941911 | 5.45E-15 | 3.66E-13 |
| hsa-miR-3677 | miRNA | 2.08624 | 1.21E-14 | 7.36E-13 |
| hsa-miR-105-2 | miRNA | 7.728965 | 2.08E-14 | 1.07E-12 |
| hsa-miR-675 | miRNA | -3.60098 | 1.38E-12 | 5.80E-11 |
| hsa-miR-4746 | miRNA | 2.225613 | 2.64E-12 | 1.04E-10 |
| hsa-miR-1251 | miRNA | 4.619528 | 4.69E-11 | 1.43E-09 |
| hsa-miR-383 | miRNA | -3.95871 | 6.69E-11 | 1.87E-09 |
| hsa-miR-891a | miRNA | 4.683075 | 1.72E-10 | 3.98E-09 |
| hsa-miR-1269a | miRNA | 4.932307 | 2.50E-09 | 4.43E-08 |
| hsa-miR-452 | miRNA | 2.19703 | 2.93E-09 | 5.06E-08 |
| hsa-miR-760 | miRNA | 2.193998 | 3.01E-09 | 5.06E-08 |
| hsa-miR-3144 | miRNA | 3.333529 | 3.08E-09 | 5.06E-08 |
| hsa-miR-199a-2 | miRNA | -2.35699 | 3.71E-09 | 5.60E-08 |
| hsa-miR-34c | miRNA | 2.658257 | 3.75E-09 | 5.60E-08 |
| hsa-miR-199a-1 | miRNA | -2.36603 | 3.98E-09 | 5.78E-08 |
| hsa-miR-1269b | miRNA | 4.836019 | 4.13E-09 | 5.78E-08 |
| hsa-miR-183 | miRNA | 3.126613 | 7.02E-09 | 8.58E-08 |
| hsa-miR-199b | miRNA | -2.30473 | 7.58E-09 | 8.94E-08 |
| hsa-miR-520c | miRNA | 3.989551 | 1.31E-08 | 1.49E-07 |
| hsa-miR-224 | miRNA | 2.395113 | 1.77E-08 | 1.95E-07 |
| hsa-miR-483 | miRNA | -3.96538 | 3.92E-08 | 3.88E-07 |
| hsa-miR-214 | miRNA | -2.13805 | 7.05E-08 | 6.67E-07 |
| hsa-miR-1266 | miRNA | 2.062702 | 1.13E-07 | 9.96E-07 |
| hsa-miR-96 | miRNA | 2.787827 | 2.25E-07 | 1.87E-06 |
| hsa-miR-182 | miRNA | 2.619371 | 3.59E-07 | 2.90E-06 |
| hsa-miR-190b | miRNA | 2.603496 | 4.18E-07 | 3.31E-06 |
| hsa-miR-135a-2 | miRNA | 3.139236 | 4.77E-07 | 3.69E-06 |
| hsa-miR-187 | miRNA | -2.35097 | 1.09E-06 | 7.45E-06 |
| hsa-miR-765 | miRNA | 2.139491 | 1.25E-06 | 8.42E-06 |
| hsa-miR-508 | miRNA | 2.327641 | 1.94E-06 | 1.22E-05 |
| hsa-miR-135a-1 | miRNA | 2.695865 | 2.58E-06 | 1.59E-05 |
| hsa-miR-466 | miRNA | 3.971953 | 3.30E-06 | 1.96E-05 |
| hsa-miR-514a-2 | miRNA | 2.651504 | 3.33E-06 | 1.96E-05 |
| hsa-miR-509-3 | miRNA | 2.248182 | 6.51E-06 | 3.59E-05 |
| hsa-miR-6718 | miRNA | -2.14223 | 6.60E-06 | 3.61E-05 |
| hsa-miR-621 | miRNA | -2.11639 | 7.12E-06 | 3.82E-05 |
| hsa-miR-509-1 | miRNA | 2.306294 | 7.61E-06 | 4.00E-05 |
| hsa-miR-514a-1 | miRNA | 2.699316 | 1.07E-05 | 5.46E-05 |
| hsa-miR-892a | miRNA | 3.261796 | 1.23E-05 | 6.22E-05 |
| hsa-miR-514a-3 | miRNA | 2.492325 | 1.25E-05 | 6.26E-05 |
| hsa-miR-184 | miRNA | 2.573029 | 1.57E-05 | 7.74E-05 |
| hsa-miR-615 | miRNA | 3.300993 | 3.06E-05 | 0.000137 |
| hsa-miR-34b | miRNA | 2.200961 | 3.41E-05 | 0.000151 |
| hsa-miR-1247 | miRNA | -2.80147 | 4.09E-05 | 0.000176 |
| hsa-miR-520f | miRNA | 3.430619 | 4.43E-05 | 0.00019 |
| hsa-miR-9-2 | miRNA | 2.06224 | 8.19E-05 | 0.000328 |
| hsa-miR-3189 | miRNA | 2.168531 | 8.69E-05 | 0.000346 |
| hsa-miR-5589 | miRNA | -2.10013 | 0.000113 | 0.000434 |
| hsa-miR-524 | miRNA | 2.55368 | 0.000139 | 0.000507 |
| hsa-miR-512-2 | miRNA | 2.835248 | 0.000156 | 0.000557 |
| hsa-miR-520b | miRNA | 2.953654 | 0.000185 | 0.000654 |
| hsa-miR-3923 | miRNA | 2.860218 | 0.00019 | 0.000667 |
| hsa-miR-429 | miRNA | -2.05936 | 0.000207 | 0.000719 |
| hsa-miR-520h | miRNA | 2.805109 | 0.000217 | 0.000747 |
| hsa-miR-512-1 | miRNA | 2.868123 | 0.000247 | 0.000832 |
| hsa-miR-519c | miRNA | 2.494231 | 0.000256 | 0.000856 |
| hsa-miR-520e | miRNA | 2.792632 | 0.000318 | 0.001033 |
| hsa-miR-520a | miRNA | 2.980603 | 0.000448 | 0.001422 |
| hsa-miR-515-2 | miRNA | 2.503036 | 0.000486 | 0.001533 |
| hsa-miR-888 | miRNA | 3.060374 | 0.000727 | 0.002251 |
| hsa-miR-517c | miRNA | 2.388818 | 0.001003 | 0.002955 |
| hsa-miR-521-1 | miRNA | 2.470935 | 0.001025 | 0.003007 |
| hsa-miR-523 | miRNA | 2.216669 | 0.001477 | 0.004171 |
| hsa-miR-891b | miRNA | 2.419317 | 0.001741 | 0.004854 |
| hsa-miR-518b | miRNA | 2.381151 | 0.001755 | 0.004863 |
| hsa-miR-520g | miRNA | 2.504013 | 0.00176 | 0.004863 |
| CLEC1B | mRNA | -9.22639 | 6.28E-29 | 1.09E-24 |
| BMP10 | mRNA | -7.90679 | 2.94E-28 | 2.55E-24 |
| CLEC4G | mRNA | -8.7409 | 1.06E-26 | 6.11E-23 |
| CRHBP | mRNA | -7.01918 | 2.55E-26 | 1.05E-22 |
| GDF2 | mRNA | -9.94035 | 3.02E-26 | 1.05E-22 |
| CLEC4M | mRNA | -9.85143 | 9.94E-26 | 2.88E-22 |
| CENPF | mRNA | 3.578604 | 1.70E-24 | 4.23E-21 |
| MARCO | mRNA | -8.03543 | 3.51E-24 | 7.62E-21 |
| KIF2C | mRNA | 3.496082 | 8.61E-24 | 1.54E-20 |
| ADAMTS13 | mRNA | -3.53464 | 8.88E-24 | 1.54E-20 |
| BMPER | mRNA | -6.39429 | 1.93E-23 | 3.05E-20 |
| UBE2T | mRNA | 3.187348 | 4.04E-23 | 5.74E-20 |
| COLEC10 | mRNA | -6.11939 | 4.29E-23 | 5.74E-20 |
| BIRC5 | mRNA | 3.752193 | 6.52E-23 | 8.09E-20 |
| FCN3 | mRNA | -5.9972 | 2.97E-22 | 3.44E-19 |
| ECM1 | mRNA | -3.98855 | 3.30E-22 | 3.58E-19 |
| KIF4A | mRNA | 3.636275 | 3.97E-22 | 4.06E-19 |
| VIPR1 | mRNA | -4.85795 | 5.90E-22 | 5.70E-19 |
| UBE2C | mRNA | 3.766945 | 6.27E-22 | 5.74E-19 |
| CDKN3 | mRNA | 3.560056 | 8.15E-22 | 6.80E-19 |
| STAB2 | mRNA | -6.96007 | 8.22E-22 | 6.80E-19 |
| MKI67 | mRNA | 3.302564 | 1.67E-21 | 1.26E-18 |
| KIFC1 | mRNA | 3.369085 | 1.77E-21 | 1.28E-18 |
| CDCA8 | mRNA | 2.998954 | 2.37E-21 | 1.63E-18 |
| PLK1 | mRNA | 3.232258 | 2.43E-21 | 1.63E-18 |
| TROAP | mRNA | 3.724257 | 2.91E-21 | 1.87E-18 |
| SLC26A6 | mRNA | 2.317592 | 3.79E-21 | 2.35E-18 |
| CENPA | mRNA | 3.721365 | 4.93E-21 | 2.86E-18 |
| PRC1 | mRNA | 2.955653 | 4.94E-21 | 2.86E-18 |
| NDST3 | mRNA | -6.29848 | 7.44E-21 | 4.17E-18 |
| PTTG1 | mRNA | 3.28091 | 8.43E-21 | 4.46E-18 |
| CDCA3 | mRNA | 2.841465 | 8.47E-21 | 4.46E-18 |
| CDCA5 | mRNA | 3.1684 | 8.98E-21 | 4.59E-18 |
| BUB1 | mRNA | 3.438946 | 9.56E-21 | 4.74E-18 |
| CDC20 | mRNA | 3.802997 | 1.63E-20 | 7.87E-18 |
| DLGAP5 | mRNA | 3.46087 | 2.00E-20 | 9.40E-18 |
| FCN2 | mRNA | -7.26116 | 2.98E-20 | 1.33E-17 |
| CXCL14 | mRNA | -7.59163 | 3.00E-20 | 1.33E-17 |
| KIF18B | mRNA | 3.552883 | 3.13E-20 | 1.34E-17 |
| CCNB1 | mRNA | 2.851229 | 3.16E-20 | 1.34E-17 |
| INS-IGF2 | mRNA | -8.86025 | 3.55E-20 | 1.45E-17 |
| NUF2 | mRNA | 3.800245 | 3.64E-20 | 1.45E-17 |
| GPM6A | mRNA | -4.68126 | 3.67E-20 | 1.45E-17 |
| EBF2 | mRNA | 5.571521 | 4.31E-20 | 1.64E-17 |
| NTF3 | mRNA | -4.71646 | 4.35E-20 | 1.64E-17 |
| EZH2 | mRNA | 2.173138 | 4.68E-20 | 1.73E-17 |
| CSRNP1 | mRNA | -2.42053 | 5.23E-20 | 1.89E-17 |
| FOXM1 | mRNA | 3.387397 | 5.36E-20 | 1.90E-17 |
| RACGAP1 | mRNA | 2.247505 | 7.76E-20 | 2.70E-17 |
| HJURP | mRNA | 3.679114 | 9.12E-20 | 3.08E-17 |
| CDK1 | mRNA | 3.265256 | 9.22E-20 | 3.08E-17 |
| LIFR | mRNA | -3.54745 | 1.01E-19 | 3.30E-17 |
| AURKB | mRNA | 2.974017 | 1.13E-19 | 3.58E-17 |
| RD3L | mRNA | -5.50814 | 1.13E-19 | 3.58E-17 |
| CFP | mRNA | -4.48579 | 1.40E-19 | 4.33E-17 |
| CCNB2 | mRNA | 3.468961 | 1.45E-19 | 4.41E-17 |
| ASPM | mRNA | 3.727982 | 1.74E-19 | 5.22E-17 |
| HGF | mRNA | -3.56629 | 1.88E-19 | 5.55E-17 |
| CCBE1 | mRNA | -6.01402 | 1.95E-19 | 5.65E-17 |
| NEK2 | mRNA | 4.088222 | 2.56E-19 | 7.29E-17 |
| CENPL | mRNA | 2.215193 | 2.61E-19 | 7.31E-17 |
| ANLN | mRNA | 3.609437 | 2.84E-19 | 7.80E-17 |
| CHRM2 | mRNA | -6.51805 | 2.87E-19 | 7.80E-17 |
| ARHGEF39 | mRNA | 2.630379 | 2.94E-19 | 7.85E-17 |
| SPC25 | mRNA | 3.117398 | 3.52E-19 | 9.13E-17 |
| DBH | mRNA | -4.60607 | 5.31E-19 | 1.36E-16 |
| AMHR2 | mRNA | -5.3348 | 6.66E-19 | 1.62E-16 |
| OIT3 | mRNA | -3.7673 | 6.72E-19 | 1.62E-16 |
| PLAC8 | mRNA | -3.97253 | 6.72E-19 | 1.62E-16 |
| TOP2A | mRNA | 3.752125 | 6.85E-19 | 1.62E-16 |
| NCAPG | mRNA | 3.837388 | 6.90E-19 | 1.62E-16 |
| DACH1 | mRNA | -3.07478 | 6.91E-19 | 1.62E-16 |
| RRM2 | mRNA | 3.147854 | 7.78E-19 | 1.80E-16 |
| SKA1 | mRNA | 4.133025 | 8.11E-19 | 1.85E-16 |
| PAMR1 | mRNA | -2.76011 | 8.87E-19 | 2.00E-16 |
| IQGAP3 | mRNA | 3.905891 | 9.52E-19 | 2.12E-16 |
| GTSE1 | mRNA | 3.028006 | 1.12E-18 | 2.46E-16 |
| MELK | mRNA | 3.859293 | 1.35E-18 | 2.89E-16 |
| PLVAP | mRNA | 2.98978 | 1.44E-18 | 3.06E-16 |
| KIF18A | mRNA | 3.195805 | 1.53E-18 | 3.21E-16 |
| SPC24 | mRNA | 3.136908 | 1.65E-18 | 3.32E-16 |
| TRIP13 | mRNA | 2.776815 | 1.99E-18 | 3.97E-16 |
| WDR62 | mRNA | 2.181947 | 2.11E-18 | 4.13E-16 |
| KIF20A | mRNA | 3.540057 | 2.12E-18 | 4.13E-16 |
| TEX26 | mRNA | -5.54337 | 2.23E-18 | 4.31E-16 |
| PELI2 | mRNA | -2.41435 | 2.35E-18 | 4.49E-16 |
| MT1B | mRNA | -6.56116 | 2.52E-18 | 4.76E-16 |
| BUB1B | mRNA | 3.323353 | 3.21E-18 | 6.00E-16 |
| CXCL12 | mRNA | -3.88925 | 4.75E-18 | 8.78E-16 |
| NDC80 | mRNA | 2.962801 | 5.57E-18 | 1.01E-15 |
| BCO2 | mRNA | -4.66546 | 5.60E-18 | 1.01E-15 |
| NOX4 | mRNA | 3.025933 | 5.87E-18 | 1.05E-15 |
| NXF3 | mRNA | -4.99077 | 6.17E-18 | 1.09E-15 |
| EXO1 | mRNA | 3.567155 | 6.82E-18 | 1.20E-15 |
| COL15A1 | mRNA | 3.584389 | 7.89E-18 | 1.37E-15 |
| KIF23 | mRNA | 2.976146 | 9.19E-18 | 1.58E-15 |
| TCIM | mRNA | -2.94772 | 9.85E-18 | 1.68E-15 |
| SKA3 | mRNA | 3.377312 | 1.09E-17 | 1.83E-15 |
| CDC25C | mRNA | 4.199835 | 1.09E-17 | 1.83E-15 |
| CENPE | mRNA | 2.903452 | 1.11E-17 | 1.84E-15 |
| TRIB1 | mRNA | -2.24081 | 1.39E-17 | 2.28E-15 |
| SLC17A8 | mRNA | -4.73132 | 1.40E-17 | 2.28E-15 |
| GABRD | mRNA | 4.521155 | 1.47E-17 | 2.36E-15 |
| DIAPH3 | mRNA | 3.153204 | 1.49E-17 | 2.38E-15 |
| PVALB | mRNA | -5.18496 | 1.51E-17 | 2.39E-15 |
| MT1F | mRNA | -6.34704 | 1.55E-17 | 2.43E-15 |
| PITPNM3 | mRNA | -4.36734 | 1.59E-17 | 2.46E-15 |
| CENPM | mRNA | 3.214959 | 1.81E-17 | 2.78E-15 |
| CAMK4 | mRNA | -2.32399 | 1.86E-17 | 2.80E-15 |
| PTH1R | mRNA | -4.02857 | 1.86E-17 | 2.80E-15 |
| PHACTR3 | mRNA | -4.37908 | 1.87E-17 | 2.80E-15 |
| COL6A6 | mRNA | -4.32529 | 2.25E-17 | 3.34E-15 |
| PKMYT1 | mRNA | 2.940255 | 2.50E-17 | 3.64E-15 |
| ESM1 | mRNA | 4.719878 | 2.67E-17 | 3.87E-15 |
| SLCO1C1 | mRNA | 3.902873 | 3.70E-17 | 5.27E-15 |
| DTL | mRNA | 3.314196 | 3.90E-17 | 5.51E-15 |
| CKAP2L | mRNA | 3.329634 | 4.14E-17 | 5.80E-15 |
| MYBL2 | mRNA | 3.491554 | 5.16E-17 | 7.05E-15 |
| CDC45 | mRNA | 3.195124 | 5.29E-17 | 7.18E-15 |
| NUDT10 | mRNA | -4.94884 | 5.37E-17 | 7.23E-15 |
| E2F1 | mRNA | 3.557947 | 5.52E-17 | 7.38E-15 |
| RSPO3 | mRNA | -5.86075 | 6.18E-17 | 8.19E-15 |
| ARHGAP11A | mRNA | 2.325133 | 6.55E-17 | 8.62E-15 |
| KIF14 | mRNA | 3.062955 | 6.89E-17 | 8.97E-15 |
| CDH13 | mRNA | 2.793213 | 6.92E-17 | 8.97E-15 |
| GPR182 | mRNA | -4.89615 | 7.16E-17 | 9.22E-15 |
| ASF1B | mRNA | 2.534312 | 7.35E-17 | 9.39E-15 |
| FLVCR1 | mRNA | 2.080723 | 7.63E-17 | 9.68E-15 |
| TRAIP | mRNA | 2.516144 | 7.73E-17 | 9.73E-15 |
| CEP55 | mRNA | 2.549922 | 8.08E-17 | 1.01E-14 |
| ERCC6L | mRNA | 2.93003 | 8.90E-17 | 1.10E-14 |
| MXD3 | mRNA | 2.236583 | 8.94E-17 | 1.10E-14 |
| ORC1 | mRNA | 2.945993 | 1.00E-16 | 1.21E-14 |
| NDUFA4L2 | mRNA | 2.40705 | 1.07E-16 | 1.29E-14 |
| IGFBP3 | mRNA | -2.57247 | 1.10E-16 | 1.31E-14 |
| UHRF1 | mRNA | 3.061576 | 1.20E-16 | 1.42E-14 |
| HHIP | mRNA | -6.98444 | 1.31E-16 | 1.55E-14 |
| CDC6 | mRNA | 2.966648 | 1.59E-16 | 1.86E-14 |
| TICRR | mRNA | 3.303864 | 1.81E-16 | 2.09E-14 |
| KCNN2 | mRNA | -5.00218 | 1.88E-16 | 2.16E-14 |
| CENPI | mRNA | 2.964616 | 2.37E-16 | 2.67E-14 |
| EME1 | mRNA | 2.71946 | 2.42E-16 | 2.71E-14 |
| CD34 | mRNA | 2.238881 | 2.44E-16 | 2.72E-14 |
| MT1H | mRNA | -8.67459 | 2.99E-16 | 3.31E-14 |
| ALKAL1 | mRNA | -4.60857 | 3.39E-16 | 3.73E-14 |
| SGO1 | mRNA | 2.727712 | 3.64E-16 | 3.97E-14 |
| GABRQ | mRNA | 4.574405 | 3.77E-16 | 4.07E-14 |
| CYR61 | mRNA | -2.4915 | 3.81E-16 | 4.08E-14 |
| RND3 | mRNA | -2.74907 | 3.86E-16 | 4.12E-14 |
| LYVE1 | mRNA | -3.8334 | 3.93E-16 | 4.17E-14 |
| EPHA2 | mRNA | -2.29328 | 4.23E-16 | 4.45E-14 |
| LRAT | mRNA | -3.77953 | 4.41E-16 | 4.62E-14 |
| OLFML2B | mRNA | 2.950198 | 4.76E-16 | 4.95E-14 |
| ANGPTL6 | mRNA | -3.52222 | 4.97E-16 | 5.08E-14 |
| MAGEA1 | mRNA | 8.176278 | 5.73E-16 | 5.79E-14 |
| HMMR | mRNA | 3.263094 | 5.91E-16 | 5.93E-14 |
| DEPDC1 | mRNA | 3.930616 | 6.13E-16 | 6.08E-14 |
| OLFML3 | mRNA | -2.83721 | 6.49E-16 | 6.41E-14 |
| APLN | mRNA | 3.940717 | 7.14E-16 | 7.01E-14 |
| HOXA13 | mRNA | 7.189531 | 7.30E-16 | 7.13E-14 |
| FANCI | mRNA | 2.003472 | 7.78E-16 | 7.55E-14 |
| E2F2 | mRNA | 2.635644 | 7.84E-16 | 7.57E-14 |
| PBK | mRNA | 3.746863 | 8.02E-16 | 7.70E-14 |
| CPEB3 | mRNA | -2.37875 | 8.12E-16 | 7.71E-14 |
| TCF19 | mRNA | 2.461106 | 8.45E-16 | 7.98E-14 |
| RECQL4 | mRNA | 2.03141 | 8.66E-16 | 8.13E-14 |
| STIL | mRNA | 2.201887 | 8.84E-16 | 8.23E-14 |
| ZIC5 | mRNA | 6.722673 | 1.02E-15 | 9.39E-14 |
| SLC5A1 | mRNA | -6.08816 | 1.03E-15 | 9.42E-14 |
| TERT | mRNA | 5.841509 | 1.04E-15 | 9.50E-14 |
| E2F8 | mRNA | 3.718585 | 1.06E-15 | 9.64E-14 |
| HIGD1B | mRNA | 3.594104 | 1.14E-15 | 1.02E-13 |
| PMP2 | mRNA | -4.44927 | 1.16E-15 | 1.03E-13 |
| NCAPH | mRNA | 2.667041 | 1.20E-15 | 1.07E-13 |
| DNASE1L3 | mRNA | -3.99187 | 1.33E-15 | 1.16E-13 |
| TTK | mRNA | 3.464586 | 1.33E-15 | 1.16E-13 |
| LRRN3 | mRNA | -3.58552 | 1.58E-15 | 1.37E-13 |
| SEMA5B | mRNA | 2.271958 | 1.61E-15 | 1.38E-13 |
| HOXD9 | mRNA | 5.249995 | 1.67E-15 | 1.43E-13 |
| ITLN1 | mRNA | -5.75208 | 1.70E-15 | 1.44E-13 |
| BMP5 | mRNA | -4.86341 | 1.76E-15 | 1.47E-13 |
| AURKA | mRNA | 2.409591 | 1.85E-15 | 1.54E-13 |
| SIGLEC7 | mRNA | -2.47831 | 1.93E-15 | 1.59E-13 |
| EFNB3 | mRNA | -3.25468 | 1.99E-15 | 1.62E-13 |
| CCNA2 | mRNA | 3.287827 | 1.99E-15 | 1.62E-13 |
| MT2A | mRNA | -4.53803 | 1.99E-15 | 1.62E-13 |
| FBXO43 | mRNA | 3.619652 | 2.00E-15 | 1.62E-13 |
| MT1G | mRNA | -7.52855 | 2.03E-15 | 1.63E-13 |
| TUBE1 | mRNA | -2.12085 | 2.11E-15 | 1.69E-13 |
| LILRA2 | mRNA | -2.14047 | 2.16E-15 | 1.71E-13 |
| HAMP | mRNA | -8.54681 | 2.25E-15 | 1.77E-13 |
| CBFA2T3 | mRNA | -2.38377 | 2.27E-15 | 1.78E-13 |
| PCDH9 | mRNA | -3.7021 | 2.28E-15 | 1.78E-13 |
| SIGLEC11 | mRNA | -2.85805 | 2.40E-15 | 1.87E-13 |
| SGO2 | mRNA | 2.249495 | 2.64E-15 | 2.03E-13 |
| CDT1 | mRNA | 3.159081 | 2.80E-15 | 2.13E-13 |
| PRR11 | mRNA | 2.102853 | 3.12E-15 | 2.36E-13 |
| ECT2 | mRNA | 2.193055 | 3.16E-15 | 2.37E-13 |
| ZIC2 | mRNA | 6.127838 | 3.21E-15 | 2.40E-13 |
| MND1 | mRNA | 3.252644 | 3.36E-15 | 2.50E-13 |
| MT1M | mRNA | -6.49738 | 3.44E-15 | 2.55E-13 |
| SFRP5 | mRNA | -7.21095 | 3.47E-15 | 2.55E-13 |
| KDM8 | mRNA | -3.10473 | 3.69E-15 | 2.70E-13 |
| COX4I2 | mRNA | 3.192017 | 3.73E-15 | 2.72E-13 |
| S100A12 | mRNA | -3.28945 | 4.07E-15 | 2.94E-13 |
| RAD54L | mRNA | 3.192692 | 4.22E-15 | 3.03E-13 |
| NGFR | mRNA | -3.4589 | 4.24E-15 | 3.03E-13 |
| ADRA2B | mRNA | -2.30393 | 4.39E-15 | 3.13E-13 |
| ACADS | mRNA | -2.01732 | 5.05E-15 | 3.58E-13 |
| ZFP36 | mRNA | -2.11508 | 5.10E-15 | 3.60E-13 |
| CXorf36 | mRNA | 2.432358 | 5.52E-15 | 3.88E-13 |
| TBXA2R | mRNA | -2.1585 | 6.24E-15 | 4.35E-13 |
| PRIMA1 | mRNA | -5.02551 | 6.27E-15 | 4.36E-13 |
| CELSR3 | mRNA | 3.058729 | 6.79E-15 | 4.68E-13 |
| LRRC55 | mRNA | -3.62368 | 6.87E-15 | 4.72E-13 |
| S100A8 | mRNA | -2.91071 | 7.46E-15 | 5.04E-13 |
| RCAN1 | mRNA | -2.5606 | 7.59E-15 | 5.11E-13 |
| CYP26A1 | mRNA | -5.40033 | 8.32E-15 | 5.50E-13 |
| SRPX | mRNA | -3.7735 | 8.47E-15 | 5.58E-13 |
| HMGCLL1 | mRNA | -4.09678 | 8.81E-15 | 5.69E-13 |
| HIST1H3J | mRNA | 3.563306 | 8.82E-15 | 5.69E-13 |
| MCM10 | mRNA | 3.013638 | 8.85E-15 | 5.69E-13 |
| SOCS2 | mRNA | -2.72169 | 9.31E-15 | 5.97E-13 |
| RASD2 | mRNA | 2.871117 | 9.90E-15 | 6.31E-13 |
| MT1X | mRNA | -5.16401 | 9.92E-15 | 6.31E-13 |
| C5orf34 | mRNA | 2.274985 | 9.98E-15 | 6.33E-13 |
| WNT3A | mRNA | 6.755462 | 1.05E-14 | 6.58E-13 |
| PARPBP | mRNA | 2.43501 | 1.05E-14 | 6.59E-13 |
| TMEM26 | mRNA | -2.5783 | 1.15E-14 | 7.14E-13 |
| FAM83D | mRNA | 2.62192 | 1.39E-14 | 8.60E-13 |
| SLITRK6 | mRNA | -4.891 | 1.45E-14 | 8.96E-13 |
| TH | mRNA | -4.19202 | 1.46E-14 | 8.98E-13 |
| SHCBP1 | mRNA | 3.007449 | 1.53E-14 | 9.35E-13 |
| PRAM1 | mRNA | -2.25815 | 1.56E-14 | 9.50E-13 |
| CLEC12A | mRNA | -2.773 | 1.82E-14 | 1.10E-12 |
| PZP | mRNA | -6.13076 | 1.88E-14 | 1.12E-12 |
| SMPD3 | mRNA | -2.93608 | 1.92E-14 | 1.15E-12 |
| ZWINT | mRNA | 2.226346 | 1.93E-14 | 1.15E-12 |
| ADGRA1 | mRNA | -4.89939 | 2.01E-14 | 1.19E-12 |
| LTK | mRNA | -3.15089 | 2.16E-14 | 1.26E-12 |
| CAP2 | mRNA | 2.100842 | 2.30E-14 | 1.34E-12 |
| RFPL1 | mRNA | -4.26471 | 2.60E-14 | 1.50E-12 |
| POLQ | mRNA | 2.683741 | 2.66E-14 | 1.54E-12 |
| RIPOR3 | mRNA | -3.4839 | 2.69E-14 | 1.54E-12 |
| P3H2 | mRNA | -2.15367 | 2.71E-14 | 1.55E-12 |
| SFRP1 | mRNA | -4.2554 | 2.78E-14 | 1.58E-12 |
| CDCA2 | mRNA | 2.940675 | 2.88E-14 | 1.62E-12 |
| PRKAR2B | mRNA | -3.08511 | 3.16E-14 | 1.76E-12 |
| MS4A6A | mRNA | -2.17885 | 3.20E-14 | 1.78E-12 |
| EHD3 | mRNA | -2.41434 | 3.54E-14 | 1.97E-12 |
| CALN1 | mRNA | -4.78804 | 3.66E-14 | 2.02E-12 |
| THBS4 | mRNA | 4.637309 | 4.22E-14 | 2.32E-12 |
| KIF19 | mRNA | -3.44774 | 4.88E-14 | 2.65E-12 |
| KIF15 | mRNA | 2.797217 | 4.93E-14 | 2.67E-12 |
| PCLAF | mRNA | 2.059821 | 5.04E-14 | 2.71E-12 |
| NPY1R | mRNA | -3.03875 | 6.09E-14 | 3.24E-12 |
| TMEM154 | mRNA | -2.99317 | 6.79E-14 | 3.55E-12 |
| FGF23 | mRNA | -6.08404 | 7.52E-14 | 3.88E-12 |
| MT1E | mRNA | -5.77372 | 7.55E-14 | 3.88E-12 |
| C1R | mRNA | -2.0694 | 8.47E-14 | 4.27E-12 |
| FOSB | mRNA | -4.57456 | 8.87E-14 | 4.41E-12 |
| ST8SIA6 | mRNA | -3.56276 | 9.54E-14 | 4.72E-12 |
| CDKN2C | mRNA | 2.13666 | 9.62E-14 | 4.75E-12 |
| LCAT | mRNA | -3.54735 | 1.07E-13 | 5.24E-12 |
| CETP | mRNA | -3.27133 | 1.12E-13 | 5.45E-12 |
| XRCC2 | mRNA | 2.607268 | 1.19E-13 | 5.76E-12 |
| PLXDC1 | mRNA | 2.58263 | 1.26E-13 | 6.06E-12 |
| GINS1 | mRNA | 2.381797 | 1.36E-13 | 6.47E-12 |
| HBB | mRNA | -2.34927 | 1.47E-13 | 6.97E-12 |
| SYT10 | mRNA | -4.75581 | 1.48E-13 | 6.99E-12 |
| FIBCD1 | mRNA | 5.160885 | 1.56E-13 | 7.33E-12 |
| ITGA9 | mRNA | -2.20926 | 1.62E-13 | 7.55E-12 |
| C14orf180 | mRNA | -5.99653 | 1.68E-13 | 7.80E-12 |
| ADGRB3 | mRNA | -3.68056 | 1.84E-13 | 8.54E-12 |
| FPR2 | mRNA | -3.45255 | 1.88E-13 | 8.70E-12 |
| GABRP | mRNA | -5.06063 | 1.91E-13 | 8.79E-12 |
| EGR1 | mRNA | -3.07809 | 1.96E-13 | 8.99E-12 |
| RNF165 | mRNA | -3.15552 | 2.18E-13 | 9.89E-12 |
| GSTZ1 | mRNA | -2.42364 | 2.21E-13 | 1.00E-11 |
| LILRB5 | mRNA | -2.50679 | 2.30E-13 | 1.04E-11 |
| CPLX2 | mRNA | 8.593066 | 2.50E-13 | 1.11E-11 |
| NT5DC2 | mRNA | 2.083566 | 2.51E-13 | 1.11E-11 |
| MCM2 | mRNA | 2.220718 | 2.72E-13 | 1.19E-11 |
| GNA14 | mRNA | -2.40466 | 2.80E-13 | 1.22E-11 |
| CXCR1 | mRNA | -2.9586 | 3.07E-13 | 1.32E-11 |
| TK1 | mRNA | 2.02259 | 3.13E-13 | 1.34E-11 |
| CDKN2A | mRNA | 3.481834 | 3.22E-13 | 1.38E-11 |
| LY6E | mRNA | -3.95569 | 3.60E-13 | 1.54E-11 |
| EDIL3 | mRNA | 2.612635 | 3.74E-13 | 1.59E-11 |
| SLC6A19 | mRNA | -6.22475 | 3.80E-13 | 1.61E-11 |
| PLIN2 | mRNA | -2.14629 | 3.88E-13 | 1.63E-11 |
| TCTEX1D1 | mRNA | -3.74071 | 4.35E-13 | 1.81E-11 |
| IL1B | mRNA | -2.20309 | 4.36E-13 | 1.81E-11 |
| LYPD2 | mRNA | -4.68453 | 4.37E-13 | 1.81E-11 |
| TEDC2 | mRNA | 2.549018 | 4.40E-13 | 1.82E-11 |
| ID1 | mRNA | -2.48171 | 4.43E-13 | 1.82E-11 |
| CD4 | mRNA | -2.09829 | 4.46E-13 | 1.83E-11 |
| OLFML2A | mRNA | 2.322373 | 5.06E-13 | 2.06E-11 |
| TCF21 | mRNA | -2.55865 | 5.33E-13 | 2.16E-11 |
| DIRAS3 | mRNA | -4.0203 | 5.55E-13 | 2.24E-11 |
| COX7B2 | mRNA | 9.467536 | 5.59E-13 | 2.25E-11 |
| NAA11 | mRNA | 6.130601 | 5.85E-13 | 2.34E-11 |
| FAM111B | mRNA | 3.197848 | 6.04E-13 | 2.41E-11 |
| DCN | mRNA | -4.17074 | 6.07E-13 | 2.42E-11 |
| CROCC2 | mRNA | -3.54602 | 6.23E-13 | 2.47E-11 |
| STARD5 | mRNA | -2.01201 | 6.34E-13 | 2.51E-11 |
| FOS | mRNA | -3.64213 | 6.48E-13 | 2.55E-11 |
| CENPU | mRNA | 2.182429 | 6.59E-13 | 2.57E-11 |
| E2F7 | mRNA | 3.286451 | 6.76E-13 | 2.62E-11 |
| FAM180A | mRNA | -4.61606 | 6.79E-13 | 2.62E-11 |
| RXFP1 | mRNA | -2.13074 | 6.79E-13 | 2.62E-11 |
| FATE1 | mRNA | 4.250611 | 6.86E-13 | 2.63E-11 |
| RASL12 | mRNA | 2.263056 | 7.07E-13 | 2.70E-11 |
| CCL23 | mRNA | -3.317 | 7.12E-13 | 2.71E-11 |
| ORC6 | mRNA | 2.490168 | 7.23E-13 | 2.74E-11 |
| THY1 | mRNA | 2.690331 | 7.26E-13 | 2.74E-11 |
| TMEM27 | mRNA | -3.81033 | 7.88E-13 | 2.95E-11 |
| ESR1 | mRNA | -3.84704 | 8.12E-13 | 3.03E-11 |
| PROM2 | mRNA | -3.11079 | 8.26E-13 | 3.07E-11 |
| TIMD4 | mRNA | -4.77164 | 8.28E-13 | 3.07E-11 |
| PCSK2 | mRNA | -4.2542 | 8.34E-13 | 3.09E-11 |
| MAPT | mRNA | 2.905977 | 8.36E-13 | 3.09E-11 |
| CD163 | mRNA | -2.30202 | 8.40E-13 | 3.10E-11 |
| KAZN | mRNA | -2.603 | 8.49E-13 | 3.11E-11 |
| RASGEF1B | mRNA | -2.22981 | 8.50E-13 | 3.11E-11 |
| EVX1 | mRNA | 5.366062 | 8.81E-13 | 3.22E-11 |
| SLC28A3 | mRNA | -3.79 | 1.02E-12 | 3.65E-11 |
| IL13RA2 | mRNA | -4.11977 | 1.12E-12 | 3.99E-11 |
| LILRA5 | mRNA | -2.538 | 1.13E-12 | 4.02E-11 |
| NEIL3 | mRNA | 3.806584 | 1.21E-12 | 4.24E-11 |
| KCNJ16 | mRNA | -5.10522 | 1.31E-12 | 4.57E-11 |
| CCL14 | mRNA | -2.44662 | 1.37E-12 | 4.73E-11 |
| HK3 | mRNA | -2.26905 | 1.37E-12 | 4.74E-11 |
| RNF152 | mRNA | -2.09109 | 1.38E-12 | 4.77E-11 |
| C21orf62 | mRNA | -3.84849 | 1.40E-12 | 4.83E-11 |
| CENPK | mRNA | 2.409436 | 1.42E-12 | 4.87E-11 |
| EBF1 | mRNA | 2.15136 | 1.44E-12 | 4.94E-11 |
| INMT | mRNA | -3.69244 | 1.53E-12 | 5.21E-11 |
| FPR1 | mRNA | -2.57429 | 1.54E-12 | 5.25E-11 |
| WDR66 | mRNA | -2.47353 | 1.58E-12 | 5.37E-11 |
| AADAT | mRNA | -3.56085 | 1.59E-12 | 5.37E-11 |
| FAM83F | mRNA | -4.59595 | 1.61E-12 | 5.43E-11 |
| JCHAIN | mRNA | -4.75227 | 1.62E-12 | 5.46E-11 |
| THBS1 | mRNA | -2.50986 | 1.74E-12 | 5.80E-11 |
| MAD2L1 | mRNA | 2.021163 | 1.81E-12 | 6.01E-11 |
| TNFRSF4 | mRNA | 2.590986 | 1.81E-12 | 6.01E-11 |
| FCGR2B | mRNA | -3.75883 | 1.87E-12 | 6.19E-11 |
| CFTR | mRNA | -5.93999 | 1.89E-12 | 6.25E-11 |
| IL1RAP | mRNA | -2.56189 | 1.94E-12 | 6.35E-11 |
| TACSTD2 | mRNA | -3.54248 | 1.96E-12 | 6.41E-11 |
| PAEP | mRNA | 8.311152 | 2.02E-12 | 6.60E-11 |
| EGFL6 | mRNA | 3.951638 | 2.26E-12 | 7.31E-11 |
| GRHL2 | mRNA | -4.61624 | 2.31E-12 | 7.45E-11 |
| HOXD8 | mRNA | 3.548835 | 2.34E-12 | 7.51E-11 |
| LRRC4 | mRNA | -2.27594 | 2.34E-12 | 7.51E-11 |
| FAM57B | mRNA | 3.526782 | 2.37E-12 | 7.59E-11 |
| STEAP3 | mRNA | -2.3215 | 2.37E-12 | 7.59E-11 |
| ROBO1 | mRNA | 2.469852 | 2.45E-12 | 7.81E-11 |
| SOCS3 | mRNA | -2.5593 | 2.80E-12 | 8.88E-11 |
| OTX1 | mRNA | 3.900123 | 2.83E-12 | 8.93E-11 |
| EGR2 | mRNA | -3.0592 | 3.01E-12 | 9.45E-11 |
| CSPG5 | mRNA | 2.176709 | 3.10E-12 | 9.70E-11 |
| DCAF8L2 | mRNA | 7.169946 | 3.11E-12 | 9.72E-11 |
| COL25A1 | mRNA | -3.90529 | 3.28E-12 | 1.02E-10 |
| CHST4 | mRNA | -6.36963 | 3.31E-12 | 1.03E-10 |
| CD226 | mRNA | -2.19433 | 3.48E-12 | 1.08E-10 |
| CXCR2 | mRNA | -2.85752 | 3.53E-12 | 1.09E-10 |
| HS3ST3A1 | mRNA | -2.4377 | 3.94E-12 | 1.20E-10 |
| FAM72D | mRNA | 3.215307 | 3.99E-12 | 1.21E-10 |
| CBX2 | mRNA | 2.247432 | 4.08E-12 | 1.23E-10 |
| BEND4 | mRNA | -3.4054 | 4.39E-12 | 1.32E-10 |
| IL33 | mRNA | -2.55628 | 4.41E-12 | 1.32E-10 |
| DUSP5 | mRNA | -2.10601 | 4.45E-12 | 1.33E-10 |
| CD5L | mRNA | -5.55374 | 5.08E-12 | 1.50E-10 |
| MUC6 | mRNA | -5.97602 | 5.09E-12 | 1.50E-10 |
| PHLDA1 | mRNA | -2.76294 | 5.17E-12 | 1.52E-10 |
| OR51E1 | mRNA | 3.266357 | 5.18E-12 | 1.52E-10 |
| TRPV6 | mRNA | -4.94082 | 5.28E-12 | 1.54E-10 |
| ADGRE1 | mRNA | -2.63947 | 5.33E-12 | 1.55E-10 |
| FFAR2 | mRNA | -2.19932 | 5.59E-12 | 1.61E-10 |
| CLSPN | mRNA | 2.846999 | 5.79E-12 | 1.67E-10 |
| PDZRN4 | mRNA | -4.18466 | 5.99E-12 | 1.72E-10 |
| SIX1 | mRNA | 4.527215 | 6.01E-12 | 1.72E-10 |
| ARHGAP10 | mRNA | -2.02511 | 6.08E-12 | 1.73E-10 |
| SELP | mRNA | -2.90895 | 6.16E-12 | 1.75E-10 |
| NOTCH3 | mRNA | 2.143233 | 6.33E-12 | 1.80E-10 |
| GHR | mRNA | -2.77698 | 6.36E-12 | 1.80E-10 |
| SLC7A8 | mRNA | -2.12243 | 6.87E-12 | 1.91E-10 |
| ADGRG7 | mRNA | -3.61968 | 6.89E-12 | 1.92E-10 |
| NCAM1 | mRNA | -3.4533 | 7.01E-12 | 1.94E-10 |
| RAD51AP1 | mRNA | 2.084684 | 7.08E-12 | 1.96E-10 |
| SSX1 | mRNA | 9.35176 | 7.19E-12 | 1.99E-10 |
| PLP1 | mRNA | -4.37099 | 7.47E-12 | 2.05E-10 |
| FAM163B | mRNA | -4.72299 | 7.68E-12 | 2.10E-10 |
| HBA2 | mRNA | -2.41475 | 7.98E-12 | 2.17E-10 |
| MTFR2 | mRNA | 2.812258 | 8.23E-12 | 2.22E-10 |
| SIX2 | mRNA | 4.602237 | 8.24E-12 | 2.22E-10 |
| ST6GAL2 | mRNA | -4.06571 | 8.33E-12 | 2.24E-10 |
| HASPIN | mRNA | 2.440296 | 8.40E-12 | 2.25E-10 |
| PXDNL | mRNA | 2.891685 | 8.40E-12 | 2.25E-10 |
| RAB25 | mRNA | -5.04702 | 8.87E-12 | 2.36E-10 |
| GJC1 | mRNA | 2.198226 | 8.94E-12 | 2.38E-10 |
| CSPG4 | mRNA | 2.356937 | 9.22E-12 | 2.44E-10 |
| CCNE1 | mRNA | 2.560708 | 9.23E-12 | 2.44E-10 |
| VCX | mRNA | 4.921052 | 9.59E-12 | 2.52E-10 |
| PPBP | mRNA | -3.19358 | 9.66E-12 | 2.53E-10 |
| MESP2 | mRNA | 2.923897 | 9.95E-12 | 2.59E-10 |
| WNT2 | mRNA | -4.5156 | 1.02E-11 | 2.63E-10 |
| LY6H | mRNA | 3.764198 | 1.02E-11 | 2.64E-10 |
| AC007906.2 | mRNA | -2.67729 | 1.03E-11 | 2.65E-10 |
| APOF | mRNA | -4.60741 | 1.04E-11 | 2.67E-10 |
| TSLP | mRNA | -3.4191 | 1.08E-11 | 2.76E-10 |
| PADI4 | mRNA | -2.88074 | 1.09E-11 | 2.77E-10 |
| DPT | mRNA | -4.86648 | 1.11E-11 | 2.81E-10 |
| MARCH4 | mRNA | 3.736073 | 1.19E-11 | 3.01E-10 |
| RNF125 | mRNA | -2.16417 | 1.22E-11 | 3.08E-10 |
| TEX19 | mRNA | 4.304153 | 1.23E-11 | 3.09E-10 |
| CD1D | mRNA | -2.55489 | 1.23E-11 | 3.09E-10 |
| TRAPPC3L | mRNA | -2.77146 | 1.24E-11 | 3.11E-10 |
| IL10 | mRNA | -2.13399 | 1.29E-11 | 3.23E-10 |
| CILP | mRNA | -3.19783 | 1.30E-11 | 3.24E-10 |
| TCHH | mRNA | -2.63803 | 1.33E-11 | 3.31E-10 |
| IBSP | mRNA | 5.143905 | 1.42E-11 | 3.51E-10 |
| MAN1C1 | mRNA | -2.41715 | 1.47E-11 | 3.61E-10 |
| ATP13A4 | mRNA | -3.75759 | 1.53E-11 | 3.74E-10 |
| RAB3B | mRNA | 3.862997 | 1.77E-11 | 4.29E-10 |
| BCAN | mRNA | 3.897211 | 1.78E-11 | 4.30E-10 |
| WDR17 | mRNA | -2.2698 | 1.79E-11 | 4.31E-10 |
| MT1HL1 | mRNA | -4.0199 | 1.83E-11 | 4.40E-10 |
| IGFALS | mRNA | -5.11115 | 1.88E-11 | 4.49E-10 |
| P2RY13 | mRNA | -2.18257 | 1.89E-11 | 4.52E-10 |
| IDO2 | mRNA | -4.39702 | 1.93E-11 | 4.58E-10 |
| MDK | mRNA | 3.222137 | 1.97E-11 | 4.66E-10 |
| ACSM3 | mRNA | -2.69014 | 1.99E-11 | 4.69E-10 |
| KBTBD11 | mRNA | -3.63504 | 2.01E-11 | 4.74E-10 |
| CNDP1 | mRNA | -6.79782 | 2.03E-11 | 4.76E-10 |
| TRPV4 | mRNA | -3.22512 | 2.13E-11 | 4.99E-10 |
| PTGS2 | mRNA | -3.34773 | 2.15E-11 | 5.02E-10 |
| PIF1 | mRNA | 2.088498 | 2.17E-11 | 5.05E-10 |
| P2RY12 | mRNA | -2.74854 | 2.18E-11 | 5.08E-10 |
| CD244 | mRNA | -2.38862 | 2.20E-11 | 5.11E-10 |
| NNMT | mRNA | -3.7899 | 2.23E-11 | 5.18E-10 |
| TFPI2 | mRNA | -3.12129 | 2.28E-11 | 5.29E-10 |
| LRRC4C | mRNA | -3.04584 | 2.38E-11 | 5.47E-10 |
| NAT2 | mRNA | -4.79425 | 2.43E-11 | 5.56E-10 |
| MAGEB17 | mRNA | 5.178292 | 2.47E-11 | 5.64E-10 |
| CHAF1B | mRNA | 2.036659 | 2.51E-11 | 5.74E-10 |
| HIST1H4H | mRNA | 2.553858 | 2.78E-11 | 6.30E-10 |
| CD200 | mRNA | 2.151569 | 2.99E-11 | 6.76E-10 |
| ADRA1A | mRNA | -4.49996 | 3.02E-11 | 6.81E-10 |
| MXRA5 | mRNA | -2.30142 | 3.02E-11 | 6.81E-10 |
| NXPH4 | mRNA | 3.706349 | 3.04E-11 | 6.84E-10 |
| TNXB | mRNA | -2.52635 | 3.09E-11 | 6.95E-10 |
| SPSB4 | mRNA | -4.03749 | 3.12E-11 | 6.98E-10 |
| MCC | mRNA | -2.18974 | 3.17E-11 | 7.08E-10 |
| GNAO1 | mRNA | -3.69024 | 3.21E-11 | 7.15E-10 |
| EGR3 | mRNA | -2.35623 | 3.24E-11 | 7.20E-10 |
| CYP2C8 | mRNA | -3.79744 | 3.26E-11 | 7.24E-10 |
| CXCL2 | mRNA | -3.07018 | 3.31E-11 | 7.31E-10 |
| TRPC6 | mRNA | 2.050874 | 3.36E-11 | 7.41E-10 |
| DHODH | mRNA | -2.08109 | 3.54E-11 | 7.72E-10 |
| HAND2 | mRNA | -4.04081 | 3.70E-11 | 8.03E-10 |
| VSIG4 | mRNA | -2.25221 | 3.75E-11 | 8.13E-10 |
| ANXA8 | mRNA | -3.91577 | 3.84E-11 | 8.30E-10 |
| KLRF1 | mRNA | -2.1047 | 3.88E-11 | 8.37E-10 |
| ERFE | mRNA | 2.277653 | 3.93E-11 | 8.48E-10 |
| CYP1A2 | mRNA | -7.77876 | 3.99E-11 | 8.60E-10 |
| PBLD | mRNA | -2.35066 | 4.10E-11 | 8.81E-10 |
| FCGR3B | mRNA | -2.42345 | 4.14E-11 | 8.87E-10 |
| SRD5A2 | mRNA | -3.54747 | 4.54E-11 | 9.62E-10 |
| STEAP4 | mRNA | -2.48625 | 4.81E-11 | 1.01E-09 |
| HOXD3 | mRNA | 3.506139 | 4.87E-11 | 1.02E-09 |
| FREM2 | mRNA | -6.02632 | 4.99E-11 | 1.04E-09 |
| MYOM2 | mRNA | -3.00527 | 5.14E-11 | 1.07E-09 |
| LIN28B | mRNA | 7.398702 | 5.27E-11 | 1.09E-09 |
| PROK2 | mRNA | -3.42965 | 5.44E-11 | 1.12E-09 |
| HOXA11 | mRNA | 4.749778 | 5.50E-11 | 1.13E-09 |
| MYO18B | mRNA | 5.382901 | 5.93E-11 | 1.21E-09 |
| RANBP3L | mRNA | -3.74603 | 6.08E-11 | 1.23E-09 |
| CR1 | mRNA | -2.80559 | 6.48E-11 | 1.30E-09 |
| SLC7A11 | mRNA | 3.967713 | 7.29E-11 | 1.44E-09 |
| MRC1 | mRNA | -2.1783 | 7.33E-11 | 1.45E-09 |
| FAM13A | mRNA | -2.64591 | 7.42E-11 | 1.46E-09 |
| MAGEB2 | mRNA | 7.522055 | 7.70E-11 | 1.51E-09 |
| NETO2 | mRNA | 2.139007 | 7.80E-11 | 1.53E-09 |
| CYS1 | mRNA | -4.03478 | 7.95E-11 | 1.55E-09 |
| IL18R1 | mRNA | -2.04641 | 8.28E-11 | 1.61E-09 |
| GNAZ | mRNA | 2.820887 | 8.45E-11 | 1.64E-09 |
| PAK5 | mRNA | -3.80043 | 8.45E-11 | 1.64E-09 |
| ASB16 | mRNA | 2.02357 | 8.62E-11 | 1.66E-09 |
| MFSD2A | mRNA | -4.78219 | 8.62E-11 | 1.66E-09 |
| GRIN2B | mRNA | -4.32507 | 8.71E-11 | 1.67E-09 |
| SHBG | mRNA | -3.6775 | 8.96E-11 | 1.72E-09 |
| BRINP2 | mRNA | -3.46551 | 9.24E-11 | 1.77E-09 |
| HOXD1 | mRNA | 3.814891 | 9.62E-11 | 1.83E-09 |
| SERPINE1 | mRNA | -3.02891 | 9.84E-11 | 1.87E-09 |
| KCNK17 | mRNA | -2.7779 | 9.90E-11 | 1.88E-09 |
| PROM1 | mRNA | -5.08925 | 1.00E-10 | 1.90E-09 |
| CCL3 | mRNA | -2.13367 | 1.02E-10 | 1.94E-09 |
| LRRN1 | mRNA | -3.8884 | 1.07E-10 | 2.01E-09 |
| SLC25A47 | mRNA | -5.73634 | 1.09E-10 | 2.04E-09 |
| TBX20 | mRNA | -3.60152 | 1.13E-10 | 2.11E-09 |
| ASPA | mRNA | -2.65963 | 1.14E-10 | 2.13E-09 |
| C7 | mRNA | -3.91125 | 1.14E-10 | 2.13E-09 |
| CDH19 | mRNA | -4.14389 | 1.20E-10 | 2.24E-09 |
| RFPL4B | mRNA | 6.670654 | 1.21E-10 | 2.25E-09 |
| STC2 | mRNA | 3.024113 | 1.21E-10 | 2.25E-09 |
| TNFRSF11B | mRNA | -2.2571 | 1.25E-10 | 2.32E-09 |
| C1QTNF1 | mRNA | -2.71843 | 1.26E-10 | 2.34E-09 |
| BGN | mRNA | -2.20251 | 1.28E-10 | 2.36E-09 |
| ANKRD55 | mRNA | -3.11324 | 1.46E-10 | 2.67E-09 |
| MRO | mRNA | -2.57057 | 1.55E-10 | 2.80E-09 |
| GLP2R | mRNA | -3.66 | 1.57E-10 | 2.82E-09 |
| SSX5 | mRNA | 4.527703 | 1.61E-10 | 2.89E-09 |
| LHX2 | mRNA | -2.8513 | 1.62E-10 | 2.90E-09 |
| TENM3 | mRNA | -3.8357 | 1.70E-10 | 3.03E-09 |
| OXT | mRNA | -5.07038 | 1.72E-10 | 3.05E-09 |
| PLPP4 | mRNA | -4.17512 | 1.81E-10 | 3.19E-09 |
| ISX | mRNA | 6.12225 | 1.82E-10 | 3.21E-09 |
| PRSS22 | mRNA | -4.13044 | 1.83E-10 | 3.22E-09 |
| LPL | mRNA | 2.621045 | 1.84E-10 | 3.24E-09 |
| DDX53 | mRNA | 7.066537 | 1.85E-10 | 3.25E-09 |
| SIRPB1 | mRNA | -2.01279 | 1.89E-10 | 3.30E-09 |
| SKAP1 | mRNA | -2.33815 | 1.90E-10 | 3.32E-09 |
| DLGAP2 | mRNA | -3.78225 | 2.05E-10 | 3.56E-09 |
| HOXD4 | mRNA | 4.10874 | 2.06E-10 | 3.57E-09 |
| PTGIS | mRNA | -3.44612 | 2.07E-10 | 3.59E-09 |
| DCAF4L2 | mRNA | 9.525143 | 2.28E-10 | 3.91E-09 |
| NRG3 | mRNA | -3.31791 | 2.55E-10 | 4.31E-09 |
| CYP39A1 | mRNA | -4.25771 | 2.69E-10 | 4.53E-09 |
| EPS8L3 | mRNA | 5.272557 | 2.74E-10 | 4.61E-09 |
| GGT5 | mRNA | -2.36131 | 2.82E-10 | 4.72E-09 |
| FREM1 | mRNA | -3.63237 | 2.87E-10 | 4.80E-09 |
| LPA | mRNA | -3.44412 | 3.02E-10 | 5.02E-09 |
| MYBPHL | mRNA | 3.83032 | 3.06E-10 | 5.07E-09 |
| ALPL | mRNA | -2.6707 | 3.07E-10 | 5.09E-09 |
| DNAJC6 | mRNA | 2.46055 | 3.10E-10 | 5.13E-09 |
| TBX4 | mRNA | 5.881395 | 3.52E-10 | 5.75E-09 |
| FITM1 | mRNA | -2.92352 | 3.58E-10 | 5.84E-09 |
| HIST1H2AI | mRNA | 3.437937 | 3.76E-10 | 6.13E-09 |
| SAMD5 | mRNA | -3.7228 | 3.77E-10 | 6.13E-09 |
| CCL4 | mRNA | -2.0145 | 3.91E-10 | 6.33E-09 |
| CYP2B6 | mRNA | -3.37838 | 4.05E-10 | 6.54E-09 |
| APOBEC3A | mRNA | -2.35977 | 4.07E-10 | 6.57E-09 |
| TP73 | mRNA | 2.993737 | 4.23E-10 | 6.78E-09 |
| CR1L | mRNA | -2.85012 | 4.47E-10 | 7.11E-09 |
| CYP3A43 | mRNA | -3.37451 | 4.50E-10 | 7.14E-09 |
| NR4A3 | mRNA | -2.55836 | 4.52E-10 | 7.18E-09 |
| TCF15 | mRNA | 2.35554 | 4.57E-10 | 7.24E-09 |
| DEPDC1B | mRNA | 2.425554 | 4.59E-10 | 7.25E-09 |
| PRRG3 | mRNA | 2.16979 | 4.61E-10 | 7.28E-09 |
| SPATA31D1 | mRNA | 4.594036 | 4.61E-10 | 7.28E-09 |
| C6 | mRNA | -2.46228 | 4.62E-10 | 7.28E-09 |
| GADD45B | mRNA | -2.17478 | 4.74E-10 | 7.44E-09 |
| CAND2 | mRNA | -2.64849 | 4.75E-10 | 7.44E-09 |
| CTNNA3 | mRNA | -3.94888 | 4.83E-10 | 7.53E-09 |
| MAGEA12 | mRNA | 7.282663 | 5.00E-10 | 7.75E-09 |
| LHX4 | mRNA | 2.223982 | 5.10E-10 | 7.87E-09 |
| CYP4A22 | mRNA | -2.85409 | 5.15E-10 | 7.95E-09 |
| CLIC6 | mRNA | -3.44869 | 5.22E-10 | 8.05E-09 |
| MYO3A | mRNA | -3.74781 | 5.26E-10 | 8.09E-09 |
| SYTL5 | mRNA | -2.59605 | 5.36E-10 | 8.23E-09 |
| CPED1 | mRNA | -2.47441 | 5.38E-10 | 8.25E-09 |
| ABCA8 | mRNA | -2.71932 | 5.49E-10 | 8.41E-09 |
| FAM72C | mRNA | 2.970612 | 5.72E-10 | 8.74E-09 |
| PTPN13 | mRNA | -2.34892 | 5.82E-10 | 8.86E-09 |
| DLX5 | mRNA | 4.104804 | 5.99E-10 | 9.08E-09 |
| SELE | mRNA | -2.38001 | 6.03E-10 | 9.12E-09 |
| IL20RA | mRNA | -4.3518 | 6.05E-10 | 9.15E-09 |
| RET | mRNA | -3.60612 | 6.16E-10 | 9.30E-09 |
| IL1RL1 | mRNA | -4.07562 | 6.52E-10 | 9.78E-09 |
| TTC36 | mRNA | -5.23531 | 6.65E-10 | 9.96E-09 |
| PDGFRA | mRNA | -3.36966 | 6.76E-10 | 1.01E-08 |
| SLC16A4 | mRNA | -2.48061 | 6.79E-10 | 1.01E-08 |
| SLITRK3 | mRNA | -6.15876 | 6.91E-10 | 1.03E-08 |
| NIPAL1 | mRNA | -2.43687 | 7.41E-10 | 1.09E-08 |
| SCARA5 | mRNA | -4.75809 | 7.47E-10 | 1.10E-08 |
| FAM198A | mRNA | -3.49715 | 7.49E-10 | 1.10E-08 |
| SCN4A | mRNA | 2.176211 | 7.53E-10 | 1.11E-08 |
| FCAR | mRNA | -2.85797 | 7.64E-10 | 1.12E-08 |
| MAGEC2 | mRNA | 8.077245 | 7.74E-10 | 1.13E-08 |
| FSTL4 | mRNA | 3.841354 | 7.92E-10 | 1.16E-08 |
| OR1F1 | mRNA | 3.696761 | 8.03E-10 | 1.17E-08 |
| CD160 | mRNA | -2.0616 | 8.09E-10 | 1.18E-08 |
| DGKI | mRNA | 2.287648 | 8.13E-10 | 1.18E-08 |
| SLC22A1 | mRNA | -5.06928 | 8.19E-10 | 1.19E-08 |
| SLC7A4 | mRNA | -3.52715 | 8.63E-10 | 1.25E-08 |
| SSX3 | mRNA | 4.594076 | 8.99E-10 | 1.29E-08 |
| TFDP3 | mRNA | 5.191569 | 9.30E-10 | 1.33E-08 |
| MYADML2 | mRNA | 2.734986 | 9.70E-10 | 1.38E-08 |
| MAG | mRNA | -3.1094 | 9.96E-10 | 1.42E-08 |
| DEFA3 | mRNA | -3.7566 | 1.00E-09 | 1.42E-08 |
| VWDE | mRNA | -3.50225 | 1.03E-09 | 1.45E-08 |
| ALX3 | mRNA | 5.241459 | 1.03E-09 | 1.46E-08 |
| NEGR1 | mRNA | -2.30934 | 1.04E-09 | 1.46E-08 |
| CNTN1 | mRNA | -4.13465 | 1.05E-09 | 1.48E-08 |
| CYP4A11 | mRNA | -2.79145 | 1.12E-09 | 1.57E-08 |
| ADRA1B | mRNA | -2.11605 | 1.14E-09 | 1.58E-08 |
| MFAP3L | mRNA | -2.51723 | 1.15E-09 | 1.60E-08 |
| HMOX1 | mRNA | -2.05846 | 1.19E-09 | 1.65E-08 |
| GSTM5 | mRNA | -2.49263 | 1.21E-09 | 1.68E-08 |
| PRUNE2 | mRNA | -2.08387 | 1.24E-09 | 1.71E-08 |
| TPPP2 | mRNA | -3.1714 | 1.25E-09 | 1.73E-08 |
| NRXN1 | mRNA | -4.13588 | 1.25E-09 | 1.73E-08 |
| TMEM132E | mRNA | -2.68841 | 1.28E-09 | 1.76E-08 |
| PPP4R3C | mRNA | 7.718624 | 1.31E-09 | 1.80E-08 |
| PITX1 | mRNA | 5.353453 | 1.34E-09 | 1.84E-08 |
| DNAJC12 | mRNA | -2.68325 | 1.40E-09 | 1.91E-08 |
| GRAMD1C | mRNA | -2.00783 | 1.44E-09 | 1.95E-08 |
| COL9A1 | mRNA | 3.131619 | 1.46E-09 | 1.97E-08 |
| BANF2 | mRNA | 4.352566 | 1.47E-09 | 1.99E-08 |
| KCNK9 | mRNA | 4.870727 | 1.49E-09 | 2.00E-08 |
| CASR | mRNA | -4.1281 | 1.50E-09 | 2.02E-08 |
| HIST1H3B | mRNA | 3.181831 | 1.52E-09 | 2.04E-08 |
| KCNJ15 | mRNA | -2.12248 | 1.54E-09 | 2.06E-08 |
| EPB41L4B | mRNA | -2.10383 | 1.59E-09 | 2.13E-08 |
| HOXD10 | mRNA | 4.225737 | 1.59E-09 | 2.13E-08 |
| HSD17B13 | mRNA | -6.33659 | 1.60E-09 | 2.14E-08 |
| IQCD | mRNA | 2.189494 | 1.62E-09 | 2.15E-08 |
| MAPK8IP2 | mRNA | 2.319677 | 1.64E-09 | 2.18E-08 |
| BMPR1B | mRNA | -3.72659 | 1.67E-09 | 2.22E-08 |
| MMP11 | mRNA | 2.56454 | 1.76E-09 | 2.33E-08 |
| CSMD1 | mRNA | 5.837068 | 1.80E-09 | 2.38E-08 |
| MFAP4 | mRNA | -3.80503 | 1.81E-09 | 2.39E-08 |
| SYT9 | mRNA | -6.02072 | 1.82E-09 | 2.39E-08 |
| AGTR1 | mRNA | -2.18155 | 1.84E-09 | 2.42E-08 |
| BLK | mRNA | -2.59377 | 1.87E-09 | 2.45E-08 |
| C9 | mRNA | -6.01352 | 1.91E-09 | 2.49E-08 |
| CYP3A4 | mRNA | -6.44752 | 1.96E-09 | 2.55E-08 |
| CLDN10 | mRNA | -4.45103 | 2.04E-09 | 2.65E-08 |
| SP8 | mRNA | 4.553767 | 2.04E-09 | 2.66E-08 |
| RPS6KA6 | mRNA | -4.84669 | 2.08E-09 | 2.70E-08 |
| CEACAM3 | mRNA | -2.63288 | 2.13E-09 | 2.75E-08 |
| POU6F2 | mRNA | -4.14679 | 2.14E-09 | 2.76E-08 |
| CCDC196 | mRNA | -3.75513 | 2.25E-09 | 2.90E-08 |
| PGLYRP1 | mRNA | -2.70531 | 2.26E-09 | 2.90E-08 |
| DPF3 | mRNA | -2.20309 | 2.29E-09 | 2.94E-08 |
| GAS1 | mRNA | -2.48512 | 2.38E-09 | 3.04E-08 |
| ANXA10 | mRNA | -2.8225 | 2.39E-09 | 3.06E-08 |
| FBP1 | mRNA | -3.01467 | 2.46E-09 | 3.14E-08 |
| SVEP1 | mRNA | -2.95559 | 2.59E-09 | 3.27E-08 |
| ALKAL2 | mRNA | -3.43849 | 2.67E-09 | 3.36E-08 |
| ANXA8L1 | mRNA | -3.44098 | 2.73E-09 | 3.42E-08 |
| NR4A1 | mRNA | -2.29132 | 2.80E-09 | 3.49E-08 |
| TMEM145 | mRNA | 2.910727 | 2.96E-09 | 3.66E-08 |
| ZNF695 | mRNA | 3.768582 | 2.99E-09 | 3.69E-08 |
| L1CAM | mRNA | -2.68288 | 3.11E-09 | 3.83E-08 |
| COLEC11 | mRNA | -2.35168 | 3.12E-09 | 3.84E-08 |
| GLYATL1 | mRNA | -2.68686 | 3.14E-09 | 3.85E-08 |
| B4GALNT1 | mRNA | 2.815692 | 3.23E-09 | 3.95E-08 |
| SEMA3E | mRNA | -3.8632 | 3.27E-09 | 3.99E-08 |
| CDHR2 | mRNA | -4.5983 | 3.33E-09 | 4.05E-08 |
| B4GALNT2 | mRNA | 6.074551 | 3.39E-09 | 4.12E-08 |
| BEX1 | mRNA | -4.22752 | 3.40E-09 | 4.13E-08 |
| TSPAN11 | mRNA | -2.27801 | 3.41E-09 | 4.14E-08 |
| GPR19 | mRNA | 2.07564 | 3.53E-09 | 4.25E-08 |
| GADD45G | mRNA | -2.11733 | 3.57E-09 | 4.29E-08 |
| IGLON5 | mRNA | -3.33519 | 3.58E-09 | 4.30E-08 |
| SLC22A10 | mRNA | -3.22795 | 3.59E-09 | 4.31E-08 |
| ABI3BP | mRNA | -2.12516 | 3.60E-09 | 4.32E-08 |
| HIST1H2AM | mRNA | 2.398578 | 3.68E-09 | 4.39E-08 |
| HAO2 | mRNA | -4.9605 | 3.72E-09 | 4.43E-08 |
| GPD1 | mRNA | -2.51509 | 3.75E-09 | 4.46E-08 |
| GPC3 | mRNA | 4.229018 | 3.81E-09 | 4.53E-08 |
| CYP2C9 | mRNA | -3.09488 | 3.84E-09 | 4.57E-08 |
| ALDH8A1 | mRNA | -2.62724 | 4.00E-09 | 4.74E-08 |
| RCVRN | mRNA | -2.40079 | 4.05E-09 | 4.79E-08 |
| GPR83 | mRNA | -2.25854 | 4.07E-09 | 4.81E-08 |
| LRCOL1 | mRNA | -3.80267 | 4.16E-09 | 4.91E-08 |
| LYPD8 | mRNA | 4.921833 | 4.16E-09 | 4.91E-08 |
| ZDBF2 | mRNA | -2.14299 | 4.18E-09 | 4.92E-08 |
| FOXS1 | mRNA | 2.537225 | 4.36E-09 | 5.13E-08 |
| KCNE1 | mRNA | -2.47104 | 4.40E-09 | 5.17E-08 |
| IGF2BP1 | mRNA | 5.584591 | 4.59E-09 | 5.37E-08 |
| NRK | mRNA | -2.77474 | 4.65E-09 | 5.43E-08 |
| PRR18 | mRNA | -2.25143 | 4.66E-09 | 5.43E-08 |
| EPHB1 | mRNA | -2.22687 | 4.72E-09 | 5.49E-08 |
| ADAMTSL2 | mRNA | -2.70635 | 4.81E-09 | 5.58E-08 |
| SERPINA11 | mRNA | -3.1097 | 4.85E-09 | 5.62E-08 |
| FXYD1 | mRNA | -4.1948 | 5.00E-09 | 5.77E-08 |
| IYD | mRNA | -2.45908 | 5.00E-09 | 5.77E-08 |
| KLKB1 | mRNA | -2.00119 | 5.07E-09 | 5.83E-08 |
| TRIM71 | mRNA | 5.811972 | 5.17E-09 | 5.94E-08 |
| ATOH8 | mRNA | -2.31604 | 5.22E-09 | 5.98E-08 |
| PLGLB2 | mRNA | -2.68878 | 5.22E-09 | 5.98E-08 |
| MCEMP1 | mRNA | -2.52708 | 5.27E-09 | 6.03E-08 |
| SLCO1B3 | mRNA | -6.89968 | 5.28E-09 | 6.04E-08 |
| HOGA1 | mRNA | -2.35891 | 5.29E-09 | 6.04E-08 |
| FAM83B | mRNA | -3.57969 | 5.46E-09 | 6.21E-08 |
| DSCAM | mRNA | -2.54272 | 5.50E-09 | 6.24E-08 |
| CLC | mRNA | -3.07525 | 5.56E-09 | 6.29E-08 |
| B3GALT5 | mRNA | -3.95227 | 5.60E-09 | 6.33E-08 |
| ACSL1 | mRNA | -2.40168 | 5.68E-09 | 6.40E-08 |
| ERVFRD-1 | mRNA | -2.64054 | 6.05E-09 | 6.78E-08 |
| RFX8 | mRNA | 2.363134 | 6.26E-09 | 6.98E-08 |
| CREG2 | mRNA | 2.645392 | 6.40E-09 | 7.13E-08 |
| ENO3 | mRNA | -2.73089 | 6.63E-09 | 7.36E-08 |
| CDA | mRNA | -2.44598 | 6.66E-09 | 7.38E-08 |
| NRG1 | mRNA | -3.91081 | 6.71E-09 | 7.44E-08 |
| AMPD1 | mRNA | -3.59414 | 7.03E-09 | 7.76E-08 |
| DUSP9 | mRNA | 2.692782 | 7.13E-09 | 7.85E-08 |
| STRIP2 | mRNA | 2.176385 | 7.42E-09 | 8.12E-08 |
| HBA1 | mRNA | -2.72173 | 7.42E-09 | 8.12E-08 |
| PTP4A3 | mRNA | 2.136762 | 7.42E-09 | 8.12E-08 |
| ZNF716 | mRNA | 4.946507 | 7.97E-09 | 8.64E-08 |
| DACH2 | mRNA | 3.640462 | 8.27E-09 | 8.92E-08 |
| MKRN3 | mRNA | 3.562872 | 8.31E-09 | 8.95E-08 |
| SLCO4C1 | mRNA | -4.41025 | 8.45E-09 | 9.10E-08 |
| GGT6 | mRNA | -4.46251 | 8.50E-09 | 9.13E-08 |
| MIP | mRNA | -2.37695 | 8.67E-09 | 9.30E-08 |
| COL28A1 | mRNA | -3.03975 | 8.77E-09 | 9.38E-08 |
| ASS1 | mRNA | -2.13176 | 8.80E-09 | 9.41E-08 |
| GPRIN2 | mRNA | -4.11122 | 8.89E-09 | 9.48E-08 |
| RTKN2 | mRNA | 2.451961 | 9.28E-09 | 9.83E-08 |
| B3GAT1 | mRNA | -3.52285 | 9.32E-09 | 9.87E-08 |
| SLC19A3 | mRNA | -2.12628 | 9.68E-09 | 1.02E-07 |
| GSDMC | mRNA | 3.356905 | 1.00E-08 | 1.06E-07 |
| IGF2 | mRNA | -4.94155 | 1.06E-08 | 1.11E-07 |
| RIC3 | mRNA | -3.32608 | 1.09E-08 | 1.14E-07 |
| VGLL1 | mRNA | -3.47978 | 1.09E-08 | 1.14E-07 |
| OMG | mRNA | -2.2037 | 1.10E-08 | 1.15E-07 |
| KCNA3 | mRNA | -2.09677 | 1.11E-08 | 1.16E-07 |
| UGT2B7 | mRNA | -2.67131 | 1.13E-08 | 1.18E-07 |
| KCNJ10 | mRNA | -2.95354 | 1.18E-08 | 1.22E-07 |
| CTAG2 | mRNA | 7.83173 | 1.23E-08 | 1.27E-07 |
| FCRL5 | mRNA | -3.341 | 1.30E-08 | 1.33E-07 |
| IRF4 | mRNA | -2.57667 | 1.33E-08 | 1.35E-07 |
| BASP1 | mRNA | -2.1312 | 1.33E-08 | 1.36E-07 |
| TCEAL2 | mRNA | -3.20122 | 1.34E-08 | 1.36E-07 |
| HIST2H4A | mRNA | 2.243322 | 1.38E-08 | 1.40E-07 |
| KIAA1456 | mRNA | -2.46039 | 1.40E-08 | 1.42E-07 |
| C1QTNF7 | mRNA | -2.11566 | 1.45E-08 | 1.47E-07 |
| RDH16 | mRNA | -3.44725 | 1.46E-08 | 1.48E-07 |
| RDM1 | mRNA | 2.991087 | 1.48E-08 | 1.49E-07 |
| ANXA3 | mRNA | -2.37257 | 1.49E-08 | 1.50E-07 |
| ISL2 | mRNA | 3.638652 | 1.52E-08 | 1.52E-07 |
| CSMD2 | mRNA | 2.960871 | 1.52E-08 | 1.53E-07 |
| CMTM5 | mRNA | -2.73684 | 1.56E-08 | 1.56E-07 |
| MAGEA6 | mRNA | 7.342805 | 1.57E-08 | 1.57E-07 |
| HIST1H2AL | mRNA | 2.725979 | 1.61E-08 | 1.61E-07 |
| PLXNA4 | mRNA | -2.93248 | 1.70E-08 | 1.69E-07 |
| NPFFR2 | mRNA | 3.767127 | 1.72E-08 | 1.71E-07 |
| HOXB9 | mRNA | 3.097399 | 1.92E-08 | 1.89E-07 |
| EPO | mRNA | -4.02445 | 1.93E-08 | 1.89E-07 |
| EPHX4 | mRNA | 2.668592 | 1.96E-08 | 1.92E-07 |
| TGIF2LX | mRNA | 4.813512 | 1.97E-08 | 1.92E-07 |
| PI16 | mRNA | -3.45333 | 2.04E-08 | 1.98E-07 |
| GALNT3 | mRNA | -2.28065 | 2.16E-08 | 2.08E-07 |
| WDR72 | mRNA | -3.24151 | 2.22E-08 | 2.13E-07 |
| KANK4 | mRNA | -3.87005 | 2.26E-08 | 2.17E-07 |
| RETREG1 | mRNA | -3.3544 | 2.28E-08 | 2.19E-07 |
| MUM1L1 | mRNA | -4.24691 | 2.30E-08 | 2.21E-07 |
| NPY5R | mRNA | -2.98582 | 2.34E-08 | 2.24E-07 |
| AKR1D1 | mRNA | -3.98491 | 2.43E-08 | 2.31E-07 |
| PCK1 | mRNA | -3.44916 | 2.44E-08 | 2.32E-07 |
| TNR | mRNA | -4.01221 | 2.61E-08 | 2.47E-07 |
| CYP2C19 | mRNA | -4.59361 | 2.66E-08 | 2.51E-07 |
| BICDL1 | mRNA | 2.004848 | 2.70E-08 | 2.54E-07 |
| SSC5D | mRNA | -2.25184 | 2.70E-08 | 2.54E-07 |
| TNFRSF17 | mRNA | -3.14693 | 2.77E-08 | 2.59E-07 |
| TGFA | mRNA | -2.21553 | 2.82E-08 | 2.63E-07 |
| ANK3 | mRNA | -2.22949 | 2.82E-08 | 2.63E-07 |
| PDE1C | mRNA | 2.119312 | 2.84E-08 | 2.65E-07 |
| IL1RAPL2 | mRNA | -3.02647 | 2.87E-08 | 2.68E-07 |
| TMEM45A | mRNA | -2.44863 | 2.88E-08 | 2.68E-07 |
| ADH4 | mRNA | -4.16407 | 2.91E-08 | 2.70E-07 |
| OR51E2 | mRNA | 2.998087 | 2.97E-08 | 2.75E-07 |
| SAA4 | mRNA | -3.08364 | 3.00E-08 | 2.78E-07 |
| BBOX1 | mRNA | -3.13931 | 3.15E-08 | 2.90E-07 |
| FAM83E | mRNA | -2.95986 | 3.18E-08 | 2.92E-07 |
| SLITRK2 | mRNA | -3.06812 | 3.28E-08 | 3.00E-07 |
| DPPA4 | mRNA | -3.02095 | 3.37E-08 | 3.07E-07 |
| RASSF9 | mRNA | -2.02328 | 3.37E-08 | 3.07E-07 |
| CRYBG2 | mRNA | 2.604905 | 3.40E-08 | 3.09E-07 |
| AGXT2 | mRNA | -2.62432 | 3.43E-08 | 3.12E-07 |
| RHO | mRNA | 2.852591 | 3.74E-08 | 3.38E-07 |
| XG | mRNA | -2.9649 | 3.75E-08 | 3.38E-07 |
| PDIA2 | mRNA | 3.665097 | 3.75E-08 | 3.38E-07 |
| HOXA10 | mRNA | 3.814514 | 3.77E-08 | 3.40E-07 |
| DOK6 | mRNA | -2.04712 | 3.79E-08 | 3.41E-07 |
| CARD18 | mRNA | 4.330345 | 3.91E-08 | 3.49E-07 |
| C11orf96 | mRNA | -2.05814 | 3.91E-08 | 3.49E-07 |
| KLK11 | mRNA | -4.47676 | 4.09E-08 | 3.63E-07 |
| DTX1 | mRNA | -2.58906 | 4.24E-08 | 3.75E-07 |
| IL7R | mRNA | -2.11161 | 4.32E-08 | 3.82E-07 |
| CDH8 | mRNA | 3.413855 | 4.41E-08 | 3.89E-07 |
| KCNN1 | mRNA | 2.529504 | 4.47E-08 | 3.93E-07 |
| AVPR1A | mRNA | -4.70111 | 4.50E-08 | 3.96E-07 |
| NTRK3 | mRNA | -2.45243 | 4.65E-08 | 4.07E-07 |
| EYA2 | mRNA | -2.33979 | 4.86E-08 | 4.24E-07 |
| ADH1B | mRNA | -2.6317 | 4.98E-08 | 4.33E-07 |
| BICDL2 | mRNA | -3.77425 | 5.05E-08 | 4.38E-07 |
| CAMP | mRNA | -2.63733 | 5.06E-08 | 4.39E-07 |
| MOGAT2 | mRNA | -3.87826 | 5.09E-08 | 4.41E-07 |
| CBLN2 | mRNA | -3.69509 | 5.09E-08 | 4.41E-07 |
| GYS2 | mRNA | -3.60131 | 5.17E-08 | 4.46E-07 |
| ERBB4 | mRNA | -2.66386 | 5.18E-08 | 4.46E-07 |
| IGLL5 | mRNA | -4.09183 | 5.19E-08 | 4.47E-07 |
| ATAD3C | mRNA | -2.19456 | 5.24E-08 | 4.51E-07 |
| PLGLB1 | mRNA | -2.17433 | 5.30E-08 | 4.56E-07 |
| MRAP2 | mRNA | 3.313936 | 5.33E-08 | 4.58E-07 |
| PTN | mRNA | -2.03253 | 5.52E-08 | 4.72E-07 |
| TAL2 | mRNA | 2.650807 | 5.66E-08 | 4.82E-07 |
| MAGEC1 | mRNA | 7.97751 | 5.70E-08 | 4.85E-07 |
| ALLC | mRNA | -3.05486 | 5.72E-08 | 4.85E-07 |
| HSF2BP | mRNA | 2.000455 | 6.05E-08 | 5.10E-07 |
| SCN7A | mRNA | -3.37751 | 6.05E-08 | 5.10E-07 |
| TMEM82 | mRNA | -3.3362 | 6.08E-08 | 5.12E-07 |
| HIST1H2BL | mRNA | 2.784712 | 6.31E-08 | 5.30E-07 |
| C3orf85 | mRNA | -3.53705 | 6.50E-08 | 5.44E-07 |
| PNMA2 | mRNA | -2.1403 | 6.68E-08 | 5.57E-07 |
| TINAG | mRNA | 5.9442 | 6.99E-08 | 5.80E-07 |
| LGI1 | mRNA | -3.57676 | 7.11E-08 | 5.90E-07 |
| VNN1 | mRNA | -2.7958 | 7.22E-08 | 5.97E-07 |
| MYCN | mRNA | 2.765073 | 7.24E-08 | 5.99E-07 |
| ITGB8 | mRNA | -2.97988 | 7.28E-08 | 6.02E-07 |
| SEPT3 | mRNA | 2.361846 | 7.39E-08 | 6.10E-07 |
| SLC27A6 | mRNA | -2.83041 | 7.40E-08 | 6.10E-07 |
| DCAF8L1 | mRNA | 5.961864 | 7.44E-08 | 6.13E-07 |
| HHIPL2 | mRNA | 3.905009 | 7.52E-08 | 6.19E-07 |
| SLC1A1 | mRNA | -2.04487 | 7.64E-08 | 6.27E-07 |
| ADH1A | mRNA | -2.69042 | 7.71E-08 | 6.32E-07 |
| C2CD4B | mRNA | -2.1135 | 7.76E-08 | 6.36E-07 |
| SFN | mRNA | 3.669679 | 7.81E-08 | 6.38E-07 |
| COL24A1 | mRNA | 2.736481 | 7.85E-08 | 6.41E-07 |
| ARHGAP20 | mRNA | -2.02453 | 7.96E-08 | 6.50E-07 |
| SORCS1 | mRNA | -2.92769 | 8.29E-08 | 6.74E-07 |
| SCX | mRNA | 2.286655 | 8.31E-08 | 6.75E-07 |
| VNN3 | mRNA | -2.36353 | 8.51E-08 | 6.90E-07 |
| RNF150 | mRNA | -2.47509 | 8.65E-08 | 7.00E-07 |
| CTHRC1 | mRNA | 2.541269 | 8.67E-08 | 7.01E-07 |
| TTC39A | mRNA | 2.500314 | 8.79E-08 | 7.08E-07 |
| TERB2 | mRNA | -3.23466 | 9.03E-08 | 7.26E-07 |
| CACNA1S | mRNA | 2.333982 | 9.12E-08 | 7.33E-07 |
| TMEM200C | mRNA | -2.2217 | 9.63E-08 | 7.70E-07 |
| ADH1C | mRNA | -3.50177 | 9.69E-08 | 7.75E-07 |
| SLAIN1 | mRNA | -3.02683 | 9.80E-08 | 7.83E-07 |
| AC093155.3 | mRNA | -2.25138 | 9.94E-08 | 7.93E-07 |
| SPIC | mRNA | -2.52402 | 1.03E-07 | 8.19E-07 |
| SOX6 | mRNA | -2.09089 | 1.04E-07 | 8.29E-07 |
| KMO | mRNA | -2.81142 | 1.07E-07 | 8.49E-07 |
| C8A | mRNA | -2.26402 | 1.11E-07 | 8.72E-07 |
| MEGF10 | mRNA | -2.7187 | 1.11E-07 | 8.73E-07 |
| AGBL4 | mRNA | -2.95279 | 1.16E-07 | 9.06E-07 |
| CD69 | mRNA | -2.02701 | 1.18E-07 | 9.21E-07 |
| LRRC19 | mRNA | -2.95588 | 1.18E-07 | 9.24E-07 |
| ACOT12 | mRNA | -2.28416 | 1.19E-07 | 9.26E-07 |
| NCAM2 | mRNA | -3.0127 | 1.20E-07 | 9.33E-07 |
| ZNF735 | mRNA | 4.020973 | 1.20E-07 | 9.38E-07 |
| CCL2 | mRNA | -2.01254 | 1.24E-07 | 9.61E-07 |
| NRG2 | mRNA | 2.311031 | 1.25E-07 | 9.66E-07 |
| BAIAP2L2 | mRNA | 2.199744 | 1.26E-07 | 9.72E-07 |
| PLCXD3 | mRNA | -3.07531 | 1.27E-07 | 9.80E-07 |
| TGM3 | mRNA | 3.827007 | 1.28E-07 | 9.86E-07 |
| MAT1A | mRNA | -2.11862 | 1.28E-07 | 9.86E-07 |
| DSCR8 | mRNA | 5.359939 | 1.28E-07 | 9.87E-07 |
| OGDHL | mRNA | -2.30914 | 1.30E-07 | 9.98E-07 |
| AJAP1 | mRNA | -2.85035 | 1.30E-07 | 1.00E-06 |
| GPRIN1 | mRNA | 2.482596 | 1.34E-07 | 1.02E-06 |
| DAND5 | mRNA | 2.486527 | 1.37E-07 | 1.04E-06 |
| MEP1B | mRNA | -3.75287 | 1.40E-07 | 1.06E-06 |
| HOXB8 | mRNA | 2.774828 | 1.44E-07 | 1.09E-06 |
| FTCD | mRNA | -2.15651 | 1.47E-07 | 1.11E-06 |
| ATF5 | mRNA | -2.8009 | 1.50E-07 | 1.13E-06 |
| NOL4 | mRNA | -3.54574 | 1.50E-07 | 1.13E-06 |
| PYROXD2 | mRNA | -2.27647 | 1.52E-07 | 1.14E-06 |
| ANO1 | mRNA | -2.34031 | 1.53E-07 | 1.15E-06 |
| SAMD12 | mRNA | -2.55776 | 1.58E-07 | 1.18E-06 |
| IGF2BP3 | mRNA | 2.897066 | 1.64E-07 | 1.22E-06 |
| LOXHD1 | mRNA | 2.69186 | 1.65E-07 | 1.22E-06 |
| SAMD11 | mRNA | -2.52896 | 1.69E-07 | 1.25E-06 |
| RGS9BP | mRNA | 2.547978 | 1.69E-07 | 1.25E-06 |
| IGSF3 | mRNA | 2.094264 | 1.72E-07 | 1.26E-06 |
| CPA6 | mRNA | 3.199039 | 1.73E-07 | 1.27E-06 |
| HOXC9 | mRNA | 3.775118 | 1.76E-07 | 1.29E-06 |
| CELF3 | mRNA | 2.629729 | 1.78E-07 | 1.30E-06 |
| RALYL | mRNA | -3.05916 | 1.82E-07 | 1.33E-06 |
| VWA2 | mRNA | -2.43409 | 1.85E-07 | 1.35E-06 |
| PROZ | mRNA | -2.19043 | 1.88E-07 | 1.37E-06 |
| RETNLB | mRNA | 3.923279 | 1.88E-07 | 1.37E-06 |
| UROC1 | mRNA | -5.30519 | 2.06E-07 | 1.48E-06 |
| PHGDH | mRNA | -2.60483 | 2.11E-07 | 1.52E-06 |
| MTUS2 | mRNA | -3.62605 | 2.15E-07 | 1.54E-06 |
| SPP2 | mRNA | -3.45441 | 2.16E-07 | 1.55E-06 |
| CAMK2N2 | mRNA | 2.114231 | 2.17E-07 | 1.55E-06 |
| SGCA | mRNA | -2.52909 | 2.19E-07 | 1.57E-06 |
| GBX2 | mRNA | 2.950588 | 2.21E-07 | 1.58E-06 |
| ADH6 | mRNA | -2.06523 | 2.32E-07 | 1.65E-06 |
| FGB | mRNA | -2.23295 | 2.42E-07 | 1.72E-06 |
| ORM1 | mRNA | -2.29813 | 2.50E-07 | 1.77E-06 |
| MAFA | mRNA | 3.940014 | 2.52E-07 | 1.78E-06 |
| C5orf46 | mRNA | 3.066065 | 2.52E-07 | 1.78E-06 |
| SEZ6L | mRNA | -2.40191 | 2.56E-07 | 1.81E-06 |
| GLP1R | mRNA | 3.735274 | 2.60E-07 | 1.83E-06 |
| LDLRAD1 | mRNA | 3.568166 | 2.67E-07 | 1.87E-06 |
| AFM | mRNA | -2.77563 | 2.67E-07 | 1.87E-06 |
| TDO2 | mRNA | -3.09482 | 2.67E-07 | 1.87E-06 |
| DHRS7C | mRNA | -3.16278 | 2.68E-07 | 1.88E-06 |
| GPR158 | mRNA | 3.351329 | 2.70E-07 | 1.89E-06 |
| CADM2 | mRNA | -3.357 | 2.92E-07 | 2.02E-06 |
| MAGEA3 | mRNA | 6.933228 | 2.92E-07 | 2.02E-06 |
| GDF6 | mRNA | -2.30877 | 2.93E-07 | 2.03E-06 |
| UCN2 | mRNA | 2.685898 | 3.08E-07 | 2.12E-06 |
| SERPINA3 | mRNA | -2.21061 | 3.09E-07 | 2.13E-06 |
| FOLR3 | mRNA | -2.17366 | 3.12E-07 | 2.15E-06 |
| SCG2 | mRNA | 3.096139 | 3.12E-07 | 2.15E-06 |
| EXOC3L4 | mRNA | -2.23274 | 3.14E-07 | 2.16E-06 |
| ZG16 | mRNA | -3.42537 | 3.28E-07 | 2.25E-06 |
| SLC27A2 | mRNA | -2.06801 | 3.35E-07 | 2.29E-06 |
| DNAH6 | mRNA | -2.38804 | 3.36E-07 | 2.30E-06 |
| HP | mRNA | -2.71176 | 3.45E-07 | 2.35E-06 |
| FLRT2 | mRNA | -2.39332 | 3.51E-07 | 2.39E-06 |
| CD79A | mRNA | -2.65085 | 3.53E-07 | 2.40E-06 |
| ACAN | mRNA | 3.014551 | 3.58E-07 | 2.43E-06 |
| GABRG2 | mRNA | 4.790673 | 3.63E-07 | 2.46E-06 |
| FOXP2 | mRNA | -4.02933 | 3.66E-07 | 2.48E-06 |
| MUC13 | mRNA | 3.997516 | 3.66E-07 | 2.48E-06 |
| IL1RN | mRNA | -2.47261 | 3.88E-07 | 2.61E-06 |
| CSAG1 | mRNA | 5.550289 | 4.11E-07 | 2.76E-06 |
| PHYHD1 | mRNA | -2.38996 | 4.25E-07 | 2.84E-06 |
| CWH43 | mRNA | -3.81597 | 4.29E-07 | 2.86E-06 |
| TCL1A | mRNA | -2.53311 | 4.40E-07 | 2.93E-06 |
| BPIFA2 | mRNA | 3.725646 | 4.49E-07 | 2.99E-06 |
| ALDH1A3 | mRNA | -2.11563 | 4.54E-07 | 3.01E-06 |
| PRND | mRNA | 2.678586 | 4.59E-07 | 3.04E-06 |
| C20orf144 | mRNA | 2.025656 | 4.60E-07 | 3.04E-06 |
| CNGA1 | mRNA | -2.28428 | 4.61E-07 | 3.04E-06 |
| BRINP1 | mRNA | -2.44723 | 4.64E-07 | 3.06E-06 |
| ASPG | mRNA | -4.23482 | 4.70E-07 | 3.10E-06 |
| GRM8 | mRNA | -3.34028 | 4.74E-07 | 3.12E-06 |
| CXCL6 | mRNA | -3.6979 | 4.77E-07 | 3.14E-06 |
| LYPD1 | mRNA | 3.47116 | 4.78E-07 | 3.14E-06 |
| PRELP | mRNA | -2.34405 | 4.80E-07 | 3.15E-06 |
| GLS2 | mRNA | -4.29262 | 4.81E-07 | 3.16E-06 |
| VTCN1 | mRNA | -3.7403 | 4.94E-07 | 3.23E-06 |
| OLFM1 | mRNA | -2.07261 | 5.06E-07 | 3.31E-06 |
| CLSTN2 | mRNA | -2.18465 | 5.06E-07 | 3.31E-06 |
| ST6GALNAC1 | mRNA | -2.23511 | 5.17E-07 | 3.37E-06 |
| PPP1R1C | mRNA | -2.83386 | 5.21E-07 | 3.39E-06 |
| TBX15 | mRNA | -2.99648 | 5.23E-07 | 3.40E-06 |
| KCND3 | mRNA | -2.8903 | 5.35E-07 | 3.47E-06 |
| IGF1 | mRNA | -2.33724 | 5.39E-07 | 3.49E-06 |
| HBD | mRNA | -2.81839 | 5.55E-07 | 3.58E-06 |
| SLC27A5 | mRNA | -2.37028 | 5.81E-07 | 3.73E-06 |
| MAGEB1 | mRNA | 5.159486 | 5.87E-07 | 3.75E-06 |
| TDRD1 | mRNA | -3.15984 | 6.11E-07 | 3.89E-06 |
| GLDN | mRNA | 2.697646 | 6.12E-07 | 3.89E-06 |
| KRT19 | mRNA | -4.18244 | 6.14E-07 | 3.91E-06 |
| CHRNB4 | mRNA | 2.447679 | 6.28E-07 | 3.98E-06 |
| SPATC1L | mRNA | 2.034702 | 6.40E-07 | 4.05E-06 |
| ADCY1 | mRNA | -2.80754 | 6.42E-07 | 4.06E-06 |
| MFSD6L | mRNA | -2.50584 | 6.55E-07 | 4.13E-06 |
| ARID3C | mRNA | -2.09579 | 6.67E-07 | 4.19E-06 |
| MBL2 | mRNA | -2.7671 | 6.72E-07 | 4.22E-06 |
| VSIG2 | mRNA | -2.61644 | 6.78E-07 | 4.25E-06 |
| SLC5A7 | mRNA | -2.91004 | 7.55E-07 | 4.69E-06 |
| DES | mRNA | -2.89617 | 7.62E-07 | 4.73E-06 |
| ESRP1 | mRNA | -3.71317 | 7.86E-07 | 4.87E-06 |
| KCNK3 | mRNA | -2.08207 | 7.91E-07 | 4.89E-06 |
| MT1A | mRNA | -2.88719 | 8.01E-07 | 4.94E-06 |
| SPOCK1 | mRNA | 3.086509 | 8.17E-07 | 5.02E-06 |
| KRTAP5-5 | mRNA | 4.201177 | 8.25E-07 | 5.06E-06 |
| SLC22A8 | mRNA | 3.629661 | 8.52E-07 | 5.21E-06 |
| HIST1H4E | mRNA | 2.441094 | 8.64E-07 | 5.28E-06 |
| BNC1 | mRNA | -2.25546 | 8.71E-07 | 5.32E-06 |
| PF4 | mRNA | -2.36729 | 8.92E-07 | 5.44E-06 |
| KRTCAP3 | mRNA | -2.83603 | 8.95E-07 | 5.45E-06 |
| CAMK2B | mRNA | -3.08166 | 9.06E-07 | 5.51E-06 |
| LEF1 | mRNA | 2.595264 | 9.29E-07 | 5.64E-06 |
| CCL21 | mRNA | -2.99006 | 9.33E-07 | 5.66E-06 |
| TMEM132C | mRNA | -3.58588 | 9.62E-07 | 5.82E-06 |
| TRPM8 | mRNA | -2.4448 | 9.98E-07 | 6.01E-06 |
| SH2D4B | mRNA | 2.778892 | 1.02E-06 | 6.14E-06 |
| VSTM1 | mRNA | -2.17861 | 1.05E-06 | 6.28E-06 |
| PGLYRP2 | mRNA | -2.95807 | 1.05E-06 | 6.31E-06 |
| CRTAC1 | mRNA | -2.70871 | 1.06E-06 | 6.31E-06 |
| KCNC1 | mRNA | 3.104487 | 1.07E-06 | 6.39E-06 |
| ESRRG | mRNA | -2.80612 | 1.08E-06 | 6.45E-06 |
| ELFN2 | mRNA | 3.2829 | 1.09E-06 | 6.52E-06 |
| LEAP2 | mRNA | -2.00102 | 1.11E-06 | 6.62E-06 |
| CNKSR1 | mRNA | -2.87991 | 1.12E-06 | 6.64E-06 |
| ADGRE3 | mRNA | -2.0973 | 1.13E-06 | 6.67E-06 |
| PLPPR4 | mRNA | 2.57331 | 1.13E-06 | 6.69E-06 |
| SAPCD1 | mRNA | 2.012151 | 1.14E-06 | 6.72E-06 |
| RBM24 | mRNA | 2.074194 | 1.14E-06 | 6.73E-06 |
| SAA2 | mRNA | -4.22905 | 1.15E-06 | 6.79E-06 |
| CCL19 | mRNA | -3.24592 | 1.16E-06 | 6.85E-06 |
| RBM11 | mRNA | -2.14761 | 1.17E-06 | 6.90E-06 |
| PLCZ1 | mRNA | -2.34159 | 1.23E-06 | 7.20E-06 |
| PF4V1 | mRNA | -2.75619 | 1.23E-06 | 7.22E-06 |
| PGC | mRNA | 4.797806 | 1.24E-06 | 7.26E-06 |
| FGF10 | mRNA | -2.54346 | 1.25E-06 | 7.29E-06 |
| SLC46A3 | mRNA | -2.10491 | 1.27E-06 | 7.39E-06 |
| MPPED1 | mRNA | -2.96375 | 1.28E-06 | 7.47E-06 |
| F9 | mRNA | -2.99797 | 1.29E-06 | 7.51E-06 |
| MS4A1 | mRNA | -2.43914 | 1.34E-06 | 7.79E-06 |
| DSCR4 | mRNA | 5.232283 | 1.35E-06 | 7.81E-06 |
| GLYAT | mRNA | -3.28915 | 1.38E-06 | 7.99E-06 |
| ASPDH | mRNA | -2.48485 | 1.41E-06 | 8.11E-06 |
| FCRL2 | mRNA | -2.68622 | 1.41E-06 | 8.12E-06 |
| PIK3C2G | mRNA | -2.7273 | 1.41E-06 | 8.13E-06 |
| MYOT | mRNA | -2.46539 | 1.43E-06 | 8.24E-06 |
| A1BG | mRNA | -2.34667 | 1.46E-06 | 8.39E-06 |
| ACSM5 | mRNA | -2.43004 | 1.47E-06 | 8.46E-06 |
| ALB | mRNA | -2.45258 | 1.47E-06 | 8.46E-06 |
| SLC51B | mRNA | 2.271927 | 1.48E-06 | 8.47E-06 |
| GJA10 | mRNA | 4.896323 | 1.49E-06 | 8.53E-06 |
| CCR9 | mRNA | -2.11127 | 1.49E-06 | 8.54E-06 |
| BCHE | mRNA | -3.56282 | 1.51E-06 | 8.65E-06 |
| ABCB5 | mRNA | 4.238484 | 1.52E-06 | 8.70E-06 |
| NKX3-2 | mRNA | 3.338338 | 1.52E-06 | 8.71E-06 |
| REG1A | mRNA | 5.120727 | 1.52E-06 | 8.72E-06 |
| C10orf142 | mRNA | -2.32929 | 1.55E-06 | 8.83E-06 |
| THRSP | mRNA | -4.27178 | 1.56E-06 | 8.89E-06 |
| EPHA7 | mRNA | -2.66985 | 1.60E-06 | 9.11E-06 |
| MEI4 | mRNA | -2.85207 | 1.63E-06 | 9.28E-06 |
| HCN2 | mRNA | 2.358987 | 1.71E-06 | 9.66E-06 |
| IRX6 | mRNA | 3.301879 | 1.72E-06 | 9.68E-06 |
| NPBWR1 | mRNA | -3.33354 | 1.73E-06 | 9.74E-06 |
| TCP10L | mRNA | -2.37483 | 1.73E-06 | 9.75E-06 |
| CHGA | mRNA | 2.747128 | 1.75E-06 | 9.87E-06 |
| S100A3 | mRNA | 2.420309 | 1.76E-06 | 9.92E-06 |
| CLEC12B | mRNA | -2.2252 | 1.78E-06 | 9.98E-06 |
| GNGT1 | mRNA | 3.355517 | 1.78E-06 | 9.99E-06 |
| PLG | mRNA | -2.00192 | 1.82E-06 | 1.02E-05 |
| EXPH5 | mRNA | -2.45088 | 1.83E-06 | 1.02E-05 |
| PCDHGA1 | mRNA | 2.296901 | 1.84E-06 | 1.03E-05 |
| TPTE | mRNA | 4.495329 | 1.90E-06 | 1.06E-05 |
| CEACAM6 | mRNA | -4.02555 | 1.93E-06 | 1.07E-05 |
| SPINK5 | mRNA | 2.388696 | 1.94E-06 | 1.07E-05 |
| GAST | mRNA | 3.620414 | 1.95E-06 | 1.08E-05 |
| AC092042.3 | mRNA | -2.14928 | 1.97E-06 | 1.09E-05 |
| XAGE5 | mRNA | 5.98271 | 2.08E-06 | 1.14E-05 |
| GPR15 | mRNA | -2.62865 | 2.20E-06 | 1.20E-05 |
| HIST2H3D | mRNA | 2.142117 | 2.24E-06 | 1.22E-05 |
| AOC1 | mRNA | -2.39804 | 2.24E-06 | 1.22E-05 |
| PKHD1 | mRNA | -2.59282 | 2.25E-06 | 1.23E-05 |
| CYP3A7 | mRNA | -2.83735 | 2.28E-06 | 1.24E-05 |
| FOXD3 | mRNA | -3.09956 | 2.39E-06 | 1.29E-05 |
| ST8SIA3 | mRNA | -2.86101 | 2.40E-06 | 1.30E-05 |
| AC011604.2 | mRNA | -3.24939 | 2.46E-06 | 1.33E-05 |
| TMEM100 | mRNA | -2.01817 | 2.47E-06 | 1.33E-05 |
| SLC38A4 | mRNA | -2.16188 | 2.57E-06 | 1.38E-05 |
| PODN | mRNA | -2.51754 | 2.62E-06 | 1.40E-05 |
| FEZF1 | mRNA | 3.623943 | 2.63E-06 | 1.40E-05 |
| SEZ6L2 | mRNA | 3.399678 | 2.63E-06 | 1.40E-05 |
| PLIN1 | mRNA | -2.47156 | 2.64E-06 | 1.41E-05 |
| VCX3A | mRNA | 3.45313 | 2.70E-06 | 1.44E-05 |
| UPK1B | mRNA | -2.81331 | 2.77E-06 | 1.47E-05 |
| RNF212 | mRNA | -2.16205 | 2.81E-06 | 1.49E-05 |
| PHEX | mRNA | 2.298243 | 2.87E-06 | 1.52E-05 |
| PDZRN3 | mRNA | -2.05655 | 2.87E-06 | 1.52E-05 |
| HEPACAM | mRNA | -5.01518 | 2.88E-06 | 1.52E-05 |
| BHLHA9 | mRNA | -2.98348 | 2.89E-06 | 1.52E-05 |
| TNFSF11 | mRNA | -2.3412 | 2.93E-06 | 1.54E-05 |
| C20orf204 | mRNA | 2.570373 | 2.94E-06 | 1.55E-05 |
| FAM151A | mRNA | -3.36889 | 2.96E-06 | 1.56E-05 |
| SLC10A1 | mRNA | -3.24941 | 2.98E-06 | 1.56E-05 |
| KLK10 | mRNA | -3.11191 | 2.98E-06 | 1.57E-05 |
| HPX | mRNA | -2.11481 | 3.02E-06 | 1.59E-05 |
| GJB3 | mRNA | -2.77441 | 3.05E-06 | 1.60E-05 |
| ANKFN1 | mRNA | 5.51062 | 3.07E-06 | 1.61E-05 |
| SLC39A5 | mRNA | -2.93082 | 3.18E-06 | 1.66E-05 |
| IGFBP1 | mRNA | -2.28494 | 3.19E-06 | 1.66E-05 |
| SPINT2 | mRNA | -2.03757 | 3.19E-06 | 1.66E-05 |
| GAGE2A | mRNA | 3.69604 | 3.20E-06 | 1.66E-05 |
| FAM9B | mRNA | -2.47033 | 3.26E-06 | 1.69E-05 |
| AC136428.1 | mRNA | -2.86438 | 3.38E-06 | 1.74E-05 |
| GABRA3 | mRNA | 5.340003 | 3.38E-06 | 1.74E-05 |
| FCRL3 | mRNA | -2.07572 | 3.39E-06 | 1.75E-05 |
| LYPD6B | mRNA | -3.14155 | 3.41E-06 | 1.76E-05 |
| RHOXF2 | mRNA | 3.530158 | 3.53E-06 | 1.82E-05 |
| MAGEA8 | mRNA | 4.366068 | 3.57E-06 | 1.83E-05 |
| GNRH2 | mRNA | -2.74859 | 3.60E-06 | 1.84E-05 |
| RGS20 | mRNA | 2.49593 | 3.62E-06 | 1.85E-05 |
| HPDL | mRNA | 2.140511 | 3.64E-06 | 1.86E-05 |
| GSC | mRNA | 2.837119 | 3.68E-06 | 1.88E-05 |
| PTPRS | mRNA | -2.04889 | 3.77E-06 | 1.92E-05 |
| LRRN4 | mRNA | -2.37266 | 3.88E-06 | 1.97E-05 |
| CALML3 | mRNA | -2.466 | 3.95E-06 | 2.00E-05 |
| TTR | mRNA | -2.64919 | 4.00E-06 | 2.02E-05 |
| SLCO6A1 | mRNA | 4.100535 | 4.08E-06 | 2.06E-05 |
| SAA2-SAA4 | mRNA | -3.99806 | 4.12E-06 | 2.07E-05 |
| DTHD1 | mRNA | -2.12659 | 4.14E-06 | 2.08E-05 |
| CLEC4D | mRNA | -2.19763 | 4.21E-06 | 2.11E-05 |
| TMPRSS15 | mRNA | 4.34006 | 4.45E-06 | 2.22E-05 |
| KRT20 | mRNA | 4.851735 | 4.48E-06 | 2.23E-05 |
| NEB | mRNA | 2.107238 | 4.49E-06 | 2.24E-05 |
| LPAR1 | mRNA | -2.04403 | 4.56E-06 | 2.27E-05 |
| DLX1 | mRNA | 2.42931 | 4.74E-06 | 2.35E-05 |
| WNT9A | mRNA | -2.45262 | 4.78E-06 | 2.37E-05 |
| CELA3A | mRNA | 3.069631 | 4.97E-06 | 2.45E-05 |
| FER1L6 | mRNA | 3.348479 | 4.99E-06 | 2.46E-05 |
| IL5RA | mRNA | -2.03861 | 5.00E-06 | 2.46E-05 |
| HIST1H1B | mRNA | 2.270832 | 5.03E-06 | 2.48E-05 |
| AKR1B15 | mRNA | 3.780966 | 5.12E-06 | 2.52E-05 |
| AKR7A3 | mRNA | -2.53049 | 5.21E-06 | 2.56E-05 |
| EFCAB1 | mRNA | -2.05036 | 5.24E-06 | 2.57E-05 |
| CST1 | mRNA | 4.766109 | 5.25E-06 | 2.57E-05 |
| A4GNT | mRNA | -2.63205 | 5.28E-06 | 2.58E-05 |
| NUGGC | mRNA | -2.1129 | 5.28E-06 | 2.58E-05 |
| SCGB3A1 | mRNA | -2.00503 | 5.34E-06 | 2.61E-05 |
| CADM3 | mRNA | -2.53049 | 5.43E-06 | 2.65E-05 |
| NTRK2 | mRNA | -2.36096 | 5.56E-06 | 2.71E-05 |
| KCNU1 | mRNA | 4.886455 | 5.73E-06 | 2.78E-05 |
| UGT2B10 | mRNA | -2.0439 | 5.87E-06 | 2.84E-05 |
| WNT7B | mRNA | -3.21388 | 5.94E-06 | 2.87E-05 |
| CLRN3 | mRNA | -2.91397 | 5.95E-06 | 2.88E-05 |
| CHRNA4 | mRNA | -3.81026 | 5.97E-06 | 2.88E-05 |
| PCDHAC1 | mRNA | -2.54751 | 6.08E-06 | 2.93E-05 |
| MYBPC1 | mRNA | 2.242922 | 6.29E-06 | 3.02E-05 |
| ELAVL2 | mRNA | 2.217706 | 6.33E-06 | 3.04E-05 |
| UNC5A | mRNA | 2.017933 | 6.38E-06 | 3.06E-05 |
| ARSF | mRNA | -3.5837 | 6.46E-06 | 3.09E-05 |
| SFTA2 | mRNA | -2.58861 | 6.49E-06 | 3.10E-05 |
| SLC3A1 | mRNA | -3.31127 | 6.56E-06 | 3.13E-05 |
| HGFAC | mRNA | -4.7279 | 6.67E-06 | 3.18E-05 |
| LAMC2 | mRNA | -2.08788 | 6.76E-06 | 3.21E-05 |
| MACC1 | mRNA | -2.12122 | 6.84E-06 | 3.24E-05 |
| CYP2A6 | mRNA | -4.57794 | 6.99E-06 | 3.30E-05 |
| PRDM7 | mRNA | 2.73067 | 7.07E-06 | 3.34E-05 |
| SIX4 | mRNA | 2.535978 | 7.11E-06 | 3.35E-05 |
| OR8G5 | mRNA | 3.787366 | 7.13E-06 | 3.36E-05 |
| BHMT | mRNA | -2.75514 | 7.13E-06 | 3.36E-05 |
| RGS7BP | mRNA | -2.16191 | 7.19E-06 | 3.38E-05 |
| PNOC | mRNA | -2.41808 | 7.20E-06 | 3.39E-05 |
| KIAA1644 | mRNA | -2.37069 | 7.31E-06 | 3.43E-05 |
| PEG3 | mRNA | -2.34209 | 7.43E-06 | 3.48E-05 |
| GLOD5 | mRNA | -2.18278 | 7.43E-06 | 3.48E-05 |
| TMEM132D | mRNA | -2.13763 | 7.44E-06 | 3.48E-05 |
| GCM1 | mRNA | 2.479156 | 7.50E-06 | 3.51E-05 |
| SLC34A2 | mRNA | -3.77725 | 7.57E-06 | 3.54E-05 |
| CYP2A7 | mRNA | -5.06651 | 7.60E-06 | 3.55E-05 |
| DRP2 | mRNA | 2.771402 | 7.66E-06 | 3.57E-05 |
| PLEKHB1 | mRNA | -2.2449 | 7.87E-06 | 3.66E-05 |
| NR1I2 | mRNA | -2.26688 | 8.05E-06 | 3.74E-05 |
| FABP1 | mRNA | -2.74654 | 8.14E-06 | 3.78E-05 |
| UBD | mRNA | 2.551114 | 8.30E-06 | 3.84E-05 |
| RTBDN | mRNA | 3.679289 | 8.46E-06 | 3.90E-05 |
| MZB1 | mRNA | -2.85834 | 8.62E-06 | 3.97E-05 |
| ACTL8 | mRNA | 3.998687 | 8.62E-06 | 3.97E-05 |
| VIPR2 | mRNA | -2.00507 | 8.71E-06 | 4.00E-05 |
| ALG1L | mRNA | 2.594427 | 8.76E-06 | 4.02E-05 |
| SPON1 | mRNA | -2.01915 | 8.92E-06 | 4.08E-05 |
| APOA1 | mRNA | -2.28401 | 9.01E-06 | 4.13E-05 |
| TNFRSF13B | mRNA | -2.22993 | 9.07E-06 | 4.14E-05 |
| CCDC144NL | mRNA | 3.432348 | 9.14E-06 | 4.17E-05 |
| SAA1 | mRNA | -3.92736 | 9.33E-06 | 4.25E-05 |
| CFHR3 | mRNA | -2.62964 | 9.39E-06 | 4.27E-05 |
| MPO | mRNA | -2.0353 | 9.56E-06 | 4.34E-05 |
| PAGE2 | mRNA | 6.035665 | 9.60E-06 | 4.35E-05 |
| ZNF99 | mRNA | 3.075185 | 1.02E-05 | 4.59E-05 |
| XCR1 | mRNA | -2.06439 | 1.02E-05 | 4.59E-05 |
| DSCAML1 | mRNA | -2.2471 | 1.04E-05 | 4.68E-05 |
| HCAR2 | mRNA | -2.03003 | 1.04E-05 | 4.69E-05 |
| UNC13A | mRNA | 2.060309 | 1.06E-05 | 4.73E-05 |
| LGSN | mRNA | -2.23932 | 1.07E-05 | 4.77E-05 |
| SEC14L3 | mRNA | -2.53222 | 1.11E-05 | 4.96E-05 |
| CLEC4C | mRNA | -2.24421 | 1.11E-05 | 4.96E-05 |
| BDKRB1 | mRNA | 2.115584 | 1.13E-05 | 5.03E-05 |
| MUC15 | mRNA | 3.806384 | 1.14E-05 | 5.05E-05 |
| ADARB2 | mRNA | -2.11265 | 1.15E-05 | 5.09E-05 |
| ZAN | mRNA | -2.02101 | 1.16E-05 | 5.15E-05 |
| SULT2A1 | mRNA | -2.03038 | 1.23E-05 | 5.41E-05 |
| GBP7 | mRNA | -2.70659 | 1.25E-05 | 5.51E-05 |
| BHLHE22 | mRNA | -2.2343 | 1.25E-05 | 5.51E-05 |
| KCNE1B | mRNA | -2.23319 | 1.25E-05 | 5.51E-05 |
| KCNH7 | mRNA | -2.46036 | 1.26E-05 | 5.52E-05 |
| OMD | mRNA | -2.41927 | 1.29E-05 | 5.64E-05 |
| CSDC2 | mRNA | -2.1477 | 1.29E-05 | 5.65E-05 |
| GCGR | mRNA | -3.94955 | 1.30E-05 | 5.69E-05 |
| ANO3 | mRNA | -2.39219 | 1.36E-05 | 5.91E-05 |
| CASKIN1 | mRNA | 2.153622 | 1.39E-05 | 6.01E-05 |
| AGR3 | mRNA | -2.74844 | 1.48E-05 | 6.36E-05 |
| HAS2 | mRNA | -2.35553 | 1.49E-05 | 6.41E-05 |
| C19orf67 | mRNA | 2.320609 | 1.50E-05 | 6.44E-05 |
| SOX11 | mRNA | 2.695966 | 1.53E-05 | 6.56E-05 |
| ACADL | mRNA | -2.77601 | 1.53E-05 | 6.56E-05 |
| WNT7A | mRNA | -3.02294 | 1.53E-05 | 6.57E-05 |
| TMEM252 | mRNA | -2.5461 | 1.57E-05 | 6.71E-05 |
| PKHD1L1 | mRNA | -2.15595 | 1.60E-05 | 6.81E-05 |
| CABP1 | mRNA | 2.00155 | 1.60E-05 | 6.82E-05 |
| CCK | mRNA | -2.34509 | 1.65E-05 | 7.02E-05 |
| SCRT1 | mRNA | 2.184275 | 1.70E-05 | 7.18E-05 |
| VAX2 | mRNA | 2.278025 | 1.70E-05 | 7.18E-05 |
| FCAMR | mRNA | -2.58157 | 1.72E-05 | 7.29E-05 |
| PADI3 | mRNA | 3.635722 | 1.76E-05 | 7.42E-05 |
| PNLIP | mRNA | 2.967199 | 1.78E-05 | 7.47E-05 |
| HPGD | mRNA | -3.36119 | 1.80E-05 | 7.55E-05 |
| DRGX | mRNA | 3.32058 | 1.82E-05 | 7.64E-05 |
| SMC1B | mRNA | 2.047624 | 1.83E-05 | 7.68E-05 |
| GATA5 | mRNA | -2.99013 | 1.84E-05 | 7.72E-05 |
| LIPN | mRNA | -2.30104 | 1.86E-05 | 7.78E-05 |
| UBL4B | mRNA | 3.460839 | 1.87E-05 | 7.83E-05 |
| AMN | mRNA | -2.04279 | 1.89E-05 | 7.88E-05 |
| SLC6A20 | mRNA | -2.69618 | 1.91E-05 | 7.97E-05 |
| FETUB | mRNA | -2.71558 | 1.99E-05 | 8.25E-05 |
| TMEM179 | mRNA | -3.27056 | 1.99E-05 | 8.27E-05 |
| WNT11 | mRNA | -2.4042 | 2.02E-05 | 8.35E-05 |
| CD207 | mRNA | -2.07639 | 2.07E-05 | 8.53E-05 |
| NSUN7 | mRNA | -2.25803 | 2.13E-05 | 8.75E-05 |
| CACNA1E | mRNA | 2.503425 | 2.13E-05 | 8.76E-05 |
| AFF2 | mRNA | 2.389558 | 2.14E-05 | 8.80E-05 |
| SDS | mRNA | -3.60536 | 2.15E-05 | 8.84E-05 |
| ALDOB | mRNA | -2.58658 | 2.16E-05 | 8.86E-05 |
| TMEM151B | mRNA | 2.008995 | 2.20E-05 | 8.98E-05 |
| IP6K3 | mRNA | -3.59145 | 2.22E-05 | 9.05E-05 |
| TENM1 | mRNA | -2.85168 | 2.22E-05 | 9.05E-05 |
| HTR1F | mRNA | 2.1917 | 2.28E-05 | 9.27E-05 |
| PLEK2 | mRNA | -2.18323 | 2.28E-05 | 9.28E-05 |
| GREM2 | mRNA | -4.03344 | 2.32E-05 | 9.43E-05 |
| MROH2A | mRNA | -3.03147 | 2.45E-05 | 9.91E-05 |
| WDR38 | mRNA | 2.478398 | 2.50E-05 | 0.000101 |
| HOXC6 | mRNA | 3.086121 | 2.51E-05 | 0.000101 |
| SULT1E1 | mRNA | -2.65202 | 2.53E-05 | 0.000102 |
| GP5 | mRNA | -2.01529 | 2.64E-05 | 0.000106 |
| LAMA2 | mRNA | -2.05602 | 2.65E-05 | 0.000106 |
| MATN3 | mRNA | 2.040745 | 2.70E-05 | 0.000108 |
| PRDM9 | mRNA | 2.711904 | 2.72E-05 | 0.000109 |
| ANGPTL1 | mRNA | -2.19301 | 2.78E-05 | 0.000111 |
| OAT | mRNA | -2.06454 | 2.78E-05 | 0.000111 |
| MMP12 | mRNA | 2.682138 | 2.87E-05 | 0.000114 |
| CHRDL1 | mRNA | -3.14183 | 2.89E-05 | 0.000115 |
| DLX6 | mRNA | 4.176925 | 2.94E-05 | 0.000116 |
| GLYATL3 | mRNA | -2.41009 | 2.95E-05 | 0.000117 |
| CNTN3 | mRNA | -3.25355 | 2.99E-05 | 0.000118 |
| KCNJ9 | mRNA | 2.314801 | 2.99E-05 | 0.000118 |
| HOXA6 | mRNA | 2.667285 | 2.99E-05 | 0.000118 |
| ZNF536 | mRNA | -2.37275 | 3.00E-05 | 0.000119 |
| PRAME | mRNA | 4.35165 | 3.10E-05 | 0.000122 |
| PPP1R14D | mRNA | 3.037478 | 3.11E-05 | 0.000122 |
| FAM196B | mRNA | -2.05371 | 3.19E-05 | 0.000125 |
| PRSS12 | mRNA | -2.10785 | 3.21E-05 | 0.000126 |
| SYT7 | mRNA | -2.19168 | 3.26E-05 | 0.000128 |
| CACNB4 | mRNA | 2.00648 | 3.36E-05 | 0.000131 |
| CYP8B1 | mRNA | -3.06691 | 3.39E-05 | 0.000132 |
| MAJIN | mRNA | -2.12295 | 3.44E-05 | 0.000134 |
| SMKR1 | mRNA | 2.052024 | 3.46E-05 | 0.000135 |
| IL17B | mRNA | 2.064226 | 3.61E-05 | 0.00014 |
| HIST1H2BF | mRNA | 2.472331 | 3.68E-05 | 0.000143 |
| WNK4 | mRNA | 2.059351 | 3.74E-05 | 0.000145 |
| GLT1D1 | mRNA | -2.15482 | 3.75E-05 | 0.000145 |
| CHRND | mRNA | 3.197243 | 3.83E-05 | 0.000148 |
| XKR4 | mRNA | -2.14774 | 3.84E-05 | 0.000148 |
| SPOCK3 | mRNA | -3.31136 | 3.85E-05 | 0.000149 |
| NQO1 | mRNA | 2.467041 | 4.01E-05 | 0.000154 |
| ACER1 | mRNA | -2.18034 | 4.03E-05 | 0.000154 |
| TFF2 | mRNA | -3.10758 | 4.07E-05 | 0.000156 |
| DPP10 | mRNA | -2.94787 | 4.14E-05 | 0.000158 |
| TAT | mRNA | -2.61657 | 4.18E-05 | 0.00016 |
| SERTM2 | mRNA | -4.19354 | 4.22E-05 | 0.000161 |
| BRSK2 | mRNA | 2.120576 | 4.24E-05 | 0.000161 |
| IRX5 | mRNA | 2.500346 | 4.24E-05 | 0.000161 |
| KCNG3 | mRNA | 2.931791 | 4.28E-05 | 0.000163 |
| LKAAEAR1 | mRNA | 3.02671 | 4.31E-05 | 0.000164 |
| PRSS2 | mRNA | 3.029885 | 4.37E-05 | 0.000166 |
| STRA8 | mRNA | 2.577542 | 4.38E-05 | 0.000166 |
| CTSV | mRNA | 2.046452 | 4.38E-05 | 0.000166 |
| TREH | mRNA | -2.08303 | 4.49E-05 | 0.00017 |
| C1QL1 | mRNA | 2.158084 | 4.67E-05 | 0.000176 |
| LRRTM4 | mRNA | -2.73233 | 4.97E-05 | 0.000186 |
| RPL10L | mRNA | 2.708657 | 5.05E-05 | 0.000189 |
| HAVCR1 | mRNA | 2.831397 | 5.13E-05 | 0.000191 |
| CLEC17A | mRNA | -2.04196 | 5.29E-05 | 0.000197 |
| B3GALT1 | mRNA | 2.689759 | 5.36E-05 | 0.000199 |
| NPC1L1 | mRNA | -3.43683 | 5.46E-05 | 0.000202 |
| CAPN6 | mRNA | -2.90404 | 5.58E-05 | 0.000206 |
| TM4SF20 | mRNA | 3.853949 | 5.84E-05 | 0.000215 |
| SLC4A1 | mRNA | -2.31889 | 5.84E-05 | 0.000215 |
| ARL14 | mRNA | -2.16335 | 6.13E-05 | 0.000224 |
| C1QL4 | mRNA | 2.510653 | 6.34E-05 | 0.000231 |
| COCH | mRNA | 2.422344 | 6.54E-05 | 0.000237 |
| LMO1 | mRNA | 2.645383 | 7.16E-05 | 0.000256 |
| DMBX1 | mRNA | 2.860005 | 7.22E-05 | 0.000258 |
| ERVV-2 | mRNA | 3.11627 | 7.24E-05 | 0.000259 |
| DCAF12L2 | mRNA | 2.227735 | 7.32E-05 | 0.000261 |
| PAGE1 | mRNA | 5.089087 | 7.33E-05 | 0.000262 |
| DLX2 | mRNA | 2.303675 | 7.76E-05 | 0.000275 |
| PPP2R2C | mRNA | 3.982122 | 7.83E-05 | 0.000277 |
| EREG | mRNA | -2.27904 | 7.86E-05 | 0.000278 |
| CYP19A1 | mRNA | 2.634908 | 7.93E-05 | 0.00028 |
| REG3A | mRNA | 6.116613 | 8.03E-05 | 0.000283 |
| TDRD15 | mRNA | -2.37418 | 8.04E-05 | 0.000283 |
| COX6A2 | mRNA | -2.94598 | 8.10E-05 | 0.000285 |
| ZPLD1 | mRNA | -2.18748 | 8.19E-05 | 0.000288 |
| RDH8 | mRNA | 2.838583 | 8.36E-05 | 0.000293 |
| BPIFB4 | mRNA | 2.679913 | 8.43E-05 | 0.000295 |
| SMIM24 | mRNA | -3.20127 | 8.45E-05 | 0.000295 |
| SPERT | mRNA | 2.747468 | 8.54E-05 | 0.000298 |
| ASXL3 | mRNA | -2.39901 | 8.68E-05 | 0.000302 |
| RBBP8NL | mRNA | -2.29966 | 8.73E-05 | 0.000304 |
| CSF3 | mRNA | -2.35879 | 8.77E-05 | 0.000305 |
| UGT2B11 | mRNA | 2.071275 | 8.81E-05 | 0.000306 |
| OR56A3 | mRNA | 2.835447 | 8.83E-05 | 0.000307 |
| GFY | mRNA | 2.579283 | 8.97E-05 | 0.000311 |
| CLEC2L | mRNA | 3.425017 | 9.15E-05 | 0.000317 |
| OR8A1 | mRNA | 2.771301 | 9.36E-05 | 0.000323 |
| GOLGA6B | mRNA | -2.45949 | 9.44E-05 | 0.000325 |
| GNMT | mRNA | -2.30245 | 9.63E-05 | 0.000331 |
| CNTFR | mRNA | -2.97803 | 9.66E-05 | 0.000332 |
| CACNG1 | mRNA | 3.261045 | 9.74E-05 | 0.000334 |
| MAGEA11 | mRNA | 3.683902 | 0.000101 | 0.000344 |
| BRINP3 | mRNA | 3.366613 | 0.000104 | 0.000354 |
| XKR3 | mRNA | 3.15858 | 0.000104 | 0.000356 |
| CA4 | mRNA | 2.234698 | 0.000105 | 0.000357 |
| ACTN2 | mRNA | 2.399923 | 0.000107 | 0.000363 |
| CSAG2 | mRNA | 3.42943 | 0.000108 | 0.000367 |
| SLC22A6 | mRNA | 3.382976 | 0.000109 | 0.000371 |
| SI | mRNA | 2.529417 | 0.00011 | 0.000372 |
| ZDHHC19 | mRNA | -2.08438 | 0.00011 | 0.000373 |
| CDX2 | mRNA | 2.014162 | 0.000112 | 0.000378 |
| MMP8 | mRNA | -2.03042 | 0.000115 | 0.000387 |
| SLC30A3 | mRNA | 2.203799 | 0.000117 | 0.000392 |
| DIO2 | mRNA | 2.207739 | 0.000118 | 0.000395 |
| HTR1B | mRNA | 2.012085 | 0.000119 | 0.0004 |
| BSND | mRNA | 2.551849 | 0.00012 | 0.000402 |
| CXorf66 | mRNA | -2.11867 | 0.000123 | 0.00041 |
| ART5 | mRNA | -2.05902 | 0.000126 | 0.00042 |
| ARPP21 | mRNA | -2.3229 | 0.000126 | 0.000421 |
| TMEM88B | mRNA | 2.875955 | 0.000127 | 0.000421 |
| OSR2 | mRNA | 2.654375 | 0.000127 | 0.000423 |
| RNF17 | mRNA | 3.537714 | 0.000131 | 0.000436 |
| CNTNAP5 | mRNA | 2.5343 | 0.000131 | 0.000436 |
| SLC22A7 | mRNA | -2.08441 | 0.000133 | 0.00044 |
| AKR1B10 | mRNA | 3.341052 | 0.000134 | 0.000443 |
| GAD1 | mRNA | 2.895131 | 0.000134 | 0.000443 |
| PRSS8 | mRNA | -3.52568 | 0.000134 | 0.000444 |
| C1orf61 | mRNA | 2.143101 | 0.000136 | 0.00045 |
| DNER | mRNA | 3.252359 | 0.000138 | 0.000456 |
| CCNO | mRNA | 2.033471 | 0.00014 | 0.000459 |
| CDC20B | mRNA | 2.110171 | 0.000141 | 0.000464 |
| DYDC1 | mRNA | 2.264673 | 0.000142 | 0.000465 |
| PTPRD | mRNA | -2.06636 | 0.000143 | 0.000469 |
| CDH9 | mRNA | 3.138003 | 0.000143 | 0.00047 |
| KCNE5 | mRNA | 2.085239 | 0.000144 | 0.000471 |
| RPRML | mRNA | 2.491588 | 0.000144 | 0.000472 |
| ISLR | mRNA | -2.12432 | 0.000147 | 0.00048 |
| SCGB1A1 | mRNA | 2.764647 | 0.00015 | 0.000488 |
| PRSS56 | mRNA | 4.000482 | 0.000155 | 0.000503 |
| ACE2 | mRNA | -2.26488 | 0.000155 | 0.000506 |
| HOXB13 | mRNA | 3.75083 | 0.000156 | 0.000508 |
| KRT12 | mRNA | 4.183738 | 0.000159 | 0.000515 |
| HAPLN1 | mRNA | 2.507507 | 0.000161 | 0.000522 |
| PPP1R1A | mRNA | -2.32599 | 0.000165 | 0.000533 |
| PI15 | mRNA | 2.432031 | 0.000167 | 0.000539 |
| HDGFL1 | mRNA | 3.653416 | 0.000168 | 0.000541 |
| ARG1 | mRNA | -2.07049 | 0.00017 | 0.000546 |
| RND2 | mRNA | -2.20193 | 0.00017 | 0.000548 |
| TLX2 | mRNA | 2.024834 | 0.000173 | 0.000556 |
| STRA6 | mRNA | 2.232428 | 0.000173 | 0.000556 |
| FABP6 | mRNA | 2.383328 | 0.000177 | 0.000568 |
| LUZP2 | mRNA | 2.070632 | 0.000178 | 0.00057 |
| CHP2 | mRNA | 4.632586 | 0.000183 | 0.000582 |
| LRRTM1 | mRNA | -2.30065 | 0.000188 | 0.000598 |
| PRAMEF9 | mRNA | 2.75347 | 0.000189 | 0.000599 |
| KCNH5 | mRNA | 2.901594 | 0.000198 | 0.000626 |
| GHRHR | mRNA | 2.825898 | 0.000201 | 0.000635 |
| HSD3B1 | mRNA | -2.38592 | 0.000205 | 0.000645 |
| ODAM | mRNA | 4.530866 | 0.00021 | 0.000658 |
| HSD11B1 | mRNA | -2.43711 | 0.000211 | 0.000661 |
| LUM | mRNA | -2.19049 | 0.000212 | 0.000664 |
| KRTAP1-1 | mRNA | -2.69555 | 0.000212 | 0.000665 |
| ATRNL1 | mRNA | -2.25755 | 0.000212 | 0.000665 |
| KRT83 | mRNA | -2.00116 | 0.000213 | 0.000667 |
| CXCR5 | mRNA | -2.03635 | 0.000216 | 0.000675 |
| NALCN | mRNA | -2.11025 | 0.000222 | 0.000692 |
| DIO3 | mRNA | -2.7253 | 0.000223 | 0.000695 |
| CLPS | mRNA | 2.474101 | 0.000226 | 0.000703 |
| GABRB3 | mRNA | -2.92043 | 0.000227 | 0.000705 |
| CYP2E1 | mRNA | -3.62585 | 0.000231 | 0.000717 |
| TNP1 | mRNA | 4.044006 | 0.000239 | 0.000738 |
| TMEM78 | mRNA | 2.056117 | 0.00024 | 0.00074 |
| CRP | mRNA | -3.04042 | 0.000248 | 0.000764 |
| HIST3H2BB | mRNA | 2.413662 | 0.000255 | 0.000782 |
| NPW | mRNA | -2.5137 | 0.000259 | 0.000793 |
| DUSP13 | mRNA | 3.284394 | 0.000259 | 0.000794 |
| DEFA4 | mRNA | -2.19717 | 0.000263 | 0.000803 |
| UGT1A4 | mRNA | -2.3264 | 0.000264 | 0.000806 |
| KCNJ13 | mRNA | -2.4356 | 0.000266 | 0.000811 |
| GCK | mRNA | -3.35476 | 0.00027 | 0.000824 |
| LRP2 | mRNA | -2.20484 | 0.000274 | 0.000835 |
| UPP2 | mRNA | -2.22825 | 0.000276 | 0.00084 |
| NR0B1 | mRNA | 4.133969 | 0.000284 | 0.000861 |
| FSTL5 | mRNA | 3.924249 | 0.00029 | 0.000877 |
| TRIM54 | mRNA | 2.275665 | 0.00029 | 0.000879 |
| SEZ6 | mRNA | 2.481404 | 0.0003 | 0.000903 |
| C19orf81 | mRNA | 2.956091 | 0.000306 | 0.000919 |
| APOA4 | mRNA | -2.89025 | 0.000308 | 0.000925 |
| KRT80 | mRNA | -2.1506 | 0.000314 | 0.000942 |
| SBK3 | mRNA | 2.097899 | 0.000317 | 0.000949 |
| COL2A1 | mRNA | 3.320165 | 0.000323 | 0.000963 |
| WISP2 | mRNA | -2.41281 | 0.000323 | 0.000964 |
| ABCB11 | mRNA | -2.27436 | 0.000332 | 0.00099 |
| SERPINI2 | mRNA | 2.162298 | 0.000335 | 0.000996 |
| HIST1H3G | mRNA | 2.32211 | 0.000351 | 0.001039 |
| PRG4 | mRNA | -2.2384 | 0.000352 | 0.00104 |
| SPINK4 | mRNA | 2.149687 | 0.000362 | 0.001066 |
| PANX3 | mRNA | 2.404771 | 0.000366 | 0.001078 |
| TEDDM1 | mRNA | 2.669555 | 0.000373 | 0.001096 |
| CPZ | mRNA | -2.01961 | 0.000374 | 0.001098 |
| PCDHA12 | mRNA | -2.34122 | 0.000384 | 0.001123 |
| NRCAM | mRNA | 2.190816 | 0.000406 | 0.001181 |
| CXCL1 | mRNA | -2.10866 | 0.000412 | 0.001195 |
| ANGPTL7 | mRNA | -2.27264 | 0.000419 | 0.001212 |
| MEP1A | mRNA | 2.54124 | 0.000429 | 0.001238 |
| STAC | mRNA | -2.05375 | 0.000432 | 0.001247 |
| ZIC4 | mRNA | 2.808279 | 0.000432 | 0.001247 |
| ZCCHC12 | mRNA | 2.366182 | 0.000438 | 0.001261 |
| GNG4 | mRNA | 2.989208 | 0.000442 | 0.00127 |
| GFRA1 | mRNA | -2.1843 | 0.000467 | 0.001335 |
| ALPI | mRNA | 2.445761 | 0.000475 | 0.001356 |
| KRT4 | mRNA | -2.28202 | 0.000488 | 0.001386 |
| CPS1 | mRNA | -2.23381 | 0.00049 | 0.001391 |
| LECT2 | mRNA | -2.34914 | 0.000491 | 0.001394 |
| TAS2R60 | mRNA | -2.00752 | 0.000497 | 0.001408 |
| CHRNA3 | mRNA | 2.104423 | 0.000497 | 0.001408 |
| TYRP1 | mRNA | 2.46586 | 0.000506 | 0.00143 |
| KISS1R | mRNA | 2.982776 | 0.000506 | 0.00143 |
| KCNK12 | mRNA | 2.13853 | 0.00051 | 0.001439 |
| OLIG3 | mRNA | 2.950191 | 0.000537 | 0.001506 |
| SOHLH1 | mRNA | 2.538164 | 0.000546 | 0.001529 |
| MUSK | mRNA | -2.01215 | 0.000552 | 0.001543 |
| CYP1A1 | mRNA | -3.26326 | 0.000558 | 0.001558 |
| AC013470.2 | mRNA | 2.138676 | 0.000561 | 0.001565 |
| GIP | mRNA | 2.887204 | 0.000567 | 0.001579 |
| CCL25 | mRNA | 2.269166 | 0.00057 | 0.001586 |
| PAGE4 | mRNA | 3.798897 | 0.000572 | 0.001593 |
| PRSS16 | mRNA | -2.28684 | 0.000573 | 0.001595 |
| KRTAP4-1 | mRNA | 2.196006 | 0.000574 | 0.001598 |
| TAC1 | mRNA | -2.19239 | 0.000575 | 0.001599 |
| HOXD13 | mRNA | 2.710141 | 0.00061 | 0.001686 |
| GALNT17 | mRNA | -2.29586 | 0.000616 | 0.0017 |
| IGDCC3 | mRNA | 2.556371 | 0.000617 | 0.001702 |
| SLCO1B7 | mRNA | -2.26772 | 0.000621 | 0.001709 |
| LCN2 | mRNA | 2.179881 | 0.000628 | 0.001727 |
| KIRREL2 | mRNA | 2.025757 | 0.000639 | 0.001751 |
| CIB3 | mRNA | 2.21027 | 0.000657 | 0.001795 |
| CTNNA2 | mRNA | 3.2751 | 0.000678 | 0.001845 |
| HRG | mRNA | -2.29979 | 0.000684 | 0.001858 |
| KCNK15 | mRNA | -2.01665 | 0.000686 | 0.001864 |
| PCLO | mRNA | -2.43657 | 0.00069 | 0.001872 |
| TLX3 | mRNA | 2.974346 | 0.000705 | 0.001909 |
| KCNF1 | mRNA | 2.530563 | 0.000711 | 0.001924 |
| MCCD1 | mRNA | 2.918117 | 0.000714 | 0.001932 |
| ZNF728 | mRNA | 2.40482 | 0.000728 | 0.001965 |
| AMBN | mRNA | 3.373143 | 0.000743 | 0.002001 |
| HAL | mRNA | -2.34685 | 0.000755 | 0.002033 |
| RELN | mRNA | -2.53757 | 0.000779 | 0.00209 |
| RIMBP2 | mRNA | -2.37289 | 0.000828 | 0.00221 |
| HCN1 | mRNA | -2.20947 | 0.000845 | 0.002248 |
| SLC13A2 | mRNA | -2.00731 | 0.000858 | 0.00228 |
| REG1B | mRNA | 3.793331 | 0.000861 | 0.002288 |
| CELA3B | mRNA | 2.023 | 0.000867 | 0.002301 |
| FGFR2 | mRNA | -2.13199 | 0.000894 | 0.002362 |
| ASPHD1 | mRNA | 2.164249 | 0.000904 | 0.002384 |
| ADAMTS20 | mRNA | 2.284596 | 0.00091 | 0.002399 |
| GRPR | mRNA | 2.415605 | 0.00095 | 0.00249 |
| CRABP1 | mRNA | 2.428877 | 0.00096 | 0.002513 |
| CNTNAP4 | mRNA | 2.684873 | 0.00097 | 0.002538 |
| HTR1D | mRNA | 2.597934 | 0.000983 | 0.002568 |
| GABRR3 | mRNA | 2.598449 | 0.000989 | 0.002583 |
| MYH8 | mRNA | 2.343731 | 0.001 | 0.002608 |
| MCIDAS | mRNA | 2.347854 | 0.001002 | 0.002614 |
| CA9 | mRNA | -2.00965 | 0.001007 | 0.002624 |
| AL355102.2 | mRNA | 2.457908 | 0.001009 | 0.00263 |
| CAMKV | mRNA | 2.203826 | 0.001025 | 0.002666 |
| MME | mRNA | -2.76366 | 0.001047 | 0.002717 |
| CSAG3 | mRNA | 2.824004 | 0.001053 | 0.002729 |
| EN1 | mRNA | 3.544608 | 0.001082 | 0.002798 |
| C12orf56 | mRNA | 2.382911 | 0.001089 | 0.002814 |
| HBM | mRNA | -2.13797 | 0.001113 | 0.002868 |
| LRRC26 | mRNA | 2.493385 | 0.001117 | 0.002878 |
| CA10 | mRNA | 2.13756 | 0.001129 | 0.002906 |
| EGF | mRNA | 2.778829 | 0.001146 | 0.002942 |
| MYH4 | mRNA | 4.855218 | 0.001167 | 0.002991 |
| KRT7 | mRNA | -2.00054 | 0.001207 | 0.003081 |
| FRRS1L | mRNA | 2.161 | 0.001216 | 0.003098 |
| PNCK | mRNA | 2.877623 | 0.001228 | 0.003125 |
| C9orf57 | mRNA | 2.005438 | 0.001264 | 0.003206 |
| OVOL2 | mRNA | -2.06101 | 0.001281 | 0.003244 |
| FXYD2 | mRNA | -2.64876 | 0.001285 | 0.003253 |
| KCNH6 | mRNA | 2.271423 | 0.001366 | 0.003434 |
| SMR3A | mRNA | 5.013978 | 0.001371 | 0.003445 |
| CKMT1A | mRNA | 2.065023 | 0.001432 | 0.003575 |
| BCL2L10 | mRNA | -2.07231 | 0.001442 | 0.003596 |
| GRP | mRNA | 2.187645 | 0.001443 | 0.003598 |
| MAGEB6 | mRNA | 4.695835 | 0.001454 | 0.003624 |
| AKAP14 | mRNA | 2.07551 | 0.001469 | 0.003658 |
| EPCAM | mRNA | -2.86398 | 0.001494 | 0.003713 |
| SMPX | mRNA | 2.316289 | 0.00153 | 0.003794 |
| SRARP | mRNA | 2.616361 | 0.001532 | 0.003799 |
| HOXC13 | mRNA | 2.187918 | 0.001533 | 0.003799 |
| CLDN2 | mRNA | -2.17412 | 0.001553 | 0.00384 |
| KLK2 | mRNA | 2.587525 | 0.00158 | 0.003899 |
| ASB15 | mRNA | 2.403009 | 0.001597 | 0.003939 |
| PLA2G2A | mRNA | -2.60059 | 0.001617 | 0.003982 |
| PRAMEF8 | mRNA | 2.632391 | 0.001619 | 0.003987 |
| KCNJ6 | mRNA | 2.148054 | 0.001718 | 0.004204 |
| GLYATL1B | mRNA | 2.434795 | 0.001782 | 0.004345 |
| HMX1 | mRNA | 3.000392 | 0.001812 | 0.004407 |
| ZNF679 | mRNA | 2.526031 | 0.001814 | 0.004411 |
| GSTA2 | mRNA | -2.32864 | 0.001826 | 0.004437 |
| EEF1A2 | mRNA | 3.800204 | 0.001855 | 0.0045 |
| DNMT3L | mRNA | -2.22841 | 0.001915 | 0.004633 |
| WFDC2 | mRNA | -2.09089 | 0.001954 | 0.004713 |
| LUZP4 | mRNA | 4.210414 | 0.00198 | 0.004769 |
| CALY | mRNA | 2.231388 | 0.002047 | 0.004917 |
| MAGEB16 | mRNA | 4.533806 | 0.00205 | 0.004923 |
| MMP7 | mRNA | -2.40682 | 0.002052 | 0.004928 |
| ANKRD33 | mRNA | 2.36717 | 0.002069 | 0.004963 |
| ZNF560 | mRNA | 2.374817 | 0.002124 | 0.00508 |
| CHL1 | mRNA | -2.55286 | 0.002127 | 0.005084 |
| SOHLH2 | mRNA | 2.715704 | 0.002145 | 0.005123 |
| NTS | mRNA | -2.11454 | 0.002158 | 0.00515 |
| FAM178B | mRNA | 2.235897 | 0.002273 | 0.005397 |
| SLC22A12 | mRNA | 3.273499 | 0.00229 | 0.005435 |
| TEX37 | mRNA | 4.298221 | 0.002298 | 0.005451 |
| POPDC3 | mRNA | 2.786919 | 0.002338 | 0.005535 |
| BPIFA1 | mRNA | 2.642439 | 0.002353 | 0.005563 |
| ISM2 | mRNA | 2.758114 | 0.002355 | 0.005566 |
| PRAC2 | mRNA | 2.590046 | 0.002391 | 0.005642 |
| CXCL13 | mRNA | -2.41882 | 0.002397 | 0.005654 |
| TUBA3C | mRNA | 3.310727 | 0.002437 | 0.005737 |
| CXorf67 | mRNA | 2.550256 | 0.002452 | 0.005769 |
| CTNND2 | mRNA | -2.70061 | 0.002498 | 0.005862 |
| PRAMEF11 | mRNA | 2.44461 | 0.002514 | 0.005895 |
| KLK4 | mRNA | 2.624502 | 0.002522 | 0.005909 |
| EDDM3A | mRNA | 2.575249 | 0.002539 | 0.005943 |
| LCE2D | mRNA | -2.06908 | 0.002805 | 0.006496 |
| TRIM60 | mRNA | 2.056313 | 0.002898 | 0.006675 |
| MYH1 | mRNA | 2.446488 | 0.002983 | 0.006847 |
| AC115220.1 | mRNA | 4.012823 | 0.002995 | 0.006871 |
| TCN1 | mRNA | -2.32749 | 0.003026 | 0.006935 |
| PRAMEF4 | mRNA | 3.166299 | 0.003062 | 0.007004 |
| FTHL17 | mRNA | 3.759544 | 0.003181 | 0.007245 |
| PTGDS | mRNA | -2.4068 | 0.00319 | 0.007262 |
| SCGB1D2 | mRNA | 2.771941 | 0.003192 | 0.007265 |
| CA12 | mRNA | 2.244269 | 0.003203 | 0.007285 |
| CSN2 | mRNA | 3.830824 | 0.003213 | 0.007303 |
| MYH13 | mRNA | 2.125361 | 0.003317 | 0.007498 |
| TRHDE | mRNA | -2.45076 | 0.00337 | 0.007601 |
| HSPB3 | mRNA | 2.308733 | 0.003393 | 0.007646 |
| RLN3 | mRNA | 2.065784 | 0.003456 | 0.007773 |
| SOX14 | mRNA | 2.583581 | 0.003498 | 0.007859 |
| ALX1 | mRNA | 2.596083 | 0.003548 | 0.007956 |
| FDCSP | mRNA | 2.43252 | 0.003647 | 0.008155 |
| EMX1 | mRNA | 2.056755 | 0.003668 | 0.008195 |
| CCDC187 | mRNA | 2.287118 | 0.003668 | 0.008195 |
| PRSS1 | mRNA | 2.023296 | 0.00375 | 0.008361 |
| HNRNPCL3 | mRNA | 2.111726 | 0.003759 | 0.008377 |
| POU3F4 | mRNA | 2.26372 | 0.003813 | 0.008483 |
| RFX6 | mRNA | 2.177285 | 0.003988 | 0.008818 |
| TSPY2 | mRNA | 3.534089 | 0.00402 | 0.008878 |
| OTOG | mRNA | 2.440542 | 0.004041 | 0.008914 |
| AQP8 | mRNA | 2.044046 | 0.004073 | 0.008977 |
| UGT1A10 | mRNA | 2.341101 | 0.004125 | 0.009075 |
| TENM2 | mRNA | -2.06687 | 0.00413 | 0.009083 |
| DMKN | mRNA | -2.01269 | 0.004165 | 0.009157 |
| SPP1 | mRNA | 2.115749 | 0.004165 | 0.009157 |
| PRAMEF27 | mRNA | 3.416788 | 0.004285 | 0.00939 |
| CCDC185 | mRNA | 2.073412 | 0.004318 | 0.009457 |
| SMR3B | mRNA | 3.154809 | 0.004384 | 0.00959 |
| RSPO2 | mRNA | -2.11104 | 0.004457 | 0.009739 |
