# Supplementary material for: Construction and comprehensive analysis of a ceRNA network to reveal potential prognostic biomarkers for hepatocellular carcinoma
Source: Cancer Cell Int. 2019 Apr 11;19:90. doi: 10.1186/s12935-019-0817-y (PMC6458652; doi:10.1186/s12935-019-0817-y)
Supplement: Supplementary file 2 — Additional file 2: Table S2. Forty-four DElncRNAs interacted with nine DEmiRNAs retrieved from the miRcode database. [file 12935_2019_817_MOESM2_ESM.docx]

**Table S2. Forty-four DElncRNAs interacted with nine DEmiRNAs retrieved from the miRcode database.**

| **lncRNA** | **miRNA** |
| --- | --- |
| IGF2-AS | hsa-miR-214 |
| PART1 | hsa-miR-187 |
| PART1 | hsa-miR-429 |
| PART1 | hsa-miR-214 |
| PART1 | hsa-miR-508 |
| AL357153.1 | hsa-miR-183 |
| AL357153.1 | hsa-miR-187 |
| AL357153.1 | hsa-miR-383 |
| AP002478.1 | hsa-miR-182 |
| AP002478.1 | hsa-miR-184 |
| AP002478.1 | hsa-miR-508 |
| AC024563.1 | hsa-miR-183 |
| C17orf82 | hsa-miR-214 |
| LINC00221 | hsa-miR-96 |
| LINC00221 | hsa-miR-182 |
| LINC00221 | hsa-miR-508 |
| TCL6 | hsa-miR-96 |
| TCL6 | hsa-miR-182 |
| TCL6 | hsa-miR-183 |
| TCL6 | hsa-miR-187 |
| TCL6 | hsa-miR-214 |
| AC087392.1 | hsa-miR-429 |
| FAM99B | hsa-miR-214 |
| FAM99A | hsa-miR-214 |
| UCA1 | hsa-miR-96 |
| UCA1 | hsa-miR-182 |
| UCA1 | hsa-miR-184 |
| UCA1 | hsa-miR-214 |
| UCA1 | hsa-miR-383 |
| AL110292.1 | hsa-miR-429 |
| DSCR4-IT1 | hsa-miR-508 |
| LINC00114 | hsa-miR-96 |
| LINC00114 | hsa-miR-182 |
| LINC00114 | hsa-miR-429 |
| LINC00114 | hsa-miR-214 |
| LINC00114 | hsa-miR-508 |
| SFTA1P | hsa-miR-182 |
| PCA3 | hsa-miR-96 |
| PCA3 | hsa-miR-182 |
| PCA3 | hsa-miR-214 |
| PCA3 | hsa-miR-383 |
| HAR1A | hsa-miR-214 |
| LINC00351 | hsa-miR-214 |
| LINC00355 | hsa-miR-214 |
| C14orf144 | hsa-miR-96 |
| C14orf144 | hsa-miR-214 |
| SACS-AS1 | hsa-miR-187 |
| SACS-AS1 | hsa-miR-508 |
| ERVMER61-1 | hsa-miR-96 |
| ERVMER61-1 | hsa-miR-182 |
| DNM3OS | hsa-miR-214 |
| AL161645.1 | hsa-miR-96 |
| AL161645.1 | hsa-miR-184 |
| DLX6-AS1 | hsa-miR-429 |
| DLX6-AS1 | hsa-miR-214 |
| DLX6-AS1 | hsa-miR-383 |
| BPESC1 | hsa-miR-214 |
| BPESC1 | hsa-miR-508 |
| MYCNOS | hsa-miR-183 |
| MYCNOS | hsa-miR-214 |
| MAGI2-AS3 | hsa-miR-429 |
| MAGI2-AS3 | hsa-miR-214 |
| MAGI2-AS3 | hsa-miR-508 |
| RBMS3-AS3 | hsa-miR-96 |
| RBMS3-AS3 | hsa-miR-182 |
| CLRN1-AS1 | hsa-miR-429 |
| KLHL6-AS1 | hsa-miR-96 |
| AC073352.1 | hsa-miR-96 |
| AC073352.1 | hsa-miR-182 |
| HOTTIP | hsa-miR-184 |
| HOTTIP | hsa-miR-187 |
| HOTTIP | hsa-miR-214 |
| AL445228.2 | hsa-miR-383 |
| CRNDE | hsa-miR-183 |
| CRNDE | hsa-miR-508 |
| LINC00491 | hsa-miR-184 |
| LINC00491 | hsa-miR-429 |
| RMST | hsa-miR-96 |
| RMST | hsa-miR-182 |
| RMST | hsa-miR-429 |
| RMST | hsa-miR-214 |
| RMST | hsa-miR-508 |
| AL139385.1 | hsa-miR-183 |
| AP006285.1 | hsa-miR-214 |
| AP006285.1 | hsa-miR-508 |
| LINC00485 | hsa-miR-214 |
| LINC00485 | hsa-miR-383 |
| DIO3OS | hsa-miR-214 |
| DIO3OS | hsa-miR-508 |
| DIO3OS | hsa-miR-383 |
| LINC00519 | hsa-miR-214 |
| PWRN1 | hsa-miR-184 |
| PWRN1 | hsa-miR-508 |
