# Supplementary material for: Construction and comprehensive analysis of a ceRNA network to reveal potential prognostic biomarkers for hepatocellular carcinoma
Source: Cancer Cell Int. 2019 Apr 11;19:90. doi: 10.1186/s12935-019-0817-y (PMC6458652; doi:10.1186/s12935-019-0817-y)
Supplement: Supplementary file 3 — Additional file 3: Table S3. Four DEmiRNAs interacted with six DEmRNAs retrieved from the miRDB, miRTarBase and TargetScan databases. [file 12935_2019_817_MOESM3_ESM.docx]

**Table S3. Four DEmiRNAs interacted with six DEmRNAs retrieved from the miRDB, miRTarBase and TargetScan databases.**

| **DEmiRNA** | **DEmRNA** |
| --- | --- |
| hsa-miR-182 | THBS1 |
| hsa-miR-182 | CHL1 |
| hsa-miR-183 | CCNB1 |
| hsa-miR-429 | SHCBP1 |
| hsa-miR-96 | SLC1A1 |
| hsa-miR-96 | PROK2 |
