# Supplementary material for: Construction and comprehensive analysis of a ceRNA network to reveal potential prognostic biomarkers for hepatocellular carcinoma
Source: Cancer Cell Int. 2019 Apr 11;19:90. doi: 10.1186/s12935-019-0817-y (PMC6458652; doi:10.1186/s12935-019-0817-y)
Supplement: Supplementary file 4 — Additional file 4: Table S4. Interactions of the ceRNA network in HCC. [file 12935_2019_817_MOESM4_ESM.docx]

**Table S4. Interactions of the ceRNA network in HCC.**

| **lncRNA** | **miRNA** | **mRNA** |
| --- | --- | --- |
| PART1 | hsa-miR-429 | SHCBP1 |
| AL357153.1 | hsa-miR-183 | CCNB1 |
| AP002478.1 | hsa-miR-182 | THBS1 |
| AC024563.1 | hsa-miR-183 | CCNB1 |
| LINC00221 | hsa-miR-96 | SLC1A1 |
| LINC00221 | hsa-miR-182 | THBS1 |
| TCL6 | hsa-miR-96 | SLC1A1 |
| TCL6 | hsa-miR-182 | THBS1 |
| TCL6 | hsa-miR-183 | CCNB1 |
| AC087392.1 | hsa-miR-429 | SHCBP1 |
| UCA1 | hsa-miR-96 | SLC1A1 |
| UCA1 | hsa-miR-182 | THBS1 |
| AL110292.1 | hsa-miR-429 | SHCBP1 |
| LINC00114 | hsa-miR-96 | SLC1A1 |
| LINC00114 | hsa-miR-182 | THBS1 |
| LINC00114 | hsa-miR-429 | SHCBP1 |
| SFTA1P | hsa-miR-182 | THBS1 |
| PCA3 | hsa-miR-96 | SLC1A1 |
| PCA3 | hsa-miR-182 | THBS1 |
| C14orf144 | hsa-miR-96 | SLC1A1 |
| ERVMER61-1 | hsa-miR-96 | SLC1A1 |
| ERVMER61-1 | hsa-miR-182 | THBS1 |
| AL161645.1 | hsa-miR-96 | SLC1A1 |
| DLX6-AS1 | hsa-miR-429 | SHCBP1 |
| MYCNOS | hsa-miR-183 | CCNB1 |
| MAGI2-AS3 | hsa-miR-429 | SHCBP1 |
| RBMS3-AS3 | hsa-miR-96 | SLC1A1 |
| RBMS3-AS3 | hsa-miR-182 | THBS1 |
| CLRN1-AS1 | hsa-miR-429 | SHCBP1 |
| KLHL6-AS1 | hsa-miR-96 | SLC1A1 |
| AC073352.1 | hsa-miR-96 | SLC1A1 |
| AC073352.1 | hsa-miR-182 | THBS1 |
| CRNDE | hsa-miR-183 | CCNB1 |
| LINC00491 | hsa-miR-429 | SHCBP1 |
| RMST | hsa-miR-96 | SLC1A1 |
| RMST | hsa-miR-182 | THBS1 |
| RMST | hsa-miR-429 | SHCBP1 |
| AL139385.1 | hsa-miR-183 | CCNB1 |
| AP002478.1 | hsa-miR-182 | CHL1 |
| LINC00221 | hsa-miR-182 | CHL1 |
| TCL6 | hsa-miR-182 | CHL1 |
| UCA1 | hsa-miR-182 | CHL1 |
| LINC00114 | hsa-miR-182 | CHL1 |
| SFTA1P | hsa-miR-182 | CHL1 |
| PCA3 | hsa-miR-182 | CHL1 |
| ERVMER61-1 | hsa-miR-182 | CHL1 |
| RBMS3-AS3 | hsa-miR-182 | CHL1 |
| AC073352.1 | hsa-miR-182 | CHL1 |
| RMST | hsa-miR-182 | CHL1 |
| LINC00221 | hsa-miR-96 | PROK2 |
| TCL6 | hsa-miR-96 | PROK2 |
| UCA1 | hsa-miR-96 | PROK2 |
| LINC00114 | hsa-miR-96 | PROK2 |
| PCA3 | hsa-miR-96 | PROK2 |
| C14orf144 | hsa-miR-96 | PROK2 |
| ERVMER61-1 | hsa-miR-96 | PROK2 |
| AL161645.1 | hsa-miR-96 | PROK2 |
| RBMS3-AS3 | hsa-miR-96 | PROK2 |
| KLHL6-AS1 | hsa-miR-96 | PROK2 |
| AC073352.1 | hsa-miR-96 | PROK2 |
| RMST | hsa-miR-96 | PROK2 |
