# Supplementary material for: Construction and comprehensive analysis of a ceRNA network to reveal potential prognostic biomarkers for hepatocellular carcinoma
Source: Cancer Cell Int. 2019 Apr 11;19:90. doi: 10.1186/s12935-019-0817-y (PMC6458652; doi:10.1186/s12935-019-0817-y)
Supplement: Supplementary file 5 — Additional file 5: Table S5. The connection degree of each gene in the ceRNA network. [file 12935_2019_817_MOESM5_ESM.docx]

**Table S5. The connection degree of each gene in the ceRNA network.**

| **Gene** | **Type** | **Connection degree** |
| --- | --- | --- |
| LINC00114 | lncRNA | 3 |
| RMST | lncRNA | 3 |
| TCL6 | lncRNA | 3 |
| AC073352.1 | lncRNA | 2 |
| ERVMER61-1 | lncRNA | 2 |
| LINC00221 | lncRNA | 2 |
| PCA3 | lncRNA | 2 |
| RBMS3-AS3 | lncRNA | 2 |
| UCA1 | lncRNA | 2 |
| AC024563.1 | lncRNA | 1 |
| AC087392.1 | lncRNA | 1 |
| AL110292.1 | lncRNA | 1 |
| AL139385.1 | lncRNA | 1 |
| AL161645.1 | lncRNA | 1 |
| AL357153.1 | lncRNA | 1 |
| AP002478.1 | lncRNA | 1 |
| C14orf144 | lncRNA | 1 |
| CLRN1-AS1 | lncRNA | 1 |
| CRNDE | lncRNA | 1 |
| DLX6-AS1 | lncRNA | 1 |
| KLHL6-AS1 | lncRNA | 1 |
| LINC00491 | lncRNA | 1 |
| MAGI2-AS3 | lncRNA | 1 |
| MYCNOS | lncRNA | 1 |
| PART1 | lncRNA | 1 |
| SFTA1P | lncRNA | 1 |
| hsa-miR-96 | miRNA | 14 |
| hsa-miR-182 | miRNA | 13 |
| hsa-miR-429 | miRNA | 10 |
| hsa-miR-183 | miRNA | 7 |
| CCNB1 | mRNA | 1 |
| CHL1 | mRNA | 1 |
| PROK2 | mRNA | 1 |
| SHCBP1 | mRNA | 1 |
| SLC1A1 | mRNA | 1 |
| THBS1 | mRNA | 1 |
