# Supplementary material for: Construction and comprehensive analysis of a ceRNA network to reveal potential prognostic biomarkers for hepatocellular carcinoma
Source: Cancer Cell Int. 2019 Apr 11;19:90. doi: 10.1186/s12935-019-0817-y (PMC6458652; doi:10.1186/s12935-019-0817-y)
Supplement: Supplementary file 6 — Additional file 6: Table S6. Eighteen DElncRNAs were associated with the overall survival of patients with HCC in the TCGA HCC cohort. [file 12935_2019_817_MOESM6_ESM.docx]

**Table S6. Eighteen DElncRNAs were associated with the overall survival of patients with HCC in the TCGA HCC cohort.**

| **Gene** | **Group** | **Expression level** | **Number of patients** | **Mean survival time** | **P-value** | **Hazard ratio** |
| --- | --- | --- | --- | --- | --- | --- |
| AP002478.1 | high | >3.82533500791294 | 44 | 2.429859989 | 2.04E-07 | 3.059653 |
|  | low | <=3.82533500791294 | 323 | 5.408120451 | 2.04E-07 | 3.059653 |
| ERVMER61-1 | high | >2.51578011712588 | 52 | 2.880886416 | 6.43E-06 | 2.501411 |
|  | low | <=2.51578011712588 | 315 | 5.558069457 | 6.43E-06 | 2.501411 |
| LINC00221 | high | >8.66069212596219 | 59 | 3.222240745 | 0.000109 | 2.187611 |
|  | low | <=8.66069212596219 | 308 | 5.37701627 | 0.000109 | 2.187611 |
| TCL6 | high | >3.57919546581697 | 105 | 3.912734014 | 0.000308 | 1.913216 |
|  | low | <=3.57919546581697 | 262 | 5.489857556 | 0.000308 | 1.913216 |
| LINC00491 | high | >1.15139350176246 | 111 | 4.023592524 | 0.000355 | 1.895757 |
|  | low | <=1.15139350176246 | 256 | 5.491654274 | 0.000355 | 1.895757 |
| AL139385.1 | high | >3.85184702943292 | 204 | 5.723853788 | 0.000386 | 0.535553 |
|  | low | <=3.85184702943292 | 163 | 4.301202124 | 0.000386 | 0.535553 |
| CLRN1-AS1 | high | >0.000116853565806475 | 285 | 5.559769989 | 0.00103 | 0.539046 |
|  | low | <=0.000116853565806475 | 82 | 3.716239769 | 0.00103 | 0.539046 |
| CRNDE | high | >7.79813644253741 | 96 | 3.806791553 | 0.001085 | 1.818381 |
|  | low | <=7.79813644253741 | 271 | 5.630044769 | 0.001085 | 1.818381 |
| AL161645.1 | high | >4.12000661861749 | 141 | 6.131821252 | 0.001361 | 0.535028 |
|  | low | <=4.12000661861749 | 226 | 4.49332153 | 0.001361 | 0.535028 |
| AC073352.1 | high | >2.2333386607888 | 168 | 4.287982915 | 0.001434 | 1.751964 |
|  | low | <=2.2333386607888 | 199 | 5.781993569 | 0.001434 | 1.751964 |
| PART1 | high | >5.97714851708026 | 41 | 3.850444808 | 0.00187 | 2.106135 |
|  | low | <=5.97714851708026 | 326 | 5.290988845 | 0.00187 | 2.106135 |
| C14orf144 | high | >3.98100122937484 | 41 | 6.75210158 | 0.006299 | 0.381597 |
|  | low | <=3.98100122937484 | 326 | 4.840208363 | 0.006299 | 0.381597 |
| RBMS3-AS3 | high | >1.53646311105413 | 128 | 5.780588853 | 0.010876 | 0.607958 |
|  | low | <=1.53646311105413 | 239 | 4.733798785 | 0.010876 | 0.607958 |
| LINC00114 | high | >0.0266005153328426 | 288 | 4.515978698 | 0.0114 | 1.951531 |
|  | low | <=0.0266005153328426 | 79 | 6.531563103 | 0.0114 | 1.951531 |
| MYCNOS | high | >4.1305491943903 | 38 | 3.419900731 | 0.01352 | 1.892211 |
|  | low | <=4.1305491943903 | 329 | 5.254543246 | 0.01352 | 1.892211 |
| PCA3 | high | >0.0783978016909701 | 91 | 6.443406508 | 0.013718 | 0.574131 |
|  | low | <=0.0783978016909701 | 276 | 4.579545614 | 0.013718 | 0.574131 |
| DLX6-AS1 | high | >5.83533655069451 | 42 | 3.209269257 | 0.018431 | 1.792667 |
|  | low | <=5.83533655069451 | 325 | 5.300540284 | 0.018431 | 1.792667 |
| AL357153.1 | high | >0 | 266 | 5.529627227 | 0.038866 | 0.678938 |
|  | low | <=0 | 101 | 4.15083378 | 0.038866 | 0.678938 |
