# Supplementary material for: Construction and comprehensive analysis of a ceRNA network to reveal potential prognostic biomarkers for hepatocellular carcinoma
Source: Cancer Cell Int. 2019 Apr 11;19:90. doi: 10.1186/s12935-019-0817-y (PMC6458652; doi:10.1186/s12935-019-0817-y)
Supplement: Supplementary file 7 — Additional file 7: Table S7. Six DEmRNAs were associated with the overall survival of patients with HCC in the TCGA HCC cohort. [file 12935_2019_817_MOESM7_ESM.docx]

**Table S7.** **Six DEmRNAs were associated with the overall survival of patients with HCC in the TCGA HCC cohort.**

| **Gene** | **Group** | **Expression level** | **Number of patients** | **Mean survival time** | **P-value** | **Hazard ratio** |
| --- | --- | --- | --- | --- | --- | --- |
| CCNB1 | high | >10.9221368585067 | 48 | 2.616392202 | 4.90E-08 | 3.152365 |
|  | low | <=10.9221368585067 | 319 | 5.519796591 | 4.90E-08 | 3.152365 |
| PROK2 | high | >0.0677250846427177 | 272 | 5.503419133 | 0.000295549 | 0.514688 |
|  | low | <=0.0677250846427177 | 95 | 3.776723433 | 0.000295549 | 0.514688 |
| SHCBP1 | high | >6.10439832448332 | 250 | 4.755338788 | 0.000337388 | 2.141284 |
|  | low | <=6.10439832448332 | 117 | 5.403938264 | 0.000337388 | 2.141284 |
| CHL1 | high | >2.80095611543639 | 177 | 4.299461567 | 0.01217957 | 1.559586 |
|  | low | <=2.80095611543639 | 190 | 5.749605314 | 0.01217957 | 1.559586 |
| SLC1A1 | high | >11.8779671450615 | 47 | 6.348225702 | 0.017114841 | 0.477969 |
|  | low | <=11.8779671450615 | 320 | 4.959350051 | 0.017114841 | 0.477969 |
| THBS1 | high | >9.4613891370706 | 323 | 5.280449751 | 0.018140101 | 0.564864 |
|  | low | <=9.4613891370706 | 44 | 3.546838029 | 0.018140101 | 0.564864 |
