# Supplementary material for: Construction and comprehensive analysis of a ceRNA network to reveal potential prognostic biomarkers for hepatocellular carcinoma
Source: Cancer Cell Int. 2019 Apr 11;19:90. doi: 10.1186/s12935-019-0817-y (PMC6458652; doi:10.1186/s12935-019-0817-y)
Supplement: Supplementary file 8 — Additional file 8: Table S8. Seven DElncRNAs were associated with the overall survival of patients with HCC in the meta-GEO HCC cohort. [file 12935_2019_817_MOESM8_ESM.docx]

**Table S8.** **Seven DElncRNAs were associated with the overall survival of patients with HCC in the meta-GEO HCC cohort.**

| **Gene** | **Group** | **Expression level** | **Number of patients** | **Mean survival time** | **P-value** | **Hazard ratio** |
| --- | --- | --- | --- | --- | --- | --- |
| CRNDE | high | >7.58901546613848 | 64 | 2.020933 | 1.81E-07 | 2.816576 |
|  | low | <=7.58901546613848 | 129 | 3.991893 | 1.81E-07 | 2.816576 |
| UCA1 | high | >5.87248214333145 | 148 | 3.692593 | 5.78E-07 | 0.348634 |
|  | low | <=5.87248214333145 | 45 | 1.939478 | 5.78E-07 | 0.348634 |
| SFTA1P | high | >5.03837210529714 | 79 | 2.340767 | 0.000669 | 1.969351 |
|  | low | <=5.03837210529714 | 114 | 3.852487 | 0.000669 | 1.969351 |
| LINC00221 | high | >3.89645468566715 | 159 | 2.731477 | 0.000902 | 3.706125 |
|  | low | <=3.89645468566715 | 34 | 5.875966 | 0.000902 | 3.706125 |
| PART1 | high | >4.52326685396817 | 108 | 3.33233 | 0.002302 | 0.54142 |
|  | low | <=4.52326685396817 | 85 | 2.546121 | 0.002302 | 0.54142 |
| DLX6-AS1 | high | >3.88368561298656 | 168 | 2.894685 | 0.00869 | 2.878228 |
|  | low | <=3.88368561298656 | 25 | 3.809219 | 0.00869 | 2.878228 |
| MYCNOS | high | >4.99702286338009 | 165 | 2.774555 | 0.029934 | 1.94191 |
|  | low | <=4.99702286338009 | 28 | 4.476189 | 0.029934 | 1.94191 |
