# Supplementary material for: Construction and comprehensive analysis of a ceRNA network to reveal potential prognostic biomarkers for hepatocellular carcinoma
Source: Cancer Cell Int. 2019 Apr 11;19:90. doi: 10.1186/s12935-019-0817-y (PMC6458652; doi:10.1186/s12935-019-0817-y)
Supplement: Supplementary file 9 — Additional file 9: Table S9. Five DEmRNAs were associated with the overall survival of patients with HCC in the meta-GEO HCC cohort. [file 12935_2019_817_MOESM9_ESM.docx]

**Table S9.** **Five DEmRNAs were associated with the overall survival of patients with HCC in the meta-GEO HCC cohort.**

| **Gene** | **Group** | **Expression level** | **Number of patients** | **Mean survival time** | **P-value** | **Hazard ratio** |
| --- | --- | --- | --- | --- | --- | --- |
| CCNB1 | high | >8.80589637822446 | 45 | 1.582333 | 5.22E-09 | 3.448443 |
|  | low | <=8.80589637822446 | 148 | 3.566619 | 5.22E-09 | 3.448443 |
| SHCBP1 | high | >9.76767523713985 | 29 | 1.437037 | 3.25E-08 | 3.502495 |
|  | low | <=9.76767523713985 | 164 | 3.460509 | 3.25E-08 | 3.502495 |
| PROK2 | high | >6.41324851104975 | 21 | 1.477366 | 3.55E-05 | 3.031454 |
|  | low | <=6.41324851104975 | 172 | 3.323052 | 3.55E-05 | 3.031454 |
| THBS1 | high | >8.30059223662092 | 81 | 2.324701 | 0.010259 | 1.680111 |
|  | low | <=8.30059223662092 | 112 | 3.5817 | 0.010259 | 1.680111 |
| CHL1 | high | >5.00138055023808 | 20 | 2.018503 | 0.021825 | 1.899518 |
|  | low | <=5.00138055023808 | 173 | 3.267406 | 0.021825 | 1.899518 |
