# Supplementary material for: Construction and comprehensive analysis of a ceRNA network to reveal potential prognostic biomarkers for hepatocellular carcinoma
Source: Cancer Cell Int. 2019 Apr 11;19:90. doi: 10.1186/s12935-019-0817-y (PMC6458652; doi:10.1186/s12935-019-0817-y)
Supplement: Supplementary file 10 — Additional file 10: Table S10. Three DEmiRNAs were associated with the overall survival of patients with HCC in the TCGA HCC cohort. [file 12935_2019_817_MOESM10_ESM.docx]

**Table S10.** **Three DEmiRNAs were associated with the overall survival of patients with HCC in the TCGA HCC cohort.**

| **Gene** | **Group** | **Expression level** | **Number of patients** | **Mean survival time** | **P-value** | **Hazard ratio** |
| --- | --- | --- | --- | --- | --- | --- |
| hsa-miR-182 | high | >16.1082936550519 | 80 | 3.568842 | 0.010638 | 1.636928 |
|  | low | <=16.1082936550519 | 291 | 5.626684 | 0.010638 | 1.636928 |
| hsa-miR-183 | high | >15.4948705260225 | 39 | 3.231687 | 0.020557 | 1.750698 |
|  | low | <=15.4948705260225 | 332 | 5.44482 | 0.020557 | 1.750698 |
| hsa-miR-429 | high | >5.80695629414192 | 132 | 3.934265 | 0.032843 | 1.471878 |
|  | low | <=5.80695629414192 | 239 | 5.656183 | 0.032843 | 1.471878 |
