# Supplementary material for: Construction and comprehensive analysis of a ceRNA network to reveal potential prognostic biomarkers for hepatocellular carcinoma
Source: Cancer Cell Int. 2019 Apr 11;19:90. doi: 10.1186/s12935-019-0817-y (PMC6458652; doi:10.1186/s12935-019-0817-y)
Supplement: Supplementary file 11 — Additional file 11: Table S11. Gene enrichment in the high CCNB1 expression group of patients with HCC in the TCGA HCC cohort. [file 12935_2019_817_MOESM11_ESM.docx]

**Table S11. Gene enrichment in the high CCNB1 expression group of patients with HCC in the TCGA HCC cohort.**

| **Name** | **Size** | **ES** | **NES** | **NOM P-value** | **FDR q-value** | **FWER P-value** | **Rank at max** | **Leading edge** |
| --- | --- | --- | --- | --- | --- | --- | --- | --- |
| SISTER_CHROMATID_SEGREGATION | 161 | 0.686378 | 2.321292 | 0 | 4.76E-04 | 0.003 | 1420 | tags=40%, list=8%, signal=44% |
| NUCLEAR_CHROMOSOME_SEGREGATION | 200 | 0.658225 | 2.305384 | 0 | 5.20E-04 | 0.003 | 1460 | tags=36%, list=8%, signal=39% |
| DNA_REPLICATION | 192 | 0.625041 | 2.22794 | 0 | 5.22E-04 | 0.008 | 1560 | tags=40%, list=9%, signal=43% |
| DNA_RECOMBINATION | 185 | 0.615521 | 2.324801 | 0 | 6.35E-04 | 0.003 | 1284 | tags=34%, list=7%, signal=36% |
| CHROMOSOME_SEGREGATION | 243 | 0.645212 | 2.343508 | 0 | 0.00121 | 0.002 | 1460 | tags=35%, list=8%, signal=37% |
| CELL_CYCLE_G1_S_PHASE_TRANSITION | 107 | 0.612469 | 2.095513 | 0 | 0.002658 | 0.084 | 1788 | tags=44%, list=10%, signal=49% |
