# Supplementary material for: Construction and comprehensive analysis of a ceRNA network to reveal potential prognostic biomarkers for hepatocellular carcinoma
Source: Cancer Cell Int. 2019 Apr 11;19:90. doi: 10.1186/s12935-019-0817-y (PMC6458652; doi:10.1186/s12935-019-0817-y)
Supplement: Supplementary file 12 — Additional file 12: Table S12. Gene enrichment in the high SHCBP1 expression group of patients with HCC in the TCGA HCC cohort. [file 12935_2019_817_MOESM12_ESM.docx]

**Table S12. Gene enrichment in the high SHCBP1 expression group of patients with HCC in the TCGA cohort.**

| **Name** | **Size** | **ES** | **NES** | **NOM P-value** | **FDR q-value** | **FWER P-value** | **Rank at max** | **Leading edge** |
| --- | --- | --- | --- | --- | --- | --- | --- | --- |
| REGULATION_OF_NUCLEAR_DIVISION | 149 | 0.627891 | 2.562431 | 0 | 0 | 0 | 525 | tags=26%, list=3%, signal=27% |
| ORGANELLE_FISSION | 441 | 0.600468 | 2.491241 | 0 | 0 | 0 | 1693 | tags=34%, list=10%, signal=37% |
| MEIOTIC_CELL_CYCLE_PROCESS | 123 | 0.639438 | 2.381718 | 0 | 4.27E-04 | 0.001 | 2623 | tags=45%, list=15%, signal=52% |
| CHROMOSOME_SEGREGATION | 243 | 0.671936 | 2.451861 | 0.002008 | 5.86E-04 | 0.001 | 967 | tags=37%, list=6%, signal=39% |
| SISTER_CHROMATID_SEGREGATION | 161 | 0.720685 | 2.419518 | 0.002008 | 6.22E-04 | 0.001 | 1940 | tags=50%, list=11%, signal=56% |
| MEIOTIC_CELL_CYCLE | 153 | 0.64013 | 2.447433 | 0 | 6.84E-04 | 0.001 | 2623 | tags=44%, list=15%, signal=52% |
| NUCLEAR_CHROMOSOME_SEGREGATION | 200 | 0.699656 | 2.4537 | 0.002004 | 6.84E-04 | 0.001 | 1940 | tags=47%, list=11%, signal=52% |
| CELL_CYCLE_PHASE_TRANSITION | 247 | 0.613894 | 2.454474 | 0 | 8.20E-04 | 0.001 | 1658 | tags=38%, list=10%, signal=41% |
| MITOTIC_NUCLEAR_DIVISION | 336 | 0.622454 | 2.459165 | 0.002041 | 0.001026 | 0.001 | 1693 | tags=36%, list=10%, signal=39% |
| DNA_REPLICATION | 192 | 0.660112 | 2.292444 | 0.002033 | 0.001258 | 0.009 | 1439 | tags=43%, list=8%, signal=47% |
| DNA_RECOMBINATION | 185 | 0.619867 | 2.29438 | 0 | 0.001345 | 0.009 | 1792 | tags=38%, list=10%, signal=42% |
| CELL_CYCLE_G1_S_PHASE_TRANSITION | 107 | 0.668273 | 2.273184 | 0 | 0.001403 | 0.01 | 2089 | tags=50%, list=12%, signal=57% |
